# Modeling the Distribution of *Geodia* Sponges and Sponge Grounds in the Northwest Atlantic

**DOI:** 10.1371/journal.pone.0082306

**Published:** 2013-12-04

**Authors:** Anders Knudby, Ellen Kenchington, Francisco Javier Murillo

**Affiliations:** 1 Department of Geography, Simon Fraser University, Burnaby, British Columbia, Canada; 2 Bedford Institute of Oceanography, Department of Fisheries and Oceans, Dartmouth, Nova Scotia, Canada; 3 Instituto Español de Oceanografía, Centro Oceanográfico de Vigo, Programa de Pesquerías Lejanas, Vigo, Spain,; University of Genova, Italy, Italy

## Abstract

Deep-sea sponge grounds provide structurally complex habitat for fish and invertebrates and enhance local biodiversity. They are also vulnerable to bottom-contact fisheries and prime candidates for Vulnerable Marine Ecosystem designation and related conservation action. This study uses species distribution modeling, based on presence and absence observations of *Geodia* spp. and sponge grounds derived from research trawl catches, as well as spatially continuous data on the physical and biological ocean environment derived from satellite data and oceanographic models, to model the distribution of *Geodia* sponges and sponge grounds in the Northwest Atlantic. Most models produce excellent fits with validation data although fits are reduced when models are extrapolated to new areas, especially when oceanographic regimes differ between areas. Depth and minimum bottom salinity were important predictors in most models, and a *Geodia* spp. minimum bottom salinity tolerance threshold in the 34.3-34.8 psu range was hypothesized on the basis of model structure. The models indicated two currently unsampled regions within the study area, the deeper parts of Baffin Bay and the Newfoundland and Labrador slopes, where future sponge grounds are most likely to be found.

## Introduction

Sponges form an ancient group of sessile filter-feeders characterized by a body plan built around a system of canals through which water is pumped, supplying food and oxygen and removing waste [1]. In the deep sea, sponges enhance both local nutrient and energy exchange [2] and biodiversity [3-5]. As such they constitute an important component of benthic ecosystems, especially when they occur in dense aggregations known as sponge grounds (alternatively “sponge fields” or “ostur”) that provide a structurally complex habitat for fish and invertebrates [6-8]. Due to the inaccessibility of their habitats relatively little is known about deep-sea sponges, and most research has focused on the anatomy and taxonomy of trawl-caught specimens [1,9], their biology and associated fauna [3], their broad-scale geographic distribution [10-12], and the potential of their secondary metabolites for drug development [13-15]. More recently the threat posed by bottom-contact fisheries has sparked conservation concern, especially in light of deep-sea sponges being slow-growing and long-lived organisms vulnerable to disturbance [3,16]. The combination of this vulnerability and their importance for deep-sea benthic ecosystems makes sponge grounds a prime candidate for Vulnerable Marine Ecosystem (VME) designation as outlined in United Nations General Assembly Resolution 61/105 [17], and hence subject to conservation action [18]. Because deep-sea conservation of benthic species and habitats is primarily carried out through spatial fisheries management there is a need to map the distributions of sponges and sponge grounds at the level of spatial detail needed for such management.

Knowledge of the spatial distribution of deep-sea sponge species and sponge grounds is currently largely limited to aggregations of point observations derived from a variety of sources, including research trawls [12], the local knowledge of fishermen [10,19], or direct observations using SCUBA, remotely operated vehicles (ROVs) or manned submersibles [20]. Although such data occasionally provide dense sampling coverage of an area [11,12], they are more often limited in both spatial extent and sampling density. Species distribution modeling (SDM), that is the quantification and extrapolation of species-environment relationships [21], can be employed to produce spatially continuous distribution maps for use in spatial fisheries management. Despite its utility in data sparse situations, SDM has rarely been used in the deep sea except to map distributions of the cold-water coral *Lophelia pertusa* [22-25] and its reefs [26], and we are not aware of any existing SDM studies involving deep-sea sponges.

In this study we used a data set obtained from recording the sponge catch in large research trawls, and to a lesser extent rock dredges and underwater video, to explore the utility of SDM-based mapping of deep-sea sponges and sponge grounds and produce distribution maps for the structure-forming sponge species *Geodia barretti* and *Geodia phlegraei*, the *Geodia* genus, and *Geodia*-dominated sponge grounds in the northwest Atlantic Ocean (NWA). We compared the structure and predictions of models developed from four subsets of our study area, each with a distinct cluster of sponges and sponge grounds and oceanographic setting, and assessed the potential for extrapolation of predictions to unsampled areas within and among those domains and to other unsampled areas in the NWA. The mapped predictions provide the best estimate of the current distribution of sponge grounds and *Geodia* spp. sponges in the NWA.

## Study Area

**Figure 1:**
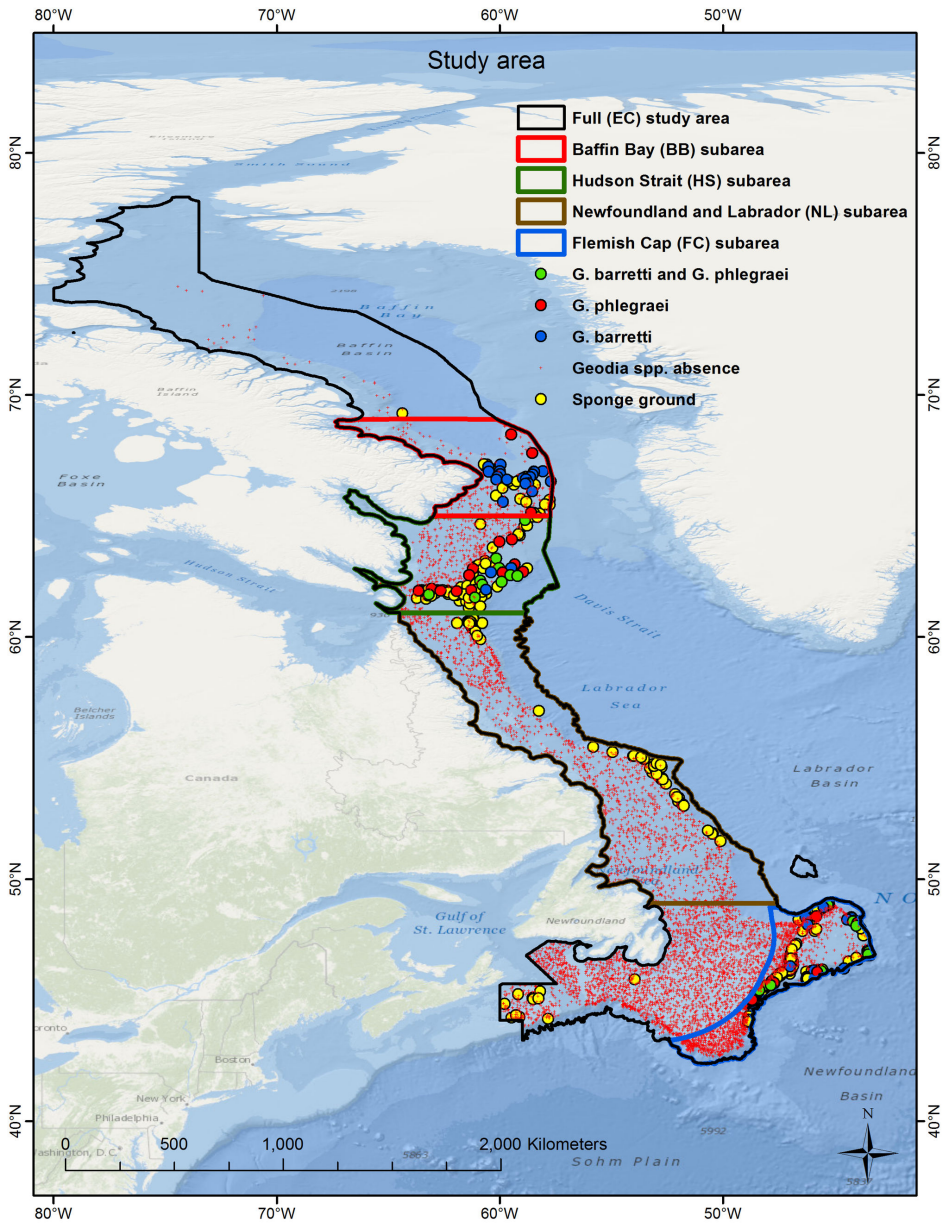
Study area. Illustration of the full study area (EC) and the four subareas (BB, HS, NL and FC), showing the locations of presence/absence data on *Geodia* spp. and sponge grounds with unidentified species. Most of the data records are from research vessel trawl surveys conducted by Canada and the EU-Spain. All maps were created using ArcGIS® 10.1 software and its Ocean Basemap.

Definition of the study area (Figure 1) was influenced by the availability of research trawl data, and includes the Canadian continental shelf and upper slope in the NWA, from the area off Cape Breton, Nova Scotia in the south, to southern Ellesmere Island, Nunavut in the north (henceforth East Coast, EC). Specifically the area is bounded in the north by Zone 0A of the Northwest Atlantic Fisheries Organization (NAFO), in the south by NAFO zones 4Vs and 3Ps, in the west at a distance of 20 km from the Canadian coast, and in the east by the 2500 m depth contour or the eastern boundary of NAFO Zones 0A and 0B, whichever is shallower. The area does not extend into Lancaster Sound or the Hudson Strait beyond the limits of NAFO Zone 0A and NAFO Zones 0B/2G respectively. Within this area, four smaller subareas were defined based on oceanography and data availability.

The first subarea includes the Flemish Cap and the nose and tail of Grand Bank east of Newfoundland, Canada (henceforth Flemish Cap, FC), bounded by the Canadian EEZ in the west and by the 2500 m depth contour at the continental slope. The second subarea includes primarily the Canadian continental shelf from the Hudson Strait (61° N) to the sill that separates the Labrador and Baffin basins (65° N) (henceforth Hudson Strait, HS), and the third subarea lies immediately to the north thereof, from the sill (65° N) to the southern Baffin Basin (69° N) (henceforth Baffin Basin, BB); both are bounded at distances of 20 km from the Canadian coast in the west and NAFO zones 0A and 0B in the east, and form part of the Eastern Arctic bioregion [27]. Sponge grounds in the FC, HS and BB subareas are dominated by *Geodia* spp. sponges [11,12]. The fourth subarea includes the Newfoundland and Labrador shelves and slopes from central Newfoundland (49 ° N) to the Hudson Strait (61° N) (henceforth Newfoundland and Labrador, NL); it is bounded at a distance of 20 km from the Canadian coast in the west and NAFO zones 0A and 0B in the east, and corresponds roughly to the northern 2/3 of the Newfoundland-Labrador Shelves bioregion [27]. *Geodia* spp. presence at sponge grounds identified in NL is known [19] but could not be confirmed specifically in our data because species/genus-level identification was not performed for those sponge catches. Delineation of these four subareas leaves two areas within the full study area: the remainder of Grand Bank with a portion of the western Scotian Shelf, and the westerly portion of Baffin Basin (Figure 1). 

## Data

The data used in this study include georeferenced field observations of sponge species and sponge ground presence/absence acquired from Canadian and European Union-Spanish (EU-Spain) research trawl surveys, box-cores/dredges and in-situ camera/video recordings collected by the authors, as well as spatially continuous environmental information derived from satellite data and oceanographic model outputs. Permission for all scientific surveys in international waters were given by NAFO, and all data collected from the Canadian research vessel surveys were undertaken with permission of the Department of Fisheries and Oceans, Canada. All field observations are restricted to depths <1800 m.

### Sponge grounds

The United Nations General Assembly Resolution 61/105 [17] calls for the protection of vulnerable marine ecosystems, which have been interpreted as including habitat-forming species such as “significant concentrations” of “some sponge dominated communities” [28]. Although consensus exists regarding a qualitative difference between sponge grounds and areas with lower-density presence of sponges, most published definitions of sponge grounds are not quantitative in nature; examples include “a restricted area where large-sized sponges are strikingly common” [10], and “aggregations of large sponges that develop under certain geological, hydrological and biological conditions to form structural habitat” [16]. This makes it difficult to objectively determine whether a sponge ground is present in a given location regardless of the available information. Quantitative definitions do exist, e.g. Klitgaard et al. [29] defined ‘ostur’ as areas where 90% of the wet weight of non-fish trawl catches is comprised of sponges, and Kenchington et al. [30] used a kernel density approach to delineate areas covered by research trawl catches above a range of weight thresholds and used an abrupt change in the area covered by catches above two neighbouring threshold values to indicate the difference between sponge grounds and lower-density sponge presence for a local area. This approach, as well as its results for individual trawl gear types and bioregions in the northwest Atlantic Ocean [31], is adopted in this study.

Sponge ground presence/absence data were collected via research vessel trawl surveys conducted by the Canadian Department of Fisheries and Oceans (DFO) and the Spanish Institute of Oceanography (IEO) in collaboration with other European institutions from 2007 to 2011.

Research trawl catches were, as a minimum, separated and weighed by phylum to yield a measure of the total wet weight of caught sponges, and quantified as kg/standard 1 km tow. In order to separate sponge grounds from non-sponge ground locations, a threshold was applied to this wet weight; locations with catches above the threshold were classified as sponge ground presence and others as sponge ground absence. Different gear and trawl durations were used by the different surveys, leading to differences in the area swept by each research trawl as well as differences in the proportion of the swept benthic fauna that was recovered and available for weighing on deck (trawl catchability/efficiency). In order to use the entire data set in our study, separate thresholds previously developed for each gear type and biogeographic region using kernel density analyses were applied to the respective data points (Table 1) to define sponge grounds in each subarea [11,30].

**Table 1:** Local gear-specific thresholds used to define sponge ground presence and absence.

**Subarea**	**Gear type**	**Local threshold (kg sponge/tow)**	**Sponge ground presences**	**Sponge ground absences**
FC	Campelen 1800 & Lofoten	70	150	3455
NL	Campelen 1800	200	150	2191
HS	Cosmos	40	89	938
	Alfredo	70		
	Campelen 1800	40		
BB	Cosmos	40	28	519
	Alfredo	70		
	Campelen 1800	40		

### Sponge species

The catch from each research trawl in the EC study area was identified to the level of phylum, genus or species, depending on the taxonomic expertise available. Sponges can be difficult to identify without examining the spicules microscopically, and consequently catches from the NL subarea were only identified to phylum, along with catches in the wider EC outside of FC, HS and BB subareas. 

For the FC study subarea, genus/species level identification of catches from EU-Spain fisheries surveys (2007) and dredges from the Spanish-led NAFO NEREIDA missions in (2009 and 2010) was performed by one of us (JM); this identification is ongoing with additional records expected over the coming years. Two additional *Geodia* spp. identifications were obtained in 2010 during a DFO-led NEREIDA mission using the ROV ROPOS, for a total of 41 *Geodia* spp. presence locations with genus/species-level identification in the FC subarea and 1632 *Geodia* spp. absences. Seven different *Geodia* species were identified: *G. barretti*, *G. phlegraei*, *G. hentscheli*, *G. atlantica*, *G. nodastrella*, *G. parva* and *G. macandrewii*, following the most recent taxonomy [9]. However *G. phlegraei* and *G. parva* are very closely related and we could not distinguish them with confidence in our data. As *G. parva* was only recently resurrected as a valid species from a subspecies of *G. phlegraei* [9] we refer to our *G. parva* specimens as *G. phlegraei* hereafter. Of these, only *G. barretti* and *G. phlegraei* were recorded in sufficient numbers (here >15) to attempt species-level distribution modeling. *Geodia* spp. absences were compiled from DFO and IEO fisheries survey data. These surveys do not routinely identify sponges beyond phylum level, so only catches with no sponges of any kind were used as *Geodia* spp. absences. 

The HS and BB subareas were covered exclusively by research trawl surveys from 2010 and 2011 conducted by the Central and Arctic Region of DFO (Winnipeg, MB, Canada). Some catches from these surveys were identified to species level by Megan Best (DFO, Bedford Institute of Oceanography, Dartmouth, NS, Canada), for a total of 47 *Geodia* spp. presences and 788 *Geodia* spp. absences, the latter being locations with no sponges of any kind in the catch as for the FC. Although the research trawls that produced these data covered areas as determined by the gear type and the duration of each trawl, all field data are treated as point observations in this study. The geographic coordinate associated with each trawl was obtained at the trawl start position; for simplicity this location is used in the analysis to represent the entire trawl.

### Environmental data

Given very limited knowledge of the habitat requirements and ecology of the sponge species in question, as well as the environmental conditions that may enable or limit the formation of sponge grounds, environmental data layers used as predictors of sponge species and sponge ground distributions were selected on the basis of availability and general notions of relevance, as is not uncommon in exploratory SDM applications [32]. Data on a total of 42 predictors were compiled from different sources (Table 2), and transformed as necessary to geographic coordinates using the WGS 1984 datum and a 0.017° cell size (approximately equal to a 1 km cell size in the FC subarea).

**Table 2:** Environmental data layers used to quantify aspects of bathymetry, surface chlorophyll-*a* concentration, temperature, salinity and current at the sea surface and sea floor, and shear near the sea floor.

**All variables**	**Unit**	**Quantifications**	**Data source**	**Native resolution**
Depth	m	N/A	GEBCO	30”
Slope	degrees	N/A	GEBCO	30”
Annual chlorophyll-*a*	mg m^-3^	Range, Min, Mean, Max	OceanColor	9 km
Summer chlorophyll-*a*	mg m^-3^	Range, Min, Mean, Max	OceanColor	9 km
Fall chlorophyll-*a*	mg m^-3^	Range, Min, Mean, Max	OceanColor	9 km
Surface temperature	°C	Range, Min, Mean, Max	GLORYS	¼°
Bottom temperature	°C	Range, Min, Mean, Max	GLORYS	¼°
Surface salinity	PSU	Range, Min, Mean, Max	GLORYS	¼°
Bottom salinity	PSU	Range, Min, Mean, Max	GLORYS	¼°
Surface current	m/s	Range, Min, Mean, Max	GLORYS	¼°
Bottom current	m/s	Range, Min, Mean, Max	GLORYS	¼°
Shear	Pa	Range, Min, Mean, Max	GLORYS	¼°

#### Seafloor depth and slope

The depth and slope of the seafloor were derived from the 30 arc-second General Bathymetric Chart of the Oceans (GEBCO) database [33]. Depth can act as a proxy for unmeasured predictors such as historical trawling intensity in addition to being correlated with variables such as temperature and salinity limits and ranges. Slope may act as a proxy for substrate type (e.g. rock, sand, silt, mud), which is thought to influence the ability of sponge larvae or fragments to settle in an area.

#### Sea surface chlorophyll-a concentration

Data on sea surface chlorophyll-*a* concentrations were derived from publicly available Level 3 SeaWiFS data for the period January 2001 – December 2010. These data are spatially composited to a 9 km cell size and provided as monthly and annual mean values [34]. Annual minimum, maximum, mean and range statistics were calculated for each cell from the 10 annual data layers. Similarly, summer and fall minimum, maximum, mean and range statistics were calculated respectively from the March-May and June-August data layers from each of the 10 years, and all data layers were then resampled to the 0.017° cell size. Due to high latitude and low sun elevation, winter and spring data provided poor coverage of the study area and were not included. Sea surface chlorophyll-*a* concentration may be related to the export flux of particulate organic carbon [35] and thus nutrient availability at the sea floor. Seasonal rather than annual measures of nutrient availability may be relevant due to the sponges’ reproductive cycles, shown for *Geodia barretti* in the northeast Atlantic Ocean [36] where the onset of reproduction coincides with the phytoplankton bloom. There gametes are released in early summer just after the spring phytoplankton bloom is over, when organic matter sedimentation is highest. In the northern part of our study area (HS, BB), the phytoplankton bloom is initiated in late June and runs on average 8 weeks ending in mid-August [37]. In the FC region the bloom peaks towards the last two weeks of March [38]. 

#### Physical ocean variables

Data on shear stress at the seafloor, as well as temperature, salinity and current speed for the sea surface and seafloor were derived from the GLORYS2V1 ocean reanalysis at ¼ ° resolution. GLORYS2V1 provides 3-hourly estimates of these and other variables from 1993 to 2009 [39]. Minimum, maximum, mean and range statistics were calculated for each cell from these 3-hourly values and resampled to the 0.017° cell size.

## Methods

### Variable elimination

Highly correlated environmental variables were identified and eliminated [40] to reduce problems arising from a large number of collinear predictors [41] and produce interpretable models from which ecological hypotheses could be generated. The Pearson correlation coefficients between all predictors were calculated from all raster cells in the study area, and the two predictors with the highest correlation were then considered and one of them eliminated. This process was repeated until no remaining variables were highly correlated, here defined as |R|>0.5. In most cases high correlations were identified between closely related variables (i.e., the annual range of chlorophyll-a concentrations and the annual maximum chlorophyll-a concentration). When the highly correlated variables represented two metrics (mean, maximum, minimum and range) for the same variable, the mean was preferentially eliminated, then the range, and then the minimum or the maximum. Elimination of the minimum or maximum metric when these two were correlated depended on the variable in question. This preferential elimination of some metrics over others was based on the reasoning that minima and maxima may represent tolerance limits for an organism, while the range may represent the variability of conditions likely to be encountered. Annual chlorophyll-*a* concentrations were preferentially eliminated over seasonal ones, and summer chlorophyll-*a* concentrations were preferentially retained over those from the fall because of their temporal proximity to the spring seasonal reproduction peak of *Geodia barretti* as reported in Scandinavian waters [36]. Variables describing conditions at the seafloor were preferentially retained over those describing conditions at the sea surface. These variable elimination preferences are subjective, but given the limited data on the ecology of deep-sea sponges, including *Geodia* spp., no objective alternative was available. The predictors remaining after the elimination of highly correlated variables are shown in Table 3.

**Table 3:** Environmental variables remaining after variable elimination.

**Environmental variable**	**Quantification**
Depth	N/A
Slope	N/A
Summer chlorophyll-*a*	Min, Max
Fall chlorophyll-*a*	Min, Max
Bottom temperature	Max
Bottom salinity	Min
Bottom current	Min, Max

### Distribution modeling

Random forest models [42] were used to model the distribution of *Geodia* sponges for the FC, HS and BB subareas (3 predictions in each of 3 subareas), and the distribution of sponge grounds for all four subareas and the full extent (EC). Random forest is an ensemble technique based on classification trees in which each split is determined using a random subsample of the available predictors. A random forest model is trained on both presence and absence data from a common area, and is preferably used for SDM when such data are available. The resultant model can be used to predict distributions in non-sampled areas by identifying areas with similar environmental conditions. The random forest models were implemented in R, using the ‘randomForest’ package [43] with default settings. Models were developed independently for each area, and model fits were quantified as the Area Under the receiver operating Characteristic (AUC) using 10-fold cross-validation repeated 10 times. AUC values typically range from 0.5 for classifiers that perform no better than random to 1.0 for error-free classifiers [44]. Fit was quantified both when models were applied to the area for which they were developed, and when they were extrapolated to other areas. Extrapolations were performed for all combinations of the four subareas, resulting in 18 for *Geodia* sponges (models of *Geodia* spp., *G. barretti* and *G. phlegraei* for each of the FC, HS and BB subareas extrapolated to the other two subareas) and 12 for sponge grounds (models for each of the four subareas extrapolated to the other three subareas).

Ecologically meaningful interpretation of the structure of complex models such as random forest is difficult, but can be assisted by partial dependence plots and variable importance measures. Partial dependence plots were created by predicting presence probability from a model, varying a single predictor while keeping all other variables at their mean observed value. In addition, variable importance was calculated for each model by permuting test set values of each individual variable and quantifying the resulting change in model fit. The permutation is repeated 10 times for each cross-validation split to obtain results that are independent of specific cross-validation splits and specific permutations [45]. Additionally, graphical displays of important variables were used to aid interpretation.

## Results

### Model fit and structure

All models run on the subareas, except those for *G. barretti* and sponge grounds in the BB subarea, produced excellent fits (AUC >= 0.9; Table 4), and there were no consistent differences between the predictability of the two *Geodia* species, the *Geodia* genus, and the sponge ground habitat. The excellent fit of most models strongly supports the use of SDM for local mapping of both *Geodia* spp. sponges and sponge grounds, suggesting that these models are suitable for outlining sponge ground areas to be considered for VME designation.

**Table 4:** AUC values quantifying model fits for the four subareas.

**Subarea**	***Geodia barretti***	***Geodia phlegraei***	***Geodia* spp.**	**Sponge grounds**
FC	0.958	0.964	0.939	0.982
NL				0.946
HS	0.957	0.959	0.957	0.920
BB	0.531	0.900	0.913	0.795

The three most important variables in each model run on the subareas, and the reduction in AUC resulting from their permutation in the test set, are listed in Table 5. Minimum bottom salinity and depth are generally the most important variables, both listed for 11 models. Other important variables include maximum bottom temperature (4 models), maximum bottom current (3 models), minimum bottom current (2 models), as well as slope and three variations of seasonal chlorophyll-*a* concentrations (1 model). 

**Table 5:** The three most important (AUC reduction >0.01) variables in each model and the reduction in AUC values when they are permuted in the test set.

	**FC**	**NL**	**HS**	**BB**
**Response**	**Variable (AUC reduction x 10^-2^)**			
*G. barretti*	Depth (6.2)		Depth (5.5)	Slope (9.5)
	Min. Sal. (4.6)		Min. Sal. (4.0)	Min. Sum. Chl-*a* (3.3)
	Max. Fall Chl-*a* (3.1)			
*G. phlegraei*	Min. Sal. (6.7)		Depth (5.7)	Depth (11.7)
	Min. Cur. (3.9)		Min. Sal. (4.6)	Min. Sal. (6.7)
	Max. Cur. (2.9)			Max. Temp. (3.0)
*Geodia spp.*	Min. Sal. (4.6)		Depth (8.6)	Min. Sal. (10.2)
	Depth (3.1)		Min. Sal. (3.9)	Depth (8.6)
	Max. Cur. (2.8)			Max. Temp. (3.2)
Sponge grounds	Max. Cur. (6.9)	Min. Sal. (9.6)	Min. Sal. (10.0)	Max. Temp. (7.9)
	Depth (4.0)	Min. Cur. (5.7)	Depth (4.1)	Min. Fall Chl-*a* (2.7)
	Min. Sal. (2.6)	Depth (5.3)	Max. Temp. (2.1)	Depth (1.8)

Note: Abbreviations used for salinity (Sal.), chlorophyll-*a* (Chl-*a*), current (Cur.) and temperature (Temp.). All values except Chl-*a* refer to the seafloor.

The large number of models prevents all mapped predictions and their interpretation from being reported within space constraints. Mapped predictions are therefore presented only for sponge ground models as these have the most direct conservation value. Additional mapped predictions are presented when they provide pertinent illustration of other results.

**Figure 2:**
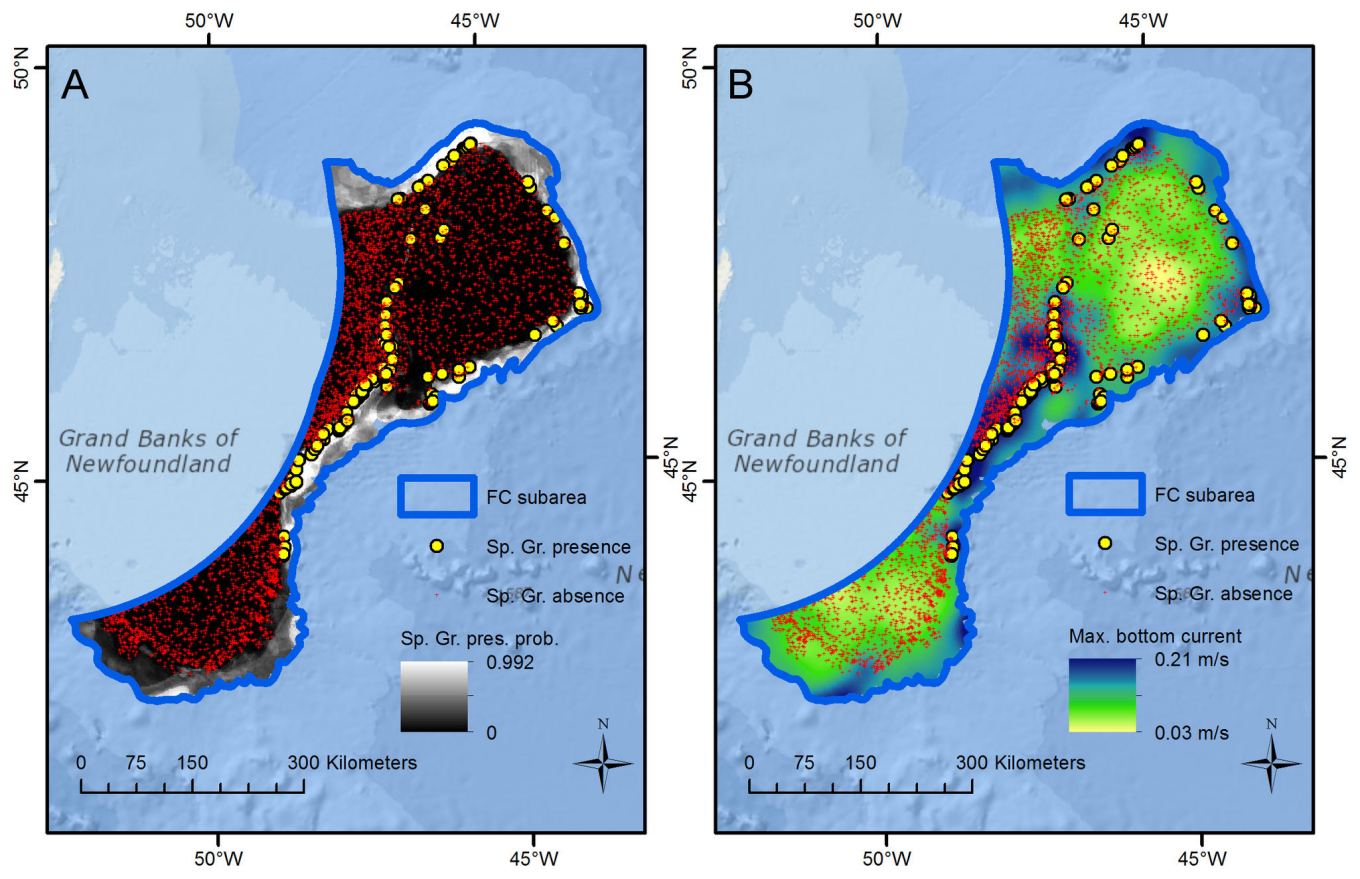
Spatial concordance between sponge grounds and maximum bottom current, FC subarea. The figure shows (A) sponge ground (Sp. Gr.) presence/absence observations and predictions of presence probability (pres. prob.), and (B) the distribution of maximum bottom current, for the FC subarea. Sponge grounds are primarily observed and predicted to exist in areas with high (>0.1 m/s) maximum bottom current.

For the FC subarea the sponge ground model fit was excellent (AUC = 0.982) and sponge ground presence is predicted with high probability on the upper continental slope where numerous observations confirm their presence, and with moderate probability in the Flemish Pass (Figure 2) where *Geodia* spp. are known to be present, but not abundant, and sponge fauna is dominated by the glass sponge *Asconema* sp. and demosponges of the family Axinellidae [5]. Due to the frequent presence observations with high depth values, predictions of high presence probability also extend into the deeper (unsampled) parts of the area, where validation will require additional field observations. Apart from these unsampled areas, the areas of high predicted presence probability align with the areas of significant concentrations of sponges derived from the kernel distributions derived from biomass data [11] which are currently closed to fishing with bottom-contact fishing gears [46] in response to the UNGA Resolution 61/105. In this region maximum bottom current was the strongest predictor variable; sponge grounds are predominantly found in areas of high (>0.1 m/s) maximum bottom current east of Grand Bank and around the Flemish Cap.

**Figure 3:**
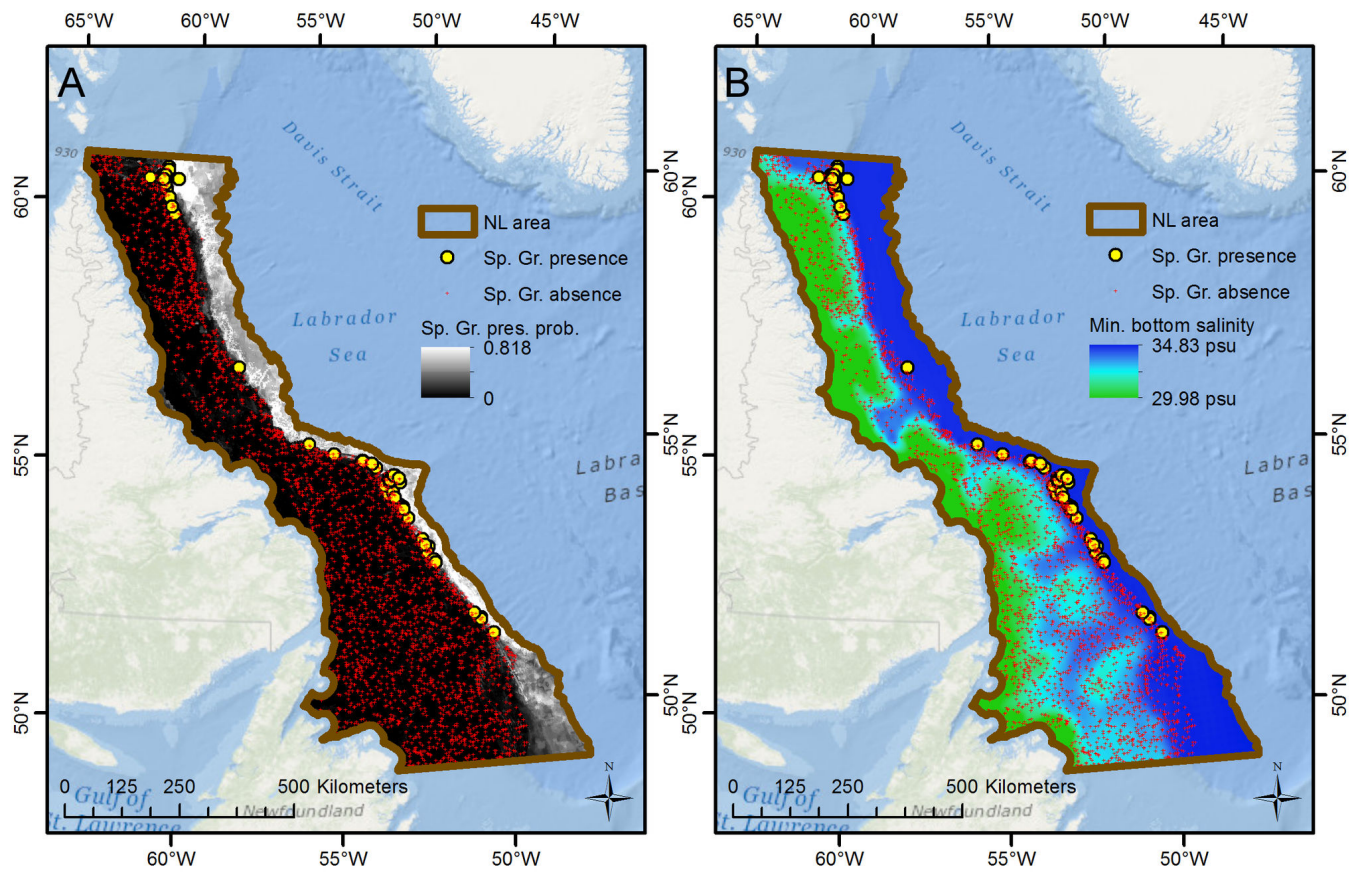
Spatial concordance between sponge grounds and minimum bottom salinity, NL subarea. The figure shows (A) sponge ground presence/absence observations and predictions of presence probability, and (B) the distribution of minimum bottom salinity, for the NL subarea. Sponge grounds are primarily observed and predicted to exist in areas with high (>34.3 psu) minimum bottom salinity.

For the NL subarea the sponge ground model fit was also excellent (AUC = 0.946), and sponge ground presence is predicted with high probability along the edge of the continental slope north of the Orphan Basin (Figure 3). Minimum salinity is an important predictor variable (Table 5) and sponge grounds are found in the more saline water along the continent slopes and absent from the fresher water on the shelf. Within the slope waters minimum current speed is also important (Table 5).

**Figure 4:**
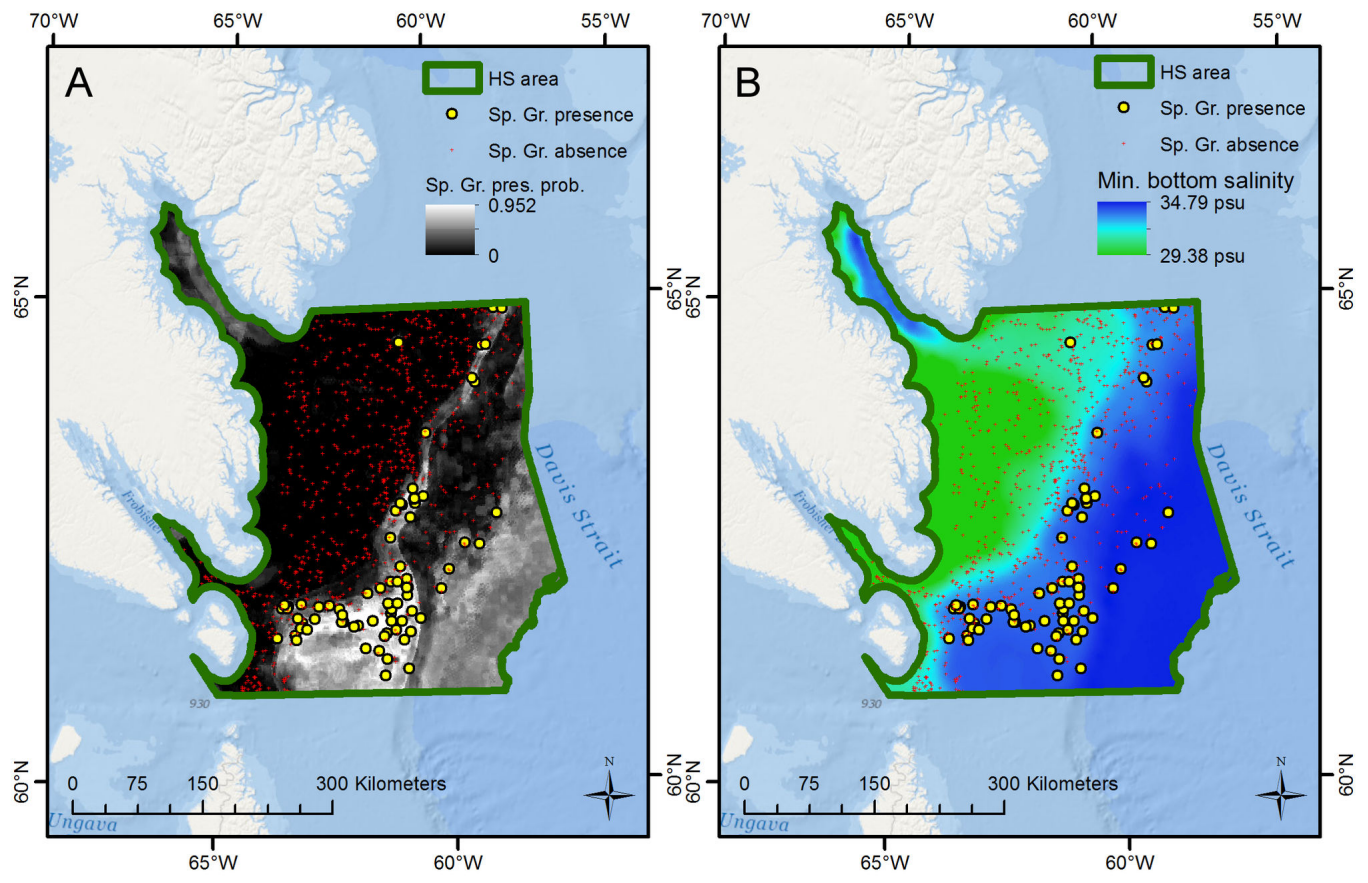
Spatial concordance between sponge grounds and minimum bottom salinity, HS subarea. The figure shows (A) sponge ground presence/absence observations and predictions of presence probability, and (B) the distribution of minimum bottom salinity, for the HS subarea. Sponge grounds are primarily observed and predicted to exist in areas with high (>34.4 psu) minimum bottom salinity.

For the HS subarea the sponge ground model fit was also excellent (AUC = 0.920) and known sponge ground locations in a narrow band on the continental shelf and a larger area on the periphery of Hatton Basin (a broad, roughly circular basin with water depths close to 600 m), east of Resolution Island, are all correctly predicted as having high presence probability, while moderate presence probability is predicted for the unsampled continental slope in the southeastern part of this area (Figure 4). The spatial data show this distribution to be at depths between 500 and 600 m (a narrow interval given the variability in the area) with minimum salinity above 34.4 psu, and with maximum bottom temperatures between 4 and 5°C. Minimum salinity was the most important predictor variable, with depth and maximum bottom temperature enhancing the performance. Sponge grounds in this area are currently not under direct conservation management although a segment of the fishing industry has introduced a voluntary closure in the south, east of Resolution Island [11,47]; the observations and predictions in our study could provide a solid basis for spatial fisheries management in this area.

**Figure 5:**
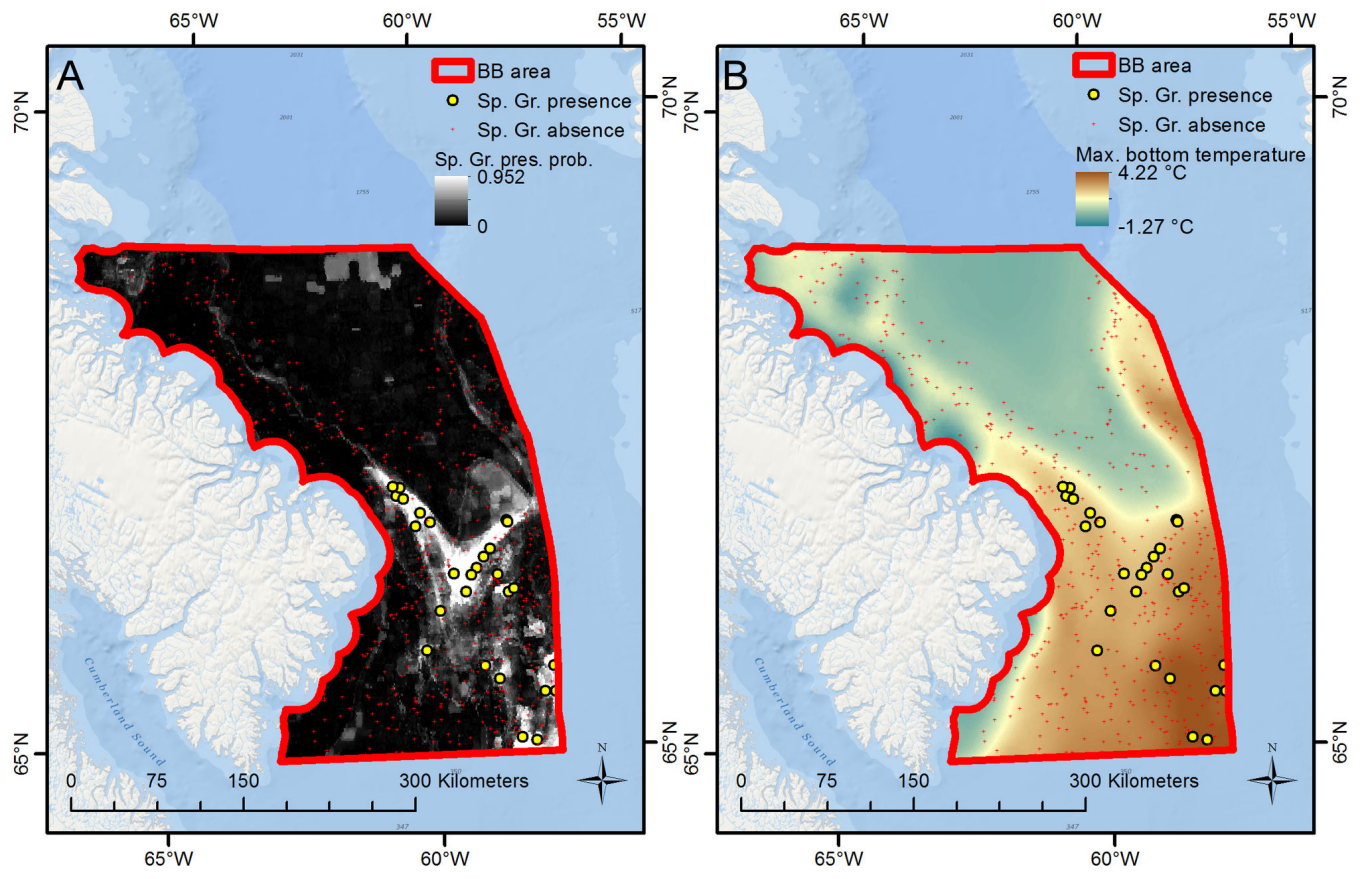
Spatial concordance between sponge grounds and maximum bottom temperature, BB subarea. The figure shows (A) sponge ground presence/absence observations and predictions of presence probability, and (B) the distribution of maximum bottom temperature, for the BB subarea. Sponge grounds are primarily observed and predicted to exist in areas with high (>2.2 °C) maximum bottom temperature.

The sponge ground model for the BB subarea had a lower fit (AUC = 0.795), but high concordance between the predicted surface and the known distribution of sponge grounds in the area is still present (Figure 5). The sponge grounds are constrained to a V-shaped area and maximum bottom temperature has the greatest influence on the predictions followed to a lesser degree by minimum fall chlorophyll levels at the surface, and water depth (Table 5). The strong association of the sponge grounds in this northern area with maximum bottom temperature is illustrated in Figure 5. Similar to their distribution in HS, these sponge grounds are also located on the edge of the shelf and in an area of relatively warm water, although the temperatures in BB are not as high as elsewhere. Maximum temperatures in the "V" (Figure 5) are 2.2-2.3°C, while they drop below 1°C in the deeper (Baffin Bay) area immediately to the north.

**Figure 6:**
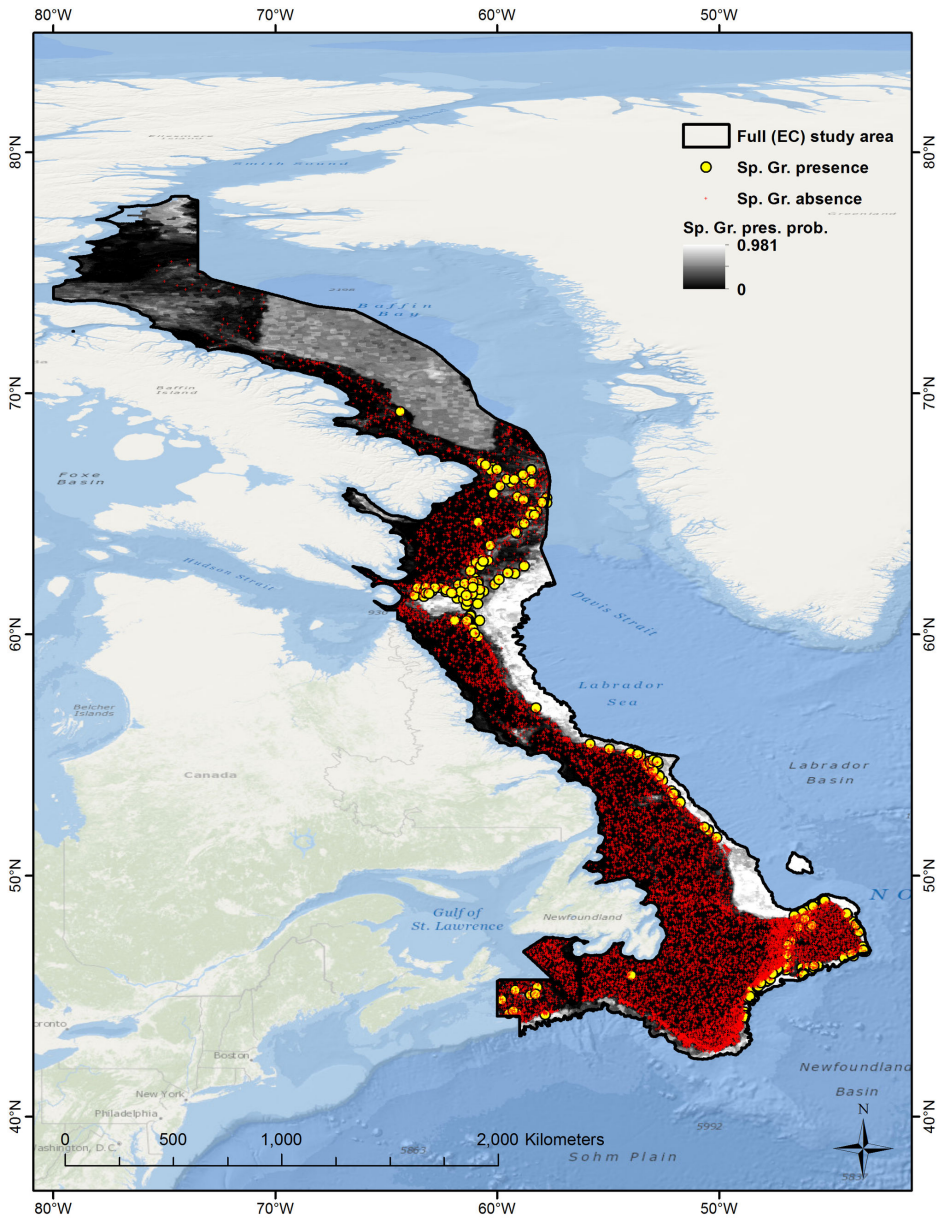
Sponge ground presence/absence observations and predictions of presence probability for the full (EC) study area.

For the full (EC) study area the sponge ground model fit was also excellent (AUC = 0.957) and better than that of some subareas (Table 4), although a comparison between observations and predictions (Figure 6) reveals that not all sponge grounds locations were predicted as having high presence probability, while large unsampled areas, specifically the deeper areas off the Labrador Slopes and in Baffin Bay, are predicted as having moderate/high presence probability. Uneven sampling density between the four subareas is likely to have biased the model towards higher fit in the FC subarea (higher sampling density) and lower fit in the BB subarea (lower sampling density). Very low presence probability is predicted for the known sponge grounds locations on the Scotian Shelf in the southwestern part of the study area; sponge grounds in this area are not dominated by Geodia spp.

**Figure 7:**
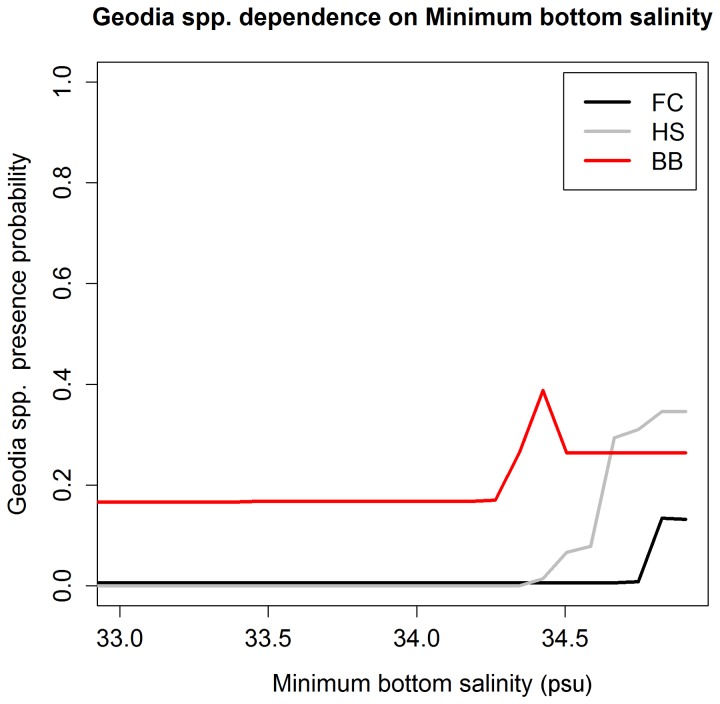
*Geodia* spp. presence probability dependence on minimum bottom salinity for three subareas. For each subarea (FC, HS and BB), the line shows predicted *Geodia* spp. presence probability when minimum bottom salinity is varied through the full range of observations while all other predictors are held constant at their mean observed value.

**Figure 8:**
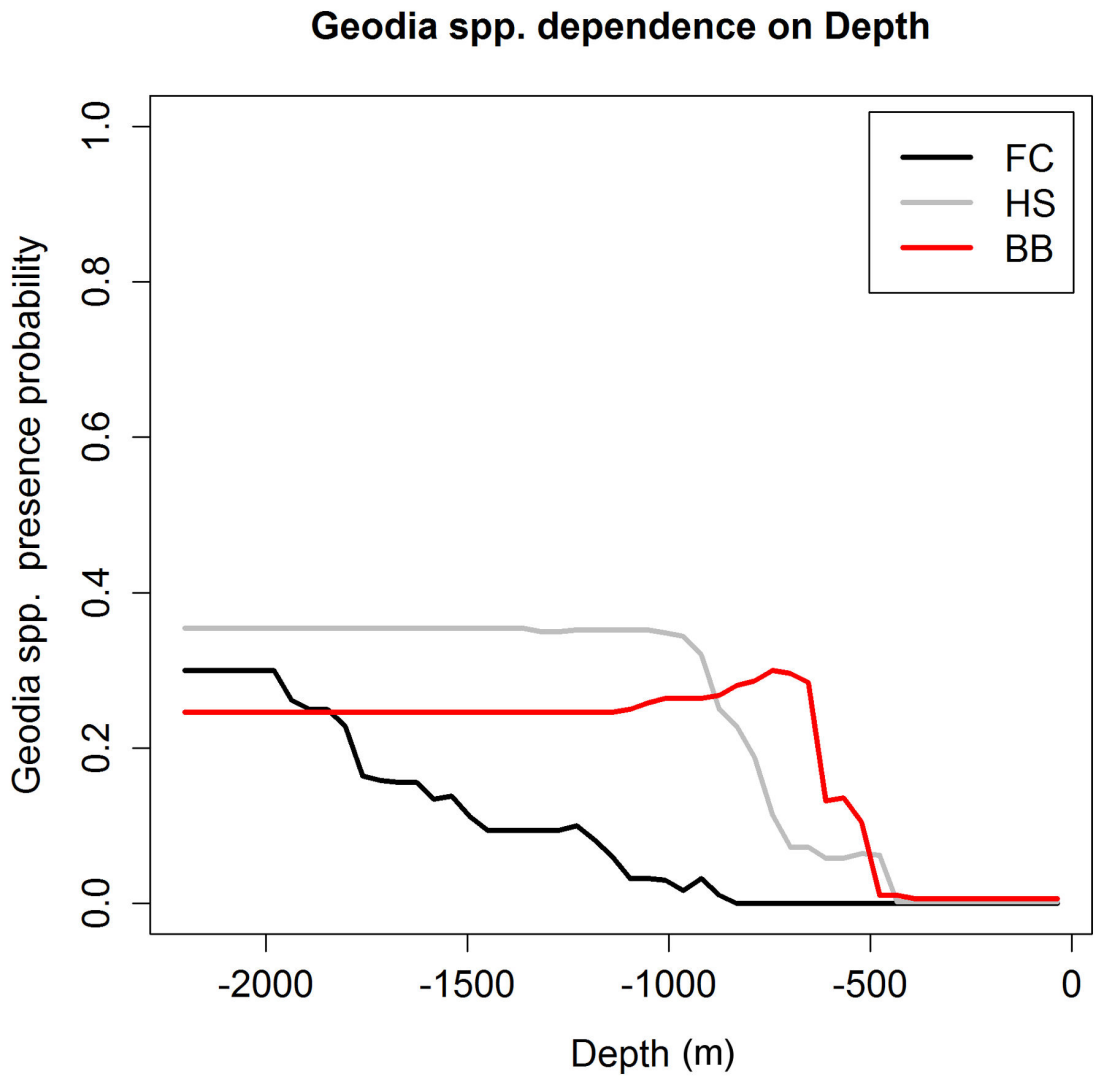
*Geodia* spp. presence probability dependence on depth for three subareas. For each subarea (FC, HS and BB), the line shows predicted *Geodia* spp. presence probability when depth is varied through the full range of observations while all other predictors are held constant at their mean observed value.

Detailed information on differences and similarities between model structures and the relationships between the response variables and their predictors is provided by the partial response plots. Figures 7 and 8 show the dependence of *Geodia* spp. presence on minimum bottom salinity and depth, which were identified as the two most important predictors of *Geodia* spp. in the FC, HS and BB subareas (Table 5). Although *Geodia* spp. presence probability generally increases with increasing values of minimum bottom salinity, the specific relationship, including presence probability at low salinity values and the value at which presence probability beings to increase, differs between the three subareas. Similarly, higher *Geodia* spp. presence probability is associated with deeper areas (0 indicates the sea surface), although *Geodia* sponges are found at much shallower locations in the HS and BB subareas than in the FC subarea. The slopes of the probability functions also differ between FC and the other subareas. In the north *Geodia* spp. show a sharp increase in presence probability with increasing depth, whereas in the FC subarea the increase is more gradual. The dependences of *G. barretti*, *G. phlegraei* and sponge grounds on minimum bottom salinity and depth, not shown here, all closely mirror those shown for *Geodia* spp.

### Model extrapolation

**Figure 9:**
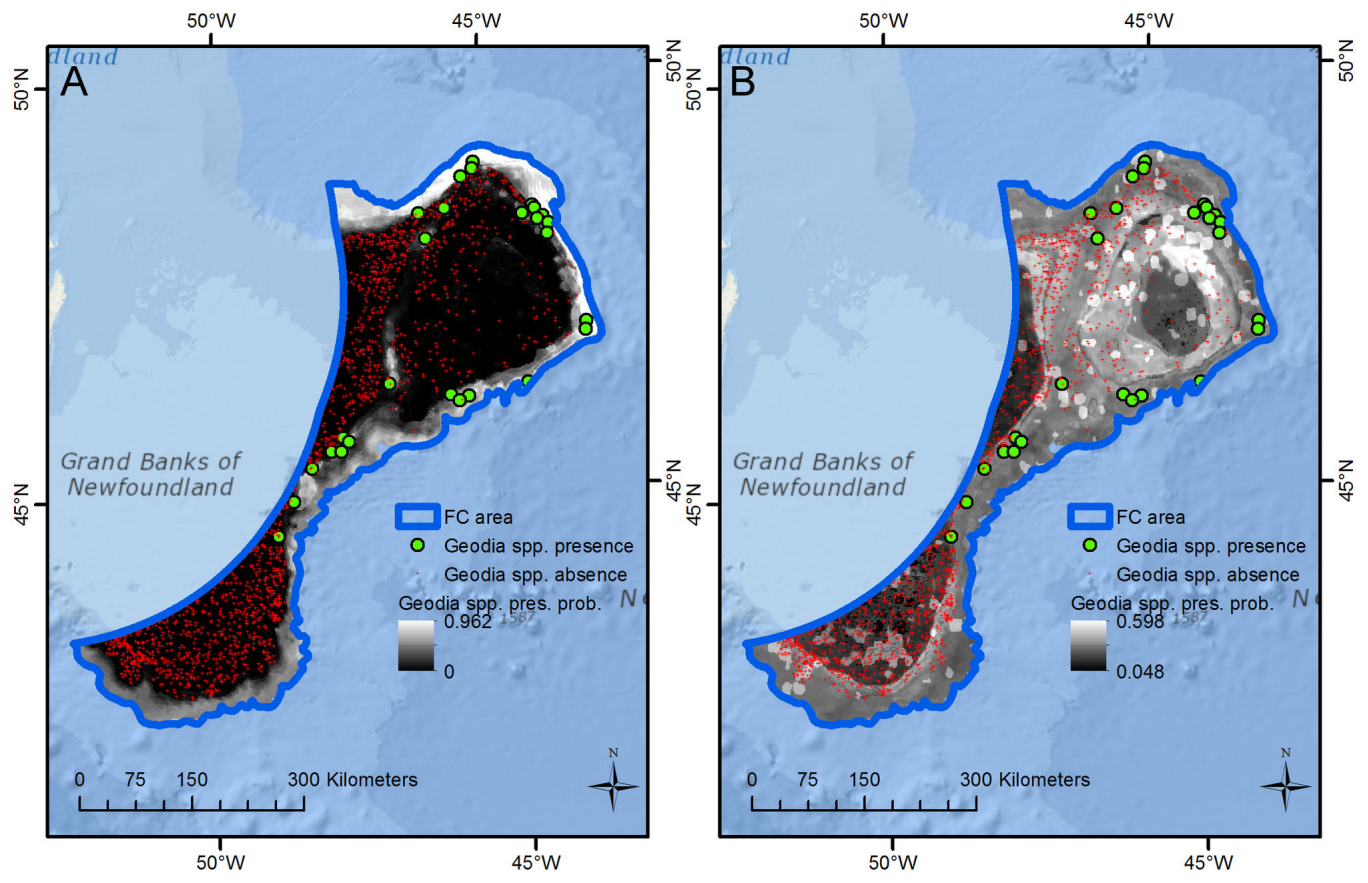
Observed and predicted distributions of *Geodia* spp. in the FC subarea. The two maps illustrate the difference between predictions made by (A) the model trained for this area (AUC = 0.939), and (B) the model trained for the BB subarea (AUC = 0.486).

An important use of SDM is identification of unsampled areas that are likely to contain presences, as such areas can be targeted in future surveys or precautionary management measures put in place until further data become available. However, in general fit is substantially reduced when models are trained in one area and extrapolated to another (Table 6). Some extrapolated models, such as the *Geodia* spp. model extrapolated from the BB to the FC subarea, are no better than random (AUC ≈ 0.5), despite very good fits when models were trained for the area of predictions. An example is shown in Figure 9, which compares predictions of *Geodia* spp. in the FC subarea between the model trained for this area (a) and the model trained for the BB subarea (b). Other extrapolated models retain high AUC values implying excellent model fits, such as the *G. phlegraei* model extrapolated from the BB to the HS subarea (AUC 0.962).

**Table 6:** AUC values quantifying model fits for models trained in one area and extrapolated to another.

**Subarea**	***Geodia barretti***	***Geodia phlegraei***	***Geodia* spp.**	**Sponge grounds**
FC	HS: 0.828	HS: 0.903	HS: 0.814	HS: 0.776
	BB: 0.738	BB: 0.741	BB: 0.486	BB: 0.606
				NL: 0.659
				FC: 0.735
NL				HS: 0.915
				BB: 0.733
	FC: 0.618	FC: 0.561	FC: 0.524	FC: 0.652
HS	BB: 0.675	BB: 0.962	BB: 0.939	BB: 0.855
				NL: 0.765
	FC: 0.651	FC: 0.623	FC: 0.663	FC: 0.565
BB	HS: 0.712	HS: 0.718	HS: 0.765	HS: 0.625
				NL: 0.574

Note: The area each model was trained in is indicated in each cell.

**Figure 10:**
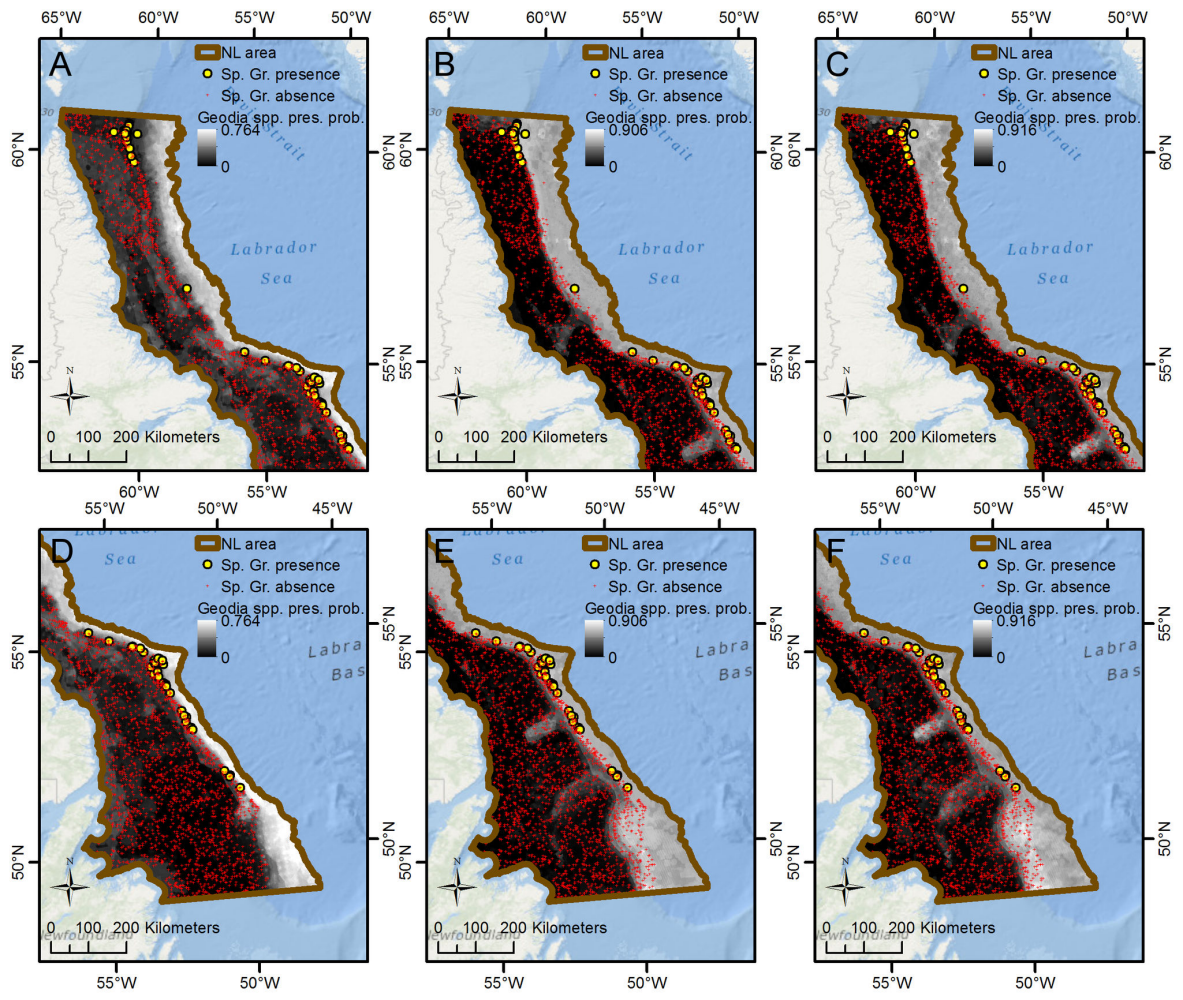
Observed sponge grounds and predictions of *Geodia* spp. presence probability by three models, NL subarea. The six maps illustrate the difference between extrapolations of three different *Geodia* spp. models to the northern (A-C) and southern (D-F) parts of the NL subarea. The three models were trained on data from (A, D) the FC subarea (AUC = 0.735), (B, E) the HS subarea (AUC = 0.915), and (C, F) the BB subarea (AUC = 0.733).

Similarly, Figure 10 shows extrapolations to the NL subarea of three *Geodia* spp. models trained on the FC, HS and BB subareas respectively. Although no genus-level identification in NL is available within our data set to validate the fits of these three models, an assumption of *Geodia* spp. presence at all NL subarea sponge grounds [19] can be used to provide a visual assessment of their performance. With the exception of the most northerly cluster of sponge grounds, the model trained on the HS subarea (Figures 10b and e) produces predictions that align well aligned with the observed sponge grounds, with low presence probability predicted on the shelf where no sponge grounds have been found and higher presence probabilities on the upper slope where sponge grounds are located. However, for Orphan Basin in the southeastern part of the NL subarea the model trained on the neighbouring FC subarea (Figure 10d) performs better than the model trained on the HS and BB subareas (Figures 10e and f) as it does not predict high presence probability in upper slope areas with numerous absence observations. Sampling in the deeper water of Orphan Basin where this model predicts a high probability of *Geodia* spp. presence could provide further insight into whether models trained on the FC subarea are actually better predictors for this subregion or not. The model trained in the northern BB subarea and extrapolated to the NL subarea (Figures 10c and f) generally shows the poorest concordance with the known distributions of the *Geodia* spp. and predicts more extensive habitat on the continental shelf than is known to occur.

## Discussion

### Data

Species distribution models are intimately linked to the sampling devices used to generate their training data, and combining large data sets from different gear types (e.g. trawls and underwater photos) can introduce bias and poor model performance. The research trawl data set predominantly used in this study is large, geographically extensive, and species identifications have been conducted by experts in the field. However, the data, and hence the models and their predictions, are in part a function of gear catchability, that is the proportion of the swept biomass that is recovered for weighing on deck. In general, the catchability of deep-sea sponges from any research trawl is unknown and likely to be low as many deep-sea sponges are small, fragile and may disintegrate before reaching the deck for weighing and identification, even if encountered by the trawl gear. The two *Geodia* species used in our study are exceptions: *G. barretti* is massive and can reach 80 cm in diameter and a wet weight of more than 35 kg, while *G. phlegraei* can reach more than 20 cm in diameter [9]. However, even if swept sponges of these two species are more likely to reach the deck for weighing and identification when encountered by the trawl gear, their patchy distribution on the seafloor can still allow them to go undetected when the trawl gear samples between sponge patches. This leads to imperfect (but unknown) detectability, likely to be higher in high-density areas (sponge grounds) than in lower-density areas. Data for the FC subarea suggests that catchability for both the Campelen and Lofoten trawl gear used to generate our data is on the order of 2%, although others report values of up to 70% for large sponges [48]. Knowledge of detectability has been shown to enable improved distribution modeling for a species, when methods that take it into account are employed [49]. In the absence of robust estimates of catchability, detectability of the *Geodia* species/genus was not accounted for in our modeling, and catchability was only indirectly taken into account through the use of local and gear-specific thresholds used to differentiate sponge grounds from lower-density sponge presence.

The environmental predictors used in our study by no means constitute an exhaustive list of factors that may influence the distribution of *Geodia* spp. sponges and sponge grounds in the study area, and other variables are available from online databases [50,51]. SDM studies of *Lophelia pertusa* and their reefs collectively include a wider range of topographic variables including aspect, curvature and a bathymetric position index [24,26], as well as data on alkalinity, aragonite saturation state and concentrations of dissolved inorganic carbon, dissolved oxygen, nitrate, phosphate and silicate [22,23,25], all derived with different accuracies and at different spatial resolutions. However, not all these predictors proved important to predict *Lophelia pertusa* distributions, and it is conceivable that some variables would have gained or lost importance for prediction had they been quantified differently and/or measured at a different spatial resolution. Selection of environmental predictors is a subjective exercise that depends on knowledge of the biology and ecology of the species in question, and is additionally constrained by the availability of spatially continuous data sets that accurately quantify potentially influential environmental variables with appropriate metrics applied at relevant spatial and temporal scales. The predictors used in our study were derived, within those constraints, from the limited knowledge of *Geodia* spp. ecology. Potentially important predictors that we did not have data for include substrate type, which may influence larval settlement, and historical trawling intensity. Further, short-term dynamics of the variables were not quantified but could be highly relevant. For example, *Geodia barretti* is known to respond negatively to rapid increases in temperature, and in the Skagerrak mass mortality has been reported following a seabed temperature increase of approximately 4°C above previous maxima in a 24 hour period [52]. If data were available such anomalies could be incorporated into SDM analyses, but the time frame over which we summarized the data and our use of ocean circulation models to extract environmental variables precludes their identification in our analyses.

### Model fit and interpretation

Only two models, predicting *G. barretti* and sponge grounds in the BB subarea, did not perform excellently (AUC >= 0.9). One reason for their poorer performance may be the variable elimination process, during which variables were removed based on their correlation in the full (EC) study area. The sill that separates the Labrador and Baffin Basins causes correlations between environmental variables to differ markedly between the areas north and south of appr. 65 °N, and the variable elimination may therefore have removed some predictors that were uncorrelated (|R| < 0.5) in the BB subarea. When models for the BB subarea were fit using all available predictors (not shown), AUC values increased substantially for both *G. barretti* (from 0.531 to 0.671) and sponge grounds (from 0.795 to 0.850). Another reason for the poorer performance of models in the BB subarea may be that the relatively few presence data records from this area are less likely to fully capture the environmental influences on sponge distribution.

The predicted distribution patterns showed no consistent differences between the two *Geodia* species, the *Geodia* genus, and the sponge ground habitat. This is not surprising given that these sponges are both highly aggregating and major constituents of the sponge grounds in the study area.

The extent to which the models have been able to identify a plausible lower tolerance limit of *Geodia* spp. for minimum bottom salinity is uncertain, but the different limits identified for the three areas (see Figure 7) suggest that either tolerance of low salinity water limit is genuinely different for *Geodia* spp. populations in the FC, HS and BB subareas, or the correspondence between the distributions of *Geodia* spp. and minimum bottom salinity is caused at least partly by one or more additional oceanographic variables. Genuinely different tolerance limits could be a result of local adaptation. Although this is highly speculative without further research, the possibility is supported by short-range larval dispersal in *Geodia* spp. and corresponding low connectivity between the FC and HS/BB subareas, which are >1500 km apart. Sponge larvae are uniformly non-feeding and short-lived (except for rare known exceptions), generally staying only a few hours in the water column [53] and settling in the vicinity of parental populations [54]. With such high levels of larval retention [55] it is possible that connectivity among patches is very low and that the patches are highly inbred and potentially locally adapted. As a minimum, the existence of a lower salinity tolerance limit for *Geodia* spp., located in the 34.3-34.8 psu range (Figure 7), is a hypothesis that warrants future investigation. Unfortunately very little is known about the physiological tolerances of these sponges. Tolerance limits for depth make for less appealing hypotheses given the proxy function of this predictor, and the larger difference between the apparent upper (shallow) limits in the two areas. Lower (deep) limits are difficult to assess in both study areas due to poor sampling of deep areas; no such limit appears to have been identified given that eight of the ten deepest sampled locations show *Geodia* spp. presence, including the six deepest (depths 1827-2201 m). The differences in the presence probability functions associated with depth seen between the FC and the northern HS and BB subareas could be explained by differences in slope. The Flemish Cap has steep slopes to the south and southeast and gentler slopes to the west and north. These could produce the gradual change in probability observed there (Figure 8). Alternatively, fishing in the area could have eroded the natural distributions at the shallow end of the distribution as the fisheries are primarily in the north and west of the Flemish Cap [56]. 

In the future, a greater geographic and depth range of samples may be used to provide a better basis for identification of real physiological tolerance limits. Sufficient coverage in environmental space, i.e. sampling beyond both the lower and upper tolerance limits of a given environmental variable, should produce partial dependence plots that reach presence probabilities near zero at both extremes. As seen in figures 7 and 8 our data do not include samples beyond the upper minimum bottom salinity tolerance limit or the lower (deeper) depth tolerance limit for *Geodia* spp.

### Extrapolation

Although extrapolation produced reduced fits in all cases, the ability to extrapolate predictions to unsampled areas is an important strength of SDM in comparison to other approaches used to map sponge grounds [11]. In the extrapolation of the *Geodia* spp. model from the HS to the NL subarea (Figure 10b), high (>0.5) presence probability is predicted for the outer continental shelf and the continental slope immediately beyond the currently sampled area, where minimum bottom salinity exceeds 34.60 psu and depth exceeds 500 m. No samples from depths >1800 m exist to validate the predictions here, and this area could be targeted during future research trawls to locate new *Geodia* spp. presences and confirm our assumptions of their association with the sponge grounds. When extrapolation is based on the *Geodia* spp. model from the FC subarea (Figure 10a), high predicted presence probability is generally restricted to areas with minimum bottom salinity exceeding 34.78 psu and depth exceeding 1800 m, which are optimal conditions in the FC subarea but not in the NL subarea.

The better performance of extrapolations from the HS subarea to the NL subarea, compared to those from the FC subarea to the NL subarea, may be explained by the common influence of the deep Labrador Current on the HS and NL subareas forming a coherent temperature-salinity environment [57-59]. This deep water current carries water of Arctic origin from Baffin Bay, West Greenland and, to a lesser degree, Hudson Bay along the edges of the Labrador and Newfoundland shelves to the northeastern slope of the Grand Banks (FC subarea) where it bifurcates. The main branch moves southward through Flemish Pass along the shelf edge of Grand Bank (known as the shelf-edge branch) at speeds of 0.3 m/sec [60] where it interacts with bottom topography (canyons) causing local upwelling [61]. A lesser branch moves eastward along the northern slope of Flemish Cap. The 3000-m isobath is often considered as the offshore limit of the deep Labrador Current [62]. In the FC subarea this current pushes Labrador slope water up and over Flemish Cap where circulation is dominated by an anticyclonic gyre [60]. The northward flowing North Atlantic Current (NAC) transports warmer (> 4°C), high salinity water to the northeast along the southeast slope of the Grand Banks and the Cap. The NAC is comprised of waters from the Slope Water Current and from the Gulf Stream. The close proximity of the NAC to the Flemish Cap creates an interaction with the two current water masses (Labrador Current and NAC), resulting in warmer waters and elevated nutrients on the Flemish Cap compared to the Grand Banks Shelf [63]. Thus the majority of the FC subarea is subjected to different physical oceanographic conditions from those of HS and NL subareas, which may explain the better performance of the model in predicting sponge grounds in the NL subarea when trained with data from the HS, rather than the FC, subarea.

The southwest slopes of Orphan Basin in the NL subarea do not support sponge grounds or *Geodia* spp. This discontinuity in the species distribution could be explained by prehistoric events which may have shaped the present day distribution. This area was subjected to a widespread large submarine landslide about 7000 years ago along the southwestern edge of Orphan Basin, but seemingly absent further east on Sackville Spur [64]. This event would likely have destroyed the sponges and changed the surficial geology and topography of the area which is characterized by weak slopes. 

The relatively poor ability for models trained in the BB subarea to predict sponge distributions in the other areas and vice versa can also be explained by its different oceanography. Baffin Bay is a deep body of water located between Greenland and Baffin Island. Water depths are generally less than 1000 m, but reach a maximum 2400 m near its centre. The bay is connected to the Arctic Ocean in the north through Nares Strait, Jones and Lancaster Sounds, and to the Labrador Sea in the south by Davis Strait, which forms a sill at about 640 m depth. More than 80% of the bay is covered by ice in the winter, which changes the temperature and salinity structure in the upper water layers by melting and freezing. The temperature and salinity of this area includes a cold fresh surface layer that penetrates to about 200 m (Rudels 1986) and likely explains the absences of the sponges from the continental shelves. Labrador Sea Water brought north by the West Greenland Current produces a temperature and salinity maximum at about 500 m (> 0.5°C, 34.5 psu) although most of the water in this layer and in the deeper bottom water arrives from the Arctic through Nares Strait [65]. Deep water is likely formed as a result of mixing of Arctic and Atlantic waters. These bottom waters are colder (

**Figure 11:**
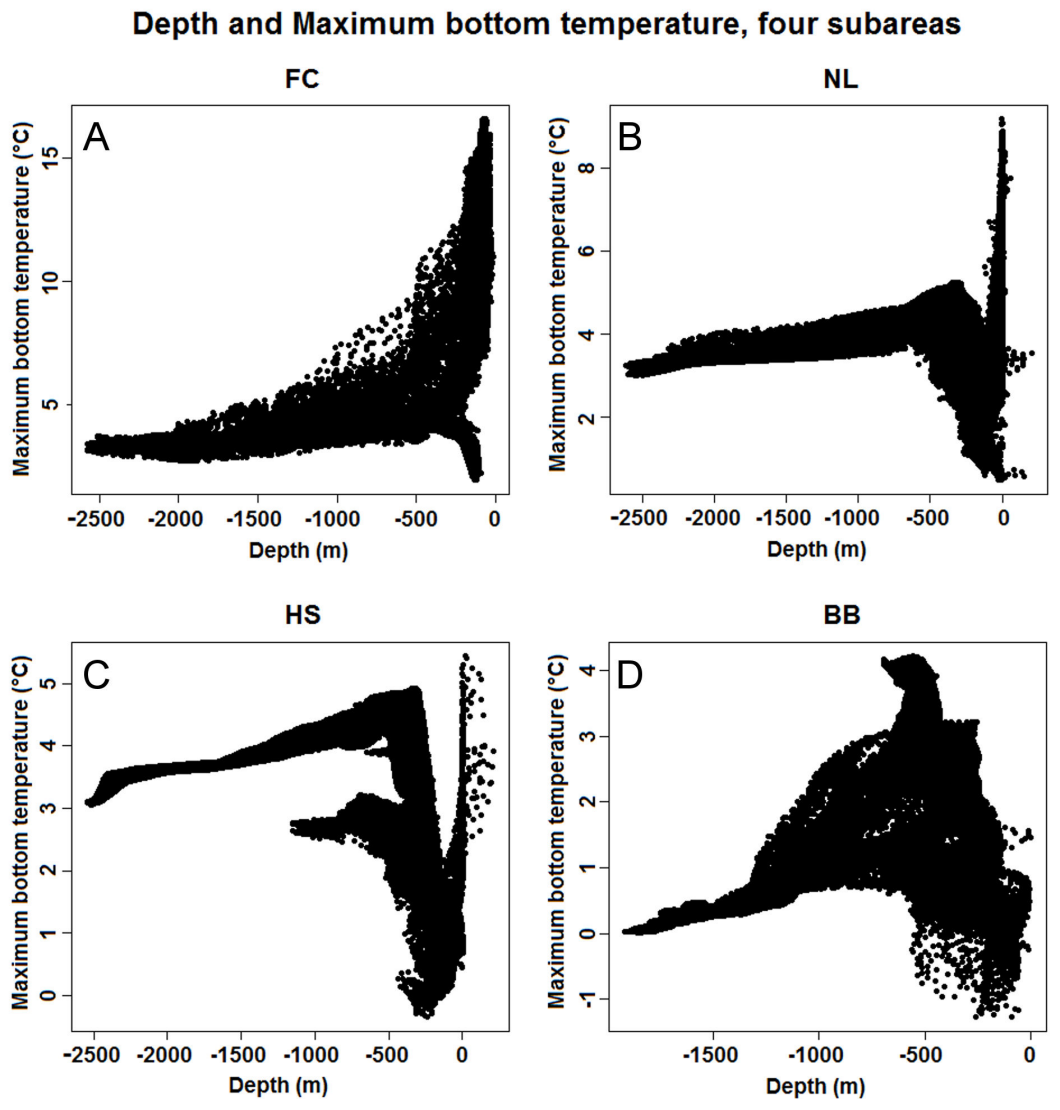
Varying relationships between depth and maximum bottom temperature. The relationship between depth (a proxy predictor) and maximum bottom temperature (a potentially causal predictor) differs dramatically between the four subareas, probably causing depth to be a poor predictor of distributions when used in models extrapolated between areas. Note the different scale of the y-axes on each figure.

<-0.4°C) and fresher (34.25 -34.5 psu) than the temperature maximum layer and water exchange in the deep water is relatively small, creating depleted oxygen and high nutrient conditions [66]. A specific problem associated with differences in physical oceanography for the different subareas relates to the use of proxy predictors that do not quantify environmental variables for which physiological tolerance limits are likely to exist, but rather correlate with one or more such variables. Depth, for example, is most likely to influence Geodia spp. distributions when acting as a proxy correlated with physical and chemical environmental factors such as nutrient availability, temperature, salinity and currents. Interpretation of relationships between Geodia spp. distributions and such proxy predictors is not ecologically meaningful, and although they may help models produce accurate predictions for the area they were trained for they may also reduce the accuracy of such models when extrapolated to new areas with different oceanographic contexts. For example, the extrapolation of the Geodia spp. from the FC to the HS subarea (AUC = 0.524) is substantially improved when the depth and slope variables are removed from the training data (AUC = 0.771) because this allows other (non-proxy) variables greater influence on predictions. The problem with extrapolation based on depth (or other proxy variables) is that different relationships exist between depth and the factors it correlates with in different geographic areas. For example, Figure 11 illustrates the relationships between depth and maximum bottom temperature (which potentially has causal influence on Geodia spp. distributions) in the four subareas. Although all subareas show different and complex relationships between these two variables, the known differences in oceanography mean that the existence of relatively warm water (>10 °C) in shallow parts of the FC subarea (A) is not matched in the other areas, and t

hat the near-linear relationship between depth and maximum bottom temperature for parts of the FC, NL (B) and HS (C) subareas, from approximately 3°C at 2500 m depth to approximately 5 °C at 500 m depth, is not found in the BB subarea (D), which is colder (0-1°C) below approximately 1500 m.

Despite such problems, extrapolation results currently constitute the only predictions of sponge grounds in unsampled sections of the study area and may help indicate areas, such as the deeper parts of Baffin Bay and the Newfoundland and Labrador slopes (Figure 6), where sponge grounds are most likely to be found in the future.

## Conclusion

The excellent fit of most models strongly supports the use of SDM for local mapping of both *Geodia* spp. sponges and sponge grounds, suggesting that these models are suitable for outlining sponge ground areas to be considered for VME designation. To our knowledge this is the first application of SDM for mapping distributions of deep-sea sponges, and although *Geodia* sponges are among the largest and most durable of deep-sea sponges and thus particularly amenable to sampling with research trawls, the results also suggest that SDM may be an effective tool for mapping distributions of other deep-sea sponges with appropriate sampling. Genus-level models generally performed as well as species-level models. Model fits were especially high in small and well-sampled regions, while extrapolations between areas with different oceanographic regimes performed poorly. Depth and minimum bottom salinity were generally the two most important predictors of the distribution of *Geodia* spp. sponges and sponge grounds, and a *Geodia* spp. minimum bottom salinity tolerance threshold in the 34.3-34.8 psu range was hypothesized on the basis of model structure. Maximum bottom temperature, minimum bottom current speed, slope and seasonal chlorophyll-*a* concentration at the sea surface also contributed to the models. Although extrapolated models produced reduced fits, extrapolations within oceanographic regimes constitute the best predictions of sponge grounds in unsampled sections of the NWA. These models indicated two areas, the deeper parts of Baffin Bay and the Newfoundland and Labrador slopes, where sponge grounds are most likely to be found in the future.

## Supporting Information

Technical Report S1 **DFO Technical Report containing an earlier iteration of this study.** The report has additional partial dependence plots and variable importance results, but relies on different approach for variable elimination and a merged version of the BB and HS subareas.
(PDF)
