# Supplementary material for: Modeling the Distribution of Geodia Sponges and Sponge Grounds in the Northwest Atlantic
Source: PLoS One. 2013 Dec 4;8(12):e82306. doi: 10.1371/journal.pone.0082306 (PMC3852940; doi:10.1371/journal.pone.0082306)
Supplement: Technical Report S1 — DFO Technical Report containing an earlier iteration of this study. The report has additional partial dependence plots and variable importance results, but relies on different approach for variable elimination and a merged version of the BB and HS subareas. (PDF) [file pone.0082306.s001.pdf]

Distribution Modeling for Sponges and Sponge Grounds in the Northwest Atlantic Ocean

A. Knudby, E. Kenchington, A.T. Cogswell, C.G. Lirette, F.J. Murillo

Science Branch, Maritimes Region
Ocean and Ecosystem Sciences Division
Fisheries and Oceans Canada
1 Challenger Drive
Dartmouth, NS
B2Y 4A2

2013

Canadian Technical Report of Fisheries and Aquatic Sciences 3055

Fisheries and Oceans Canada Pêches et Océans Canada

Canada

Canadian Technical Report of Fisheries and Aquatic Sciences

Technical reports contain scientific and technical information that contributes to existing knowledge but which is not normally appropriate for primary literature. Technical reports are directed primarily toward a worldwide audience and have an international distribution. No restriction is placed on subject matter and the series reflects the broad interests and policies of Fisheries and Oceans Canada, namely, fisheries and aquatic sciences.

Technical reports may be cited as full publications. The correct citation appears above the abstract of each report. Each report is abstracted in the data base *Aquatic Sciences and Fisheries Abstracts*.

Technical reports are produced regionally but are numbered nationally. Requests for individual reports will be filled by the issuing establishment listed on the front cover and title page.

Numbers 1-456 in this series were issued as Technical Reports of the Fisheries Research Board of Canada. Numbers 457-714 were issued as Department of the Environment, Fisheries and Marine Service, Research and Development Directorate Technical Reports. Numbers 715-924 were issued as Department of Fisheries and Environment, Fisheries and Marine Service Technical Reports. The current series name was changed with report number 925.

Rapport technique canadien des sciences halieutiques et aquatiques

Les rapports techniques contiennent des renseignements scientifiques et techniques qui constituent une contribution aux connaissances actuelles, mais qui ne sont pas normalement appropriés pour la publication dans un journal scientifique. Les rapports techniques sont destinés essentiellement à un public international et ils sont distribués à cet échelon. II n'y a aucune restriction quant au sujet; de fait, la série reflète la vaste gamme des intérêts et des politiques de Pêches et Océans Canada, c'est-à-dire les sciences halieutiques et aquatiques.

Les rapports techniques peuvent être cités comme des publications à part entière. Le titre exact figure au-dessus du résumé de chaque rapport. Les rapports techniques sont résumés dans la base de données *Résumés des sciences aquatiques et halieutiques.*

Les rapports techniques sont produits à l'échelon régional, mais numérotés à l'échelon national. Les demandes de rapports seront satisfaites par l'établissement auteur dont le nom figure sur la couverture et la page du titre.

Les numéros 1 à 456 de cette série ont été publiés à titre de Rapports techniques de l'Office des recherches sur les pêcheries du Canada. Les numéros 457 à 714 sont parus à titre de Rapports techniques de la Direction générale de la recherche et du développement, Service des pêches et de la mer, ministère de l'Environnement. Les numéros 715 à 924 ont été publiés à titre de Rapports techniques du Service des pêches et de la mer, ministère des Pêches et de l'Environnement. Le nom actuel de la série a été établi lors de la parution du numéro 925.

Canadian Technical Report of
Fisheries and Aquatic Sciences 3055

2013

Distribution Modeling for Sponges and Sponge Grounds in the Northwest Atlantic Ocean

A. Knudby[1], E. Kenchington, A.T. Cogswell, C.G. Lirette, F.J. Murillo[2]

Science Branch
Ocean and Ecosystem Sciences Division
Fisheries and Oceans Canada
1 Challenger Drive
Dartmouth, NS
B2Y 4A2

[1] Department of Geography, Robert C. Brown Hall, Simon Fraser University, Burnaby, BC V5A 1S6
[2] Instituto Español de Oceanografía, Centro Oceanográfico de Vigo, Programa de Pesquerías Lejanas, Apartado 1552, 36280 Vigo, Spain

© Her Majesty the Queen in Right of Canada, 2013.
Cat. No. Fs 97-6/2855E ISSN 0706-6457

Correct citation for this publication:

Knudby, A., E. Kenchington, A.T. Cogswell, C.G. Lirette, F.J. Murillo. 2013. Distribution modeling for sponges and sponge grounds in the northwest Atlantic Ocean. Can. Tech. Rep. Fish. Aquat. Sci. 3055: v + 73 p.

TABLE OF CONTENTS

ABSTRACT .... **V**

RÉSUMÉ .... **VI**

INTRODUCTION **1**

SPONGE GROUNDS 1
MAPPING SPONGE GROUNDS 2
Habitat Suitability Modeling *2*
OBJECTIVES OF STUDY 3

STUDY AREA **4**

DATA AND METHODS **6**

FIELD DATA 6
Sponge Ground Locations *6*
Geodia spp. *Locations* *7*
ENVIRONMENTAL DATA LAYERS 12
Depth and Slope *12*
Chlorophyll-a Concentration *12*
Physical Ocean Variables *13*
DISTRIBUTION MODELING 14
Model Types *14*
Variable Elimination *14*
Removal of Highly Correlated Variables 14
Removal of Variables That Did Not Contribute to Model Performance 15
Model Evaluation *18*
Model Fit for Training Area 18
Model Extrapolation 19
Partial Dependence Plots 19
Variable Importance 19

RESULTS AND DISCUSSION **20**

VARIABLE ELIMINATION 20
MODEL EVALUATION 22
Model Fit for Training Area *22*
Model Extrapolation *23*
Partial Dependence Plots *29*
Variable Importance *32*
GLOBAL VS. LOCAL GEAR-SPECIFIC THRESHOLDS FOR DEFINING SPONGE GROUNDS 33
SPECIES- VS. GENUS-LEVEL RESPONSE VARIABLES 37
SDM-BASED DISTRIBUTION MAPS 39

CONCLUSION **42**

REFERENCES **43**

APPENDIX **49**

PARTIAL DEPENDENCE PLOTS 49
FC Study Area *49*
Geodia spp. 49
G. barretti *51*
G. phlegraei *53*
Sponge Grounds 55

EC Study Area *56*
Sponge Grounds 56
G. barretti *58*
G. phlegraei *60*
Sponge Grounds (Local Gear-Specific Thresholds) 62
Sponge Grounds (Global Thresholds) 64
VARIABLE IMPORTANCE RESULTS 66
FC Study Area *66*
Geodia spp. 66
Geodia barretti *66*
Geodia phlegraei *66*
Sponge Grounds 67
MAPPED PREDICTIONS, FC STUDY AREA 68
Geodia spp. *68*
G. barretti *69*
G. phlegraei *70*
Sponge Grounds *71*
ACKNOWLEDGEMENTS 73

ABSTRACT

Knudby, A., E. Kenchington, A.T. Cogswell, C.G. Lirette, F.J. Murillo. 2013. Distribution modeling for sponges and sponge grounds in the northwest Atlantic Ocean. Can. Tech. Rep. Fish. Aquat. Sci. 3055: v + 73 p.

Species distribution models (SDM), trained using research trawl data and spatial information on depth, slope, chlorophyll-*a* concentration, shear, surface and bottom temperature, salinity and current speed, were used to make predictions of the distributions of *Geodia* spp. sponges and sponge grounds in the northwest Atlantic Ocean. We compared two model types often used for SDM, random forest (RF), a machine-learning method based on presence/absence data, and MaxEnt (ME), a machine-learning method based on presence-only data. This comparison included the fits obtained on independent data from the area each model was trained for as well as the fits obtained on independent data from a different area (extrapolation), the spatial predictions each model produced, and the ability of each model to produce sensible partial dependence plots and realistic measures of the importance of each predictor variable for model predictions. Of the two model types tested, RF generally out-performs ME when tested against independent validation data; the difference between the two model types is larger when models trained for the Flemish Cap are extrapolated to the full East Coast study area. Comparing the RF model with the kernel analyses previously employed, there is a general distribution match between the maps. However the RF model predictions have a more refined distribution as would be expected given its use of absence data in addition to the presence data. For management and conservation purposes, RF can be used to refine kernel models of the location of sponge grounds and to predict sponge ground areas where data are either sparse or non-existent.

RÉSUMÉ

Knudby, A., Kenchington, E., Cogswell, A.T., Lirette, C.G., Murillo, F.J. 2013. Modélisation de la répartition des éponges et des lits d'éponges dans le nord-ouest de l'Atlantique. Can. Tech. Rep. Fish. Aquat. Sci. 3055: v + 73 p.

Les modèles de répartition des espèces, formés à partir de données de recherche de chalutage et d'information spatiale sur la profondeur, la pente, les concentrations de chlorophylle-*a*, le cisaillement, la température à la surface et de fond, la salinité et la vitesse actuelle, ont été utilisés pour faire des prédictions quant à la répartition des éponges *Geodia* spp. et des lits d'éponges du nord-ouest de l'Atlantique. Nous avons comparé deux types de modèles souvent utilisés pour le modèle de répartition des espèces, soit le modèle de forêts aléatoires (RF), une méthode d'apprentissage automatique basée sur les données relatives à la présence et à l'absence, et le modèle MaxEnt (ME), une méthode d'apprentissage automatique basée sur les données relatives à la présence uniquement. Cette comparaison incluait les ajustements obtenus à partir de données indépendantes de la zone pour laquelle chaque modèle a été formé de même que les ajustements obtenus à partir de données indépendantes d'une zone différente (extrapolation), les prédictions spatiales produites par chaque modèle ainsi que la capacité de chaque modèle à produire des graphiques de dépendance partiels sensibles et des mesures réalistes de l'importance de chaque variable prédictive pour les prédictions de modèle. Parmi les deux types de modèle essayés, la performance du modèle RF dépasse largement d'habitude celle du ME lorsque des données de validation indépendantes sont utilisées dans l'évaluation; la différence entre les deux types de modèle est plus importante lorsque des modèles formés pour le Bonnet Flamand sont extrapolés à la pleine zone d'étude de la côte est. En comparant le modèle RF avec les analyses de noyaux utilisées antérieurement, on note une correspondance dans la répartition générale des cartes. Cependant, les prédictions du modèle RF présentent une répartition plus précise comme on pourrait s'y attendre compte tenu de l'utilisation des données d'absence et de présence. Aux fins de gestion et de conservation, le modèle RF peut être utilisé pour améliorer les modèles de noyaux de l'emplacement des lits d'éponges et pour faire des prédictions sur les zones de lits d'éponges lorsqu'il n'y a pas de données ou que les données sont rares.

INTRODUCTION

Sponges form an ancient group of sessile filter-feeders that occur as sparsely distributed solitary organisms or in dense single- or multi-species aggregations. They are characterized by a body plan built around a system of canals through which water is pumped, supplying food and oxygen and removing waste (Hooper and van Soest 2002).

Sponges are highly effective filter feeders and play a clear role in benthic-pelagic coupling (ICES 2009). They also enhance local nutrient and energy exchange (de Goeij and van Duyl 2007) and biodiversity (Klitgaard 1995; Hentschel *et al.* 2002, Beazley *et al.* 2013). They constitute an important component of benthic ecosystems, especially when they occur in dense aggregations known as sponge grounds or "ostur", which provide a structurally complex habitat for fish and invertebrates (Herrnkind *et al.* 1997; Amsler *et al.* 2009; Kenchington *et al.* 2013; Beazley *et al.* 2013). The foundation for these sponge grounds are often the large, erect, aggregating sponges. In the North Atlantic, these are species of *Geodia, Stryphnus, Stelletta, Thenea, Tetilla* and *Polymastia* (ICES 2009).

Due to the inaccessibility of their habitats relatively little is known about deep-sea sponges, and most research has focused on the anatomy and taxonomy of trawl-caught specimens (Hooper and van Soest 2002; Cárdenas *et al.* 2013), biology and associated fauna (Klitgaard 1995; van Soest *et al.* 2013), broad-scale geographic distribution (Klitgaard and Tendal 2004; Kenchington *et al.* 2010; Murillo *et al.* 2012*)*, and the potential of their secondary metabolites for drug development (Hill 2004; Müller *et al.* 2004).

The threat posed by bottom-contact fisheries has sparked conservation concern, especially in light of deep-sea sponges being slow-growing and long-lived organisms vulnerable to disturbance (Klitgaard 1995, Hogg *et al.* 2010). The combination of their vulnerability and importance for deep-sea benthic ecosystems makes sponge grounds a prime candidate for Vulnerable Marine Ecosystem (VME) designation as outlined in United Nations General Assembly Resolution 61/105 (UNGA 2007), and hence subject to conservation action (Penney *et al.* 2009). Because deep-sea conservation is largely carried out through spatial fisheries management, this has generated a need to map the distributions of sponges and sponge grounds at the level of spatial detail needed for such management.

Sponge Grounds

The distributions of individual sponge species and genera can be described or predicted through presence/absence or presence-probability maps (Franklin 2009), as can the distribution of sponge grounds following a definition of what constitutes a sponge ground. Due to the general lack of data on sponge density and the spatial extent of sponge aggregations, published definitions are mostly qualitative in nature, including "a restricted area where large-sized sponges are strikingly common" (Klitgaard and Tendal

2004), and "aggregations of large sponges that develop under certain geological, hydrological and biological conditions to form structural habitat" (Hogg *et al.* 2010). Klitgaard *et al*. (1997) defined 'ostur' as areas where 90% of the wet weight of trawl catches is comprised of sponges, excluding fish. One quantitative approach to distinguishing the presence of sponge grounds from a background area with some sponge presence at lower density was developed by Kenchington *et al*. (2009), who used a kernel density approach to delineate areas covered by research trawl catches above a range of weight thresholds, and used an abrupt change in the area covered by catches above two neighbouring threshold values to indicate the difference between sponge grounds and lower density sponge presence for a local area. The application of this approach to individual trawl gear types and bioregions (DFO 2009) in the northwest Atlantic Ocean (Kenchington *et al.* 2010), is adopted in this study.

Mapping Sponge Grounds

Knowledge of the spatial distribution of deep-sea sponges and sponge grounds is currently limited to compilations of presence observations derived from a variety of sources, including research trawls (Fuller 2011; Murillo *et al.* 2012), the local knowledge of fishers (Klitgaard and Tendal 2004), or direct observations using SCUBA, remotely operated vehicles (ROVs) or crewed submersibles (Leys *et al.* 2004). Although such data compilations occasionally provide dense sampling coverage of an area (Kenchington *et al.* 2010), they are more often necessarily limited in both spatial extent and sampling density.

Sponge grounds in the northwest Atlantic are found along the continental slopes of Grand Bank and Flemish Cap and northward along the Labrador Slope (ICES 2009; Kenchington *et al.* 2009; Kenchington *et al.* 2010; Fuller 2011; Murillo *et al.* 2012). Murillo *et al.* (2012) described four areas with large aggregations of sponges in the high seas east of Newfoundland, Canada: the continental slopes of Grand Bank; the southeastern slope of the Flemish Cap; the eastern slope of the Flemish Cap; and the northern slope of the Flemish Cap and the Flemish Pass. There the Demosponges *Geodia barretti*, *G. phlegraei*, *G. macandrewii* (Geodiidae), *Stryphnus ponderosus* and *Stelletta normani* (Ancorinidae) are the main structure-forming sponges constituting more than 99 % of the total invertebrate biomass within the sponge grounds (Murillo *et al.* 2012).

Habitat Suitability Modeling

In the context of typically sparse point observations and a desire for complete and continuous maps of the distribution of a species or habitat type, species distribution modeling (SDM) is often used. We adopt the SDM term (Franklin 2009) for any model that predicts the presence, absence or abundance of a phenomenon (henceforth: the response), typically a species or habitat type, from environmental variables thought to influence it (henceforth: the predictors), when this model is applied to maps of the predictors to create spatially explicit and continuous predictions of the distribution of the

response. SDM has been extensively used in both terrestrial and marine environments to make contemporary distribution maps, to predict species/habitat responses to climate change (Knudby *et al.* 2010; Lawler *et al.* 2009), to predict the future range of invasive species (Peterson and Robins 2003; Peterson 2003) and more. SDM, despite its utility in data sparse situations, has rarely been used in the deep-sea. Existing studies include several focusing on the cold-water coral *Lophelia pertusa*, whose local, regional or global distributions are modeled on the basis of statistical relationships with variables that quantify a range of aspects related to seafloor topography as well as physical and chemical oceanography (Dolan *et al.* 2008; Davies *et al.* 2008; Guinan *et al.* 2009; Tittensor *et al.* 2009; Davies and Guinotte 2011; Yesson *et al.* 2012). The distribution of *Lophelia* reefs, as opposed to the presence of individual corals, has also been modeled on the basis of topographic variables derived from multi-beam data (Ross and Howell 2012). However, despite the recognized importance of sponges and sponge grounds as deep-sea benthic habitats, their distributions are still only known from point observations and SDMs have not to our knowledge been applied to sponges in the deep-sea.

Objectives of Study

This study explores the use of SDM-based mapping of sponges and sponge grounds in the northwest Atlantic (NWA) Ocean. Specifically,

1) We estimate the fits achieved by SDM models on independent test data from the study area, and compare results before and after application of two different approaches to elimination of collinear predictor variables;

2) We compare two model types often used for SDM, random forest (RF), a machine-learning method based on presence/absence data, and MaxEnt (ME), a machine-learning method based on presence-only data. This comparison includes the fits obtained on independent data from the area each model was trained for as well as the fits obtained on independent data from a different area (extrapolation), the spatial predictions each model produces, and the ability of each model to produce sensible partial dependence plots and realistic measures of the importance of each predictor variable for model predictions;

3) Sponge grounds, as distinct from a low density background presence of sponges, are defined using a threshold based on the wet sponge weight caught per tow. We compare two approaches to thresholding, a single global threshold and a variable threshold defined by gear type and bioregion (Kenchington *et al.* 2010), to assess the success of each approach for SDM-based mapping of sponge grounds;

4) We compare the accuracy of SDM models used to map the presence of individual members of the *Geodia* genus (*Geodia* spp.) against models used to map the two most prevalent species (*Geodia barretti* and *Geodia phlegraei*);

5) We produce SDM-based maps of sponges and sponge grounds that constitute the best available information on their distribution in the study area.

STUDY AREA

Data for this study come from the Canadian continental shelf and slope in the NWA, from the area off Cape Breton in the south to southern Ellesmere Island in the north (henceforth East Coast, abbreviated EC). This area is bounded in the north by NAFO zone 0A and in the south by NAFO zones 4Vs and 3Ps. The area is further bounded at a distance of 20 km from the Canadian coast in the west, and by the 2500 m depth contour or the eastern boundary of NAFO zones 0A and 0B, whichever is shallower, in the east. The area does not extend into Lancaster Sound or the Hudson Strait beyond the limits of NAFO zone 0A and NAFO zones 0B/2G respectively. In addition a smaller area, densely covered by field data, was defined around the Flemish Cap and Grand Bank (henceforth Flemish Cap, abbreviated FC). This area is bounded by the Canadian EEZ in the west and by the 2500 m depth contour at the continental slope, and forms a subsection of the EC area. The extents of both areas are shown in Figure 1 with the NAFO divisions in the area.

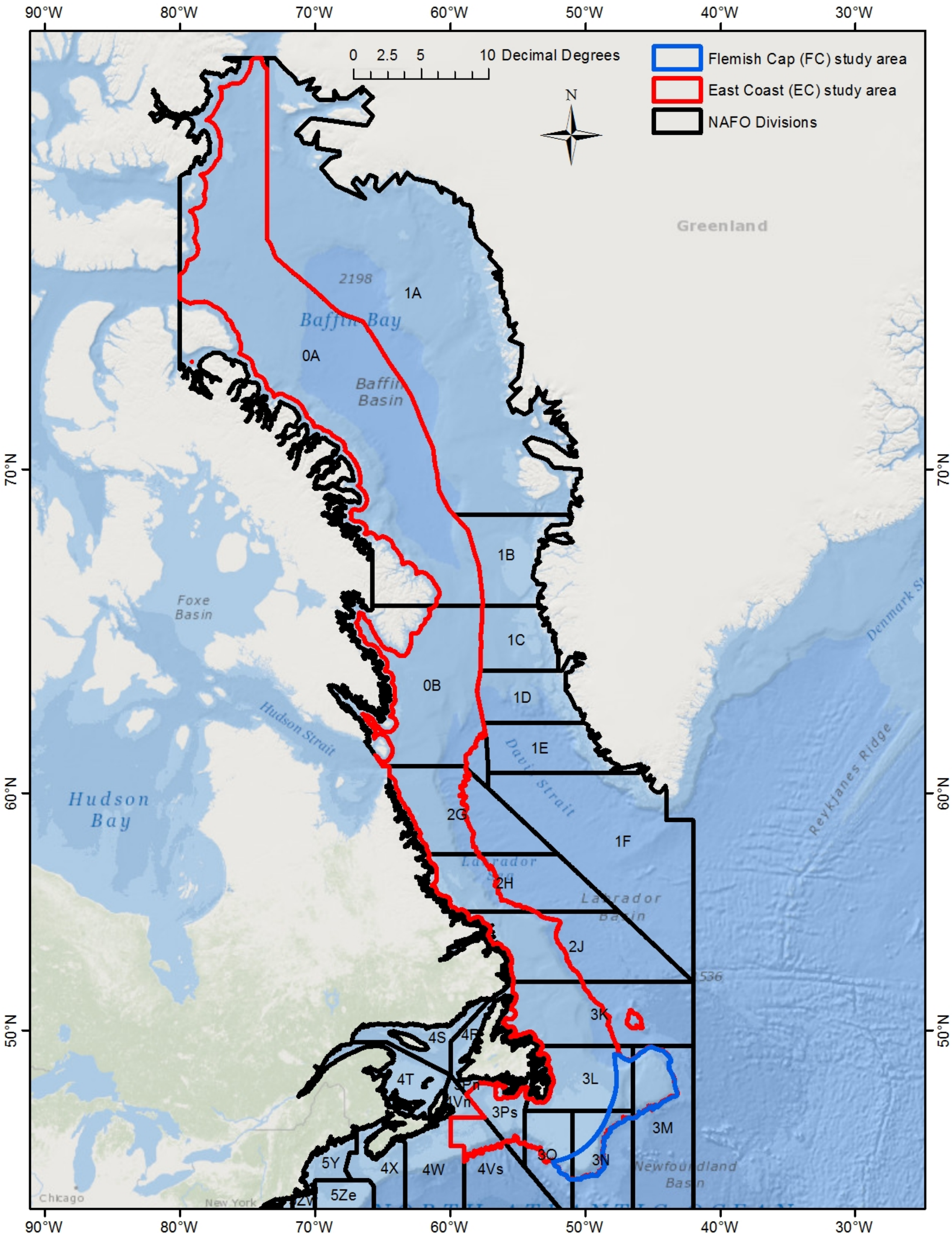

Figure 1. The East Coast (red) and Flemish Cap (blue) study areas, and NAFO divisions in the area (black).

DATA AND METHODS

The data used in this study form two major components, biological distribution data and environmental data. The biological data are derived from georeferenced samples collected from Canadian and European Union-Spanish (EU-Spain) research trawl surveys, via box-cores/dredges, or *in situ* camera/video. The environmental data are derived from satellite data and oceanographic model outputs.

Field Data

Sponge Ground Locations

The data used to identify locations of sponge grounds were collected via research vessel trawl surveys by Fisheries and Oceans Canada (DFO) and the Spanish Institute of Oceanography (IEO) in collaboration with other European institutions during the period of 2007-2011. Research trawl catches were, as a minimum, separated and weighed by phylum, to yield a measure of the total wet weight of caught sponges and quantified as kg/standard tow. In order to separate sponge grounds from non-sponge ground locations, a threshold was applied to this wet weight and locations with catches above the threshold were classified as a sponge ground presence, while those with catches below the threshold were classified as a sponge ground absence. However, different gear and trawl durations were used by the different missions, leading to differences in the area covered by each research trawl (swept area) and differences in the proportion of the swept benthic fauna that was recovered and available for weighing on deck (catchability). In order to use the entire data set in our study, separate thresholds were developed for each gear type and biogeographic region using kernel density analyses (Kenchington *et al.* 2009; Kenchington *et al.* 2010), and applied to the respective data points; these thresholds are summarized in Table 1. A global sponge grounds presence/absence threshold of 70 kg sponge/tow was also used as a comparison to the local gear-specific thresholds.

Table 1. Local gear-specific thresholds used to define sponge grounds.

Bioregion	Gear type	Local threshold (kg sponge/tow)
Eastern Arctic	Cosmos<br>Alfredo III<br>Campelen 1800	40<br>70<br>40
Newfoundland Labrador Shelves	Campelen 1800	200
Scotian Shelf	Western IIA	2
Flemish Cap	Campelen 1800	70 (DFO data)
Flemish Cap	Campelen 1800 and Lofoten	75 (DFO and EU-Spanish data)

Geodia* spp. *Locations

The catch from each research trawl was identified to the level of phylum, genus or species, depending on the taxonomic expertise available. Sponges can be very difficult to identify without examining the spicules microscopically and so most data were only recorded as the phylum Porifera. For the FC study area, species-level identification was carried out for some of the dredge/box-core collected during the Spanish led NAFO PotEntial VulneRable Marine Ecosystems-Impacts of Deep-sea Fisheries (NEREIDA) missions aboard the R/V Miguel Oliver (2009/10), which provided 20 data points with confirmed *Geodia* spp. presence records comprising multiple specimens from a single trawl set location. An additional 19 records were collected during EU-Spanish fisheries surveys in 2007 and identified by one of us (JM) and two additional *Geodia* spp. presence locations were obtained in 2010 during a DFO led NEREIDA mission aboard the CCGS Hudson using the ROV ROPOS (Canadian Scientific Submersible Facility, North Saanich, B.C.), for a total of 41 presence locations with species-level identification in the FC study area. Seven different *Geodia* species were identified: *G. barretti*, *G. phlegraei*, *G. hentscheli*, *G. atlantica*, *G. nodastrella*, *G. parva* and *G. macandrewii,* following the most recent taxonomy (Cárdenas *et al.* 2013). However *G. phlegraei* and *G. parva* are very closely related and we could not distinguish them with confidence in our data. As *G. parva* was only recently resurrected as a valid species from a subspecies of *G. phlegraei* we refer to our *G. parva* specimens as *G. phlegraei* hereafter. Of these, only *G. barretti* and *G. phlegraei* were recorded in sufficient numbers to allow species-level distribution modeling. *Geodia* spp. absences were compiled from DFO and IEO fisheries survey data. These surveys do not identify sponges beyond phylum level, so only catches with no sponges of any kind were used as *Geodia* spp. absences (Table 2). As noted by Kenchington *et al.* (2012), no sponges in a catch does not necessarily mean that there are no sponges on the sea floor, although any sponge presence is likely to be of low density.

Table 2. Origins of the FC study area data on *Geodia* spp. presence/absence.

Species	Sample Origin	Year	Gear Type	Number of Records
Geodia barretti presence	EU-Spanish Surveys	2007	Campelen 1800	6
			Lofoten	9
	NEREIDA (CCGS Hudson 029 - ROPOS)	2010	Remotely Operated Vehicle	1
Geodia phlegraei presence	EU-Spanish Surveys	2007	Campelen 1800	3
			Lofoten	10
	NEREIDA (R/V Miguel Oliver)	2009	Dredge	6
	NEREIDA (CCGS Hudson 029 - ROPOS)	2010	Remotely Operated Vehicle	1
Geodia spp. absence	EU-Spanish/DFO surveys	2007-2011	Various trawl gear	1632

Data for the EC study area include those listed above for the FC study, as well as additional data collected by DFO and detailed in Kenchington et al. (2010). Data on *Geodia* spp. absences from the EC study area were added from all DFO survey locations where no sponge of any kind was caught (Table 3). Occasionally the DFO Arctic surveys identified the sponge catch to genus level; when *Geodia* spp. sponges were identified these are listed in Table 3 as "*Geodia* spp. presence", *G. barretti* and/or *G. phlegraei* may or may not have been present at these locations.

Table 3. Origins of the EC study area data on *Geodia* spp. presence/absence.

Species	Sample Origin	Year	Gear Type	Number of Records
Geodia barretti presence	EU- Spanish surveys	2007	Campelen 1800	6
			Lofoten	9
	NEREIDA (CCGS Hudson 029 - ROPOS)	2010	Remotely Operated Vehicle	1
	DFO Fisheries Surveys (Arctic)	2010	Alfredo	1
			Cosmos	1
		2011	Alfredo	23
Geodia phlegraei presence	EU-Spanish surveys	2007	Campelen 1800	3
			Lofoten	10
	NEREIDA (RV Miguel Oliver)	2009	Dredge	6
	NEREIDA (CCGS Hudson	2010	Remotely Operated	1

	029 - ROPOS)		Vehicle	
	DFO Fisheries Surveys (Arctic)	2010	Alfredo	11
			Cosmos	6
		2011	Alfredo	15
Geodia spp. presence	DFO Fisheries Surveys (Arctic)	2010	Alfredo	2
			Cosmos	2
		2011	Alfredo	3
Geodia spp. absence	EU-Spanish/DFO Surveys	2007-2010	Various trawl gear	6819

Note that the data in Table 2 and Table 3 are treated as point observations, while in reality the research trawls covered an area as determined by the gear type and the direction and duration of the trawl. The geographic coordinate associated with each trawl was obtained at trawl start; for simplicity this location is used in the analysis to represent the entire trawl.

Based on the data outlined above we derived the presence/absence data sets listed in Table 4. It is clear from Table 4 that all data sets are unbalanced, with presences being relatively rare compared to absences. This may present problems for accurately mapping the rarer of the two values (Japkowicz and Stephen 2002; van Hulse and Khoshgoftaar 2009), in our case the presences. It is also clear that the two sponge species have a small number of presences, particularly for the FC study area, which may also be problematic in the model development (Stockwell and Peterson 2002).

Table 4. List of response variables with numbers of presence/absence values by study area.

**Response**	**Presences / Absences East Coast (EC)**	**Presences / Absences Flemish Cap (FC)**
Sponge ground (local gear-specific threshold)	322 / 11133	150 / 3455
Sponge ground (global threshold)	364 / 11091	150 / 3455
Geodia spp.	81 / 6819	41 / 1632
Geodia barretti	41 / 6819	16 / 1632
Geodia phlegraei	52 / 6819	20 / 1632

The distribution of these data points are illustrated in Figure 2 and Figure 3. A spatial bias towards dense sampling in the southern part of the EC study area is clear, with especially dense sampling (not clearly seen in the figure) in the FC study area.

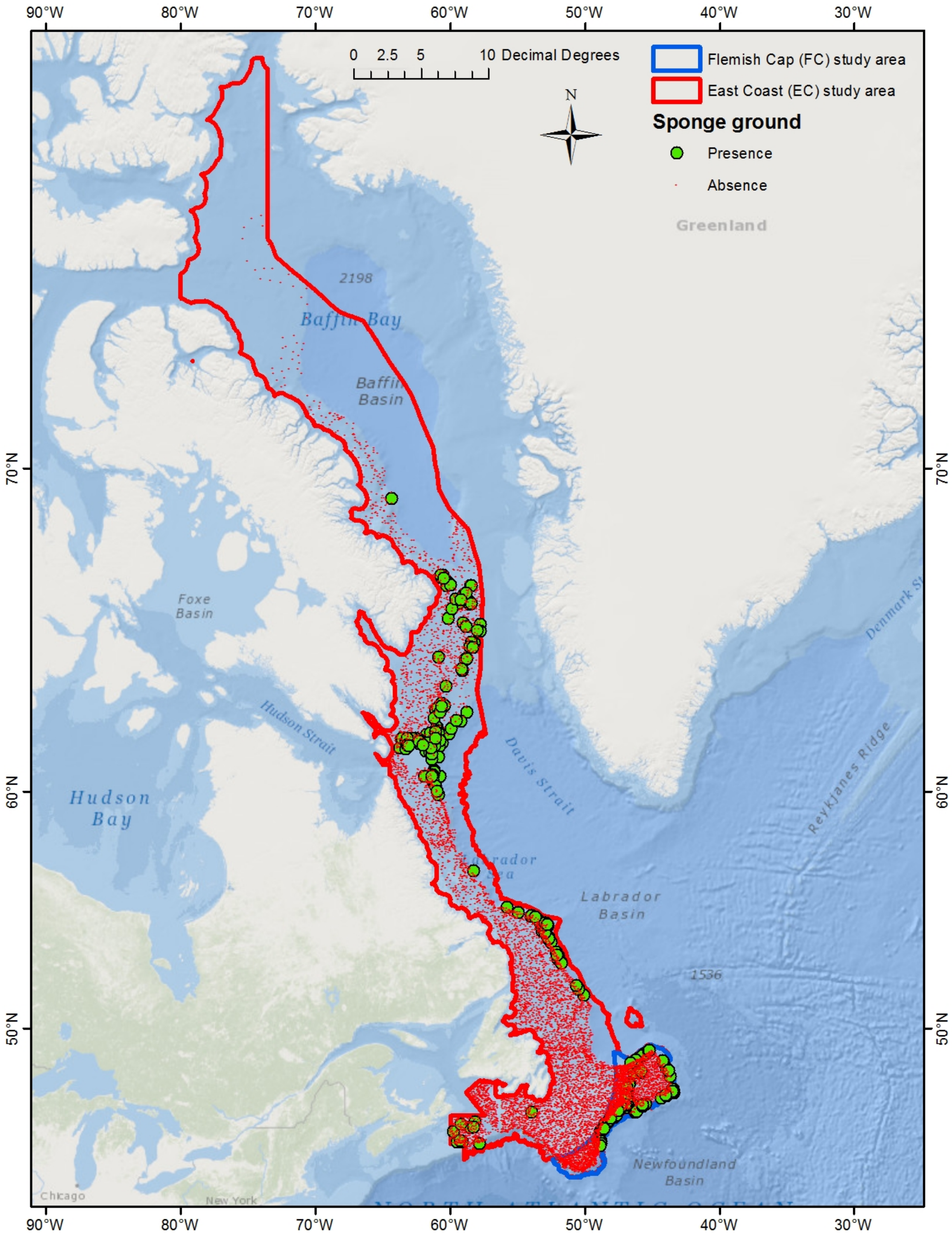

Figure 2. Spatial distribution of sponge ground presences as defined using local gear-specific thresholds (green circles) and absences (red crosses) as known from the field data.

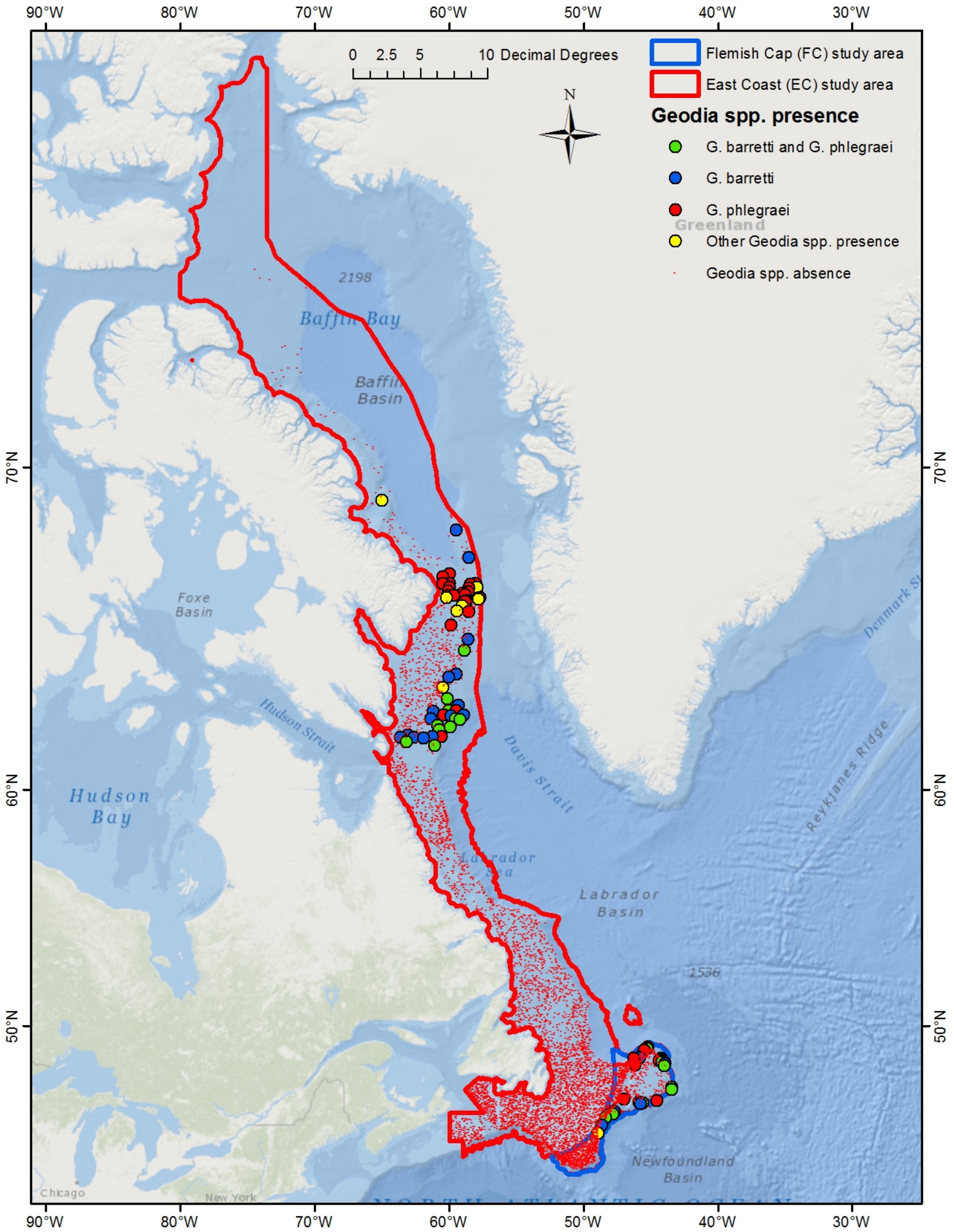

Figure 3. Spatial distribution of presences and absences for G. phlegraei (red), G. barretti (blue), both species (green), other *Geodia* species (yellow), and *Geodia* spp. absence (red cross).

Environmental Data Layers

While the point observations take the form of georeferenced presence/absence observations, the predictors in SDM take the form of spatial data sets (maps) that describe the spatially continuous distribution of factors thought to influence the spatial distribution of the response variable(s), at the appropriate spatial resolution (Franklin 2009). Given the limited knowledge of the habitat requirements and ecology of the sponge species in question, and the environmental conditions that may enable or limit the formation of sponge grounds, environmental data layers were selected on the basis of general notions of relevance and, crucially, availability. Data on a total of 50 predictors were compiled from different sources (Table 5), and transformed as necessary to geographic (lat/long) coordinates using the WGS 1984 datum and a 0.017° cell size; this cell size is approximately equal to a 1 km cell size in the southern part of the EC study area. The predictor data for the Flemish Cap area form a subset of the data for the East Coast area.

Depth and Slope

Bathymetric data were derived from the 30 arc-second General Bathymetric Chart of the Oceans (GEBCO) data (BODC 2009). Slope was derived in degrees from each depth layer using ArcGIS's Spatial Analyst tool. Depth is expected to act as a general proxy for unmeasured predictors including light availability and historical trawling intensity (negative correlation), in addition to being correlated with variables such as temperature and salinity ranges. Slope is expected to act primarily as a proxy for substrate type (e.g. rock, sand, silt, mud), which is thought to influence the ability of sponge larvae or fragments to settle in an area.

Chlorophyll-a Concentration

Data on surface chlorophyll-a concentration were derived from publicly available Level 3 SeaWiFS data for the period January 2001 – December 2010. These data are spatially composited to a 9 km cell size, and provided as monthly and annual mean values (NASA 2012). Annual minimum, maximum, mean and range statistics were calculated for each cell from the 10 annual data layers. Similarly, winter minimum, maximum, mean and range statistics were calculated from the December, January and February monthly data layers from each of the 10 years. This procedure was repeated to calculate seasonal statistics for spring, summer and fall. Kriging was performed using ArcGIS's Geostatistical Analyst to transform the 9 km cell size of the original data layers to the 0.017° cell size. Surface chlorophyll-a concentration is expected to be related to the export flux of particulate organic carbon (Lutz *et al.* 2007) and thus nutrient availability at the sea floor. Seasonal rather than annual measures of nutrient availability may be relevant due to the sponges' reproductive cycles, shown for *Geodia barretti* in the northeast Atlantic (Spetland *et al.* 2007).

Physical Ocean Variables

Data on shear stress at the seafloor, as well as temperature, salinity and current for the sea surface and seafloor were derived from the GLORYS2V1 ocean reanalysis at ¼ ° resolution. GLORYS2V1 provides 3-hourly estimates of these and other variables from 1993 to 2009 (Ferry *et al.* 2012). Minimum, maximum, mean and range statistics were calculated for each cell from these 3-hourly values. Kriging was subsequently performed using ArcGIS's Geostatistical Analyst to transform the ¼° cell size of the original data to the 0.017° cell size. Temperature and salinity can be used to identify water masses while all three may indicate physical tolerance limits of the sponges.

Table 5. Data layers used to quantify aspects of bathymetry, surface chlorophyll concentration, temperature, salinity and current at the sea surface and sea floor, and shear near the sea floor.

**All variables**	**Unit**	**Quantifications**	**Data source**	**Native resolution**
Depth	m	N/A	GEBCO	30”
Slope	degrees	N/A	GEBCO	30”
Annual chlorophyll	mg m^{-3}	Range, Min, Mean, Max	OceanColor	9 km
Winter chlorophyll	mg m^{-3}	Range, Min, Mean, Max	OceanColor	9 km
Spring chlorophyll	mg m^{-3}	Range, Min, Mean, Max	OceanColor	9 km
Summer chlorophyll	mg m^{-3}	Range, Min, Mean, Max	OceanColor	9 km
Fall chlorophyll	mg m^{-3}	Range, Min, Mean, Max	OceanColor	9 km
Surface temperature	°C	Range, Min, Mean, Max	GLORYS	¼°
Bottom temperature	°C	Range, Min, Mean, Max	GLORYS	¼°
Surface salinity	PSU	Range, Min, Mean, Max	GLORYS	¼°
Bottom salinity	PSU	Range, Min, Mean, Max	GLORYS	¼°
Surface current	m/s	Range, Min, Mean, Max	GLORYS	¼°
Bottom current	m/s	Range, Min, Mean, Max	GLORYS	¼°
Shear	Pa	Range, Min, Mean, Max	GLORYS	¼°

This list of variables is by no means an exhaustive list of predictors that may influence the distribution of *Geodia* spp. sponges or sponge grounds in the study area, and other variables (e.g., sea ice, wind, chemistry) are available from online data bases (EU 2013; NODC 2013). SDM studies of *Lophelia pertusa* and their reefs include a wider range of topographic variables including aspect, curvature and a bathymetric position index (Guinan *et al.* 2009; Ross and Howell 2012), as well as data on alkalinity, aragonite saturation state, salinity and concentrations of dissolved inorganic carbon, dissolved oxygen, nitrate, phosphate and silicate (Dolan *et al.* 2008; Davies *et al.* 2008; Tittensor *et al.* 2009), all derived with different accuracies and at different spatial resolutions. It is important to note that not all these predictors proved important to predict *Lophelia pertusa* distributions, and it is at least conceivable that some variables would have gained or lost importance for prediction had they been quantified differently and/or measured at

a different spatial resolution. Other potentially important predictors that we did not have data for include the substrate type and historical trawling intensity.

Distribution Modeling

Model Types

Two machine learning techniques were used to model the distribution of each response variable: random forest (RF) (Breiman 2001) and MaxEnt (ME) (Phillips *et al.* 2006). RF is a non-parametric ensemble classifier that predicts the value (here: probability of presence) of a single response variable from the values of multiple predictors. It is an effective modeling method in situations where the number of predictors is larger than the number of samples, and is widely used for distribution modeling (e.g., Lawler *et al.* 2009; Knudby *et al.* 2010). An RF model is composed of multiple regression trees (Breiman *et al.* 1984), where splits at each node in each tree are based on a random subset of the available predictors. For each response variable and study site, we trained a model using the presence/absence field data and the values of the predictors at the corresponding locations. RF model development was done in R (R Core Development Team 2012) using the "randomForest" package (Liaw and Wiener 2002). Default values were used for RF parameters. ME is a distribution modeling technique that determines the maximum entropy probability distribution given a set of presence locations and, instead of absence data, a set of randomly selected background locations, as well as the values of the predictors at those locations (Phillips *et al.* 2006). ME has been shown to perform well in comparison to other methods (Elith *et al.* 2006). ME model development was done in R using the "dismo" package (Hijmans *et al.* 2011). Default values were used for ME parameters.

Variable Elimination

To reduce problems arising from a large number of collinear predictors (Graham 2003), two approaches were used to reduce the number of predictors in the models. The first approach was to remove predictors highly correlated with others (Legendre and Legendre 2012), and the second was to remove predictors that did not contribute to reduce a model's prediction error. Variable elimination was important in our study because of the high number of collinear variables that had initially been included (e.g., the various chlorophyll-a concentration data layers) and the desire to produce interpretable models from which ecological hypotheses could be generated.

Removal of Highly Correlated Variables

For each study area, the Pearson correlation coefficients between all predictors were calculated from a complete sample of all raster cells. The two predictors with the highest correlation were then considered, and one of them eliminated. This process was repeated until no remaining variables were highly correlated, here defined as $R>0.7$.

In most cases high correlations were found between obviously related variables (i.e., the annual range of chlorophyll-a concentrations and the annual maximum chlorophyll-a concentration). When the highly correlated variables represented two metrics (mean, maximum, minimum and range) for the same variable, we preferentially eliminated the mean, then the minimum or the maximum, and then the range. Elimination of the minimum or maximum metric when these two were correlated depended on the variable in question. This preferential elimination of some metrics over others was based on the reasoning that the range (max-min), while not as refined or specific as mean, maximum and minimum, represents the variability of conditions likely to be encountered by an organism, while the maximum and minimum represent tolerance limits. For different variables, regardless of metric, we preferentially eliminated annual chlorophyll-a concentrations over seasonal ones, and preferentially kept spring chlorophyll-a concentrations over those of other seasons, based on the seasonal reproduction of *Geodia barretti* in Scandinavia (Spetland *et al.* 2007). We preferentially kept variables describing conditions at the seafloor over those describing conditions at the sea surface. We acknowledge that our preferences are subjective, but given the limited data on the ecology of deep-sea sponges in general, and *Geodia* spp. in particular, no objective alternative was available. The predictors remaining after the elimination of highly correlated variables are shown in Table 6. For both the FC and EC study areas, 23 variables of the original 50 remained after highly correlated variables had been eliminated.

Table 6. Subset of environmental predictor variables used in the SDMs. Elimination of variables was determined by their correlation coefficients.

**FC study area**		**EC study area**	
**Only R < 0.7**	**Quantification**	**Only R < 0.7**	**Quantification**
Depth	N/A	Depth	N/A
Slope	N/A	Slope	N/A
Annual chlorophyll	Min	Annual chlorophyll	Range
Winter chlorophyll	Range, Min, Mean	Winter chlorophyll	Range, Min
Spring chlorophyll	Range	Spring chlorophyll	Range, Min, Mean
Summer chlorophyll	Range, Min	Summer chlorophyll	Range, Min
Fall chlorophyll	Range, Min	Fall chlorophyll	Range, Min
Surface temperature	Range, Min	Surface temperature	Range, Min
Surface salinity	Range, Max	Surface salinity	Max
Bottom temperature	Min, Max	Bottom temperature	Max
Bottom salinity	Max	Bottom salinity	Range, Max
Surface current	Range, Min, Mean	Surface current	Range, Min
Shear	Range, Min	Shear	Range, Min, Max

Removal of Variables That Did Not Contribute to Model Performance

For each study area, response variable, and model type (RF and ME), the area under the receiver operating characteristic curve (AUC) was calculated using 10-fold cross-validation repeated 10 times for models developed using the 23 predictors (Table 6). The AUC value is a measure of model fit calculated from the combination of true-positive and false-positive rates. Its value ranges from 0 to 1 and equals the probability that the model will produce a higher presence-probability for a randomly chosen observed presence than for a randomly chosen observed absence (Fawcett 2006). AUC is a commonly used measure of model performance in SDM. Each individual variable was then temporarily removed from the model development and testing process, and AUC values were calculated again for the 23 resulting models, each model based on all but the removed variable. If the removal of any variable caused the AUC value to increase (indicating improved fit) or at least not decrease, the variable that, when temporarily removed, produced the highest AUC value, was eliminated. This process was repeated, starting with the 22 remaining variables, and so on until no variable could be removed without decreasing the resulting AUC value. Because the AUC value fluctuates between model runs as a consequence of the random splits used to define the cross-validation groups, AUC values were considered to “not decrease” if a reduction in AUC value was less than 0.005. The resulting variable combinations for each combination of study area, response variable and model type are shown in Table 7 and Table 8 for the FC and EC study areas, respectively.

Table 7. Predictor variable combinations remaining after the AUC-based variable elimination, for the FC study area under the two SDMs for each Response variable.

**Response variable \ Model**	**Random Forest**	**MaxEnt**
Sponge ground (local gear-specific thresholds)	Depth<br>Slope<br>Surface temperature, range<br>Surface salinity, range<br>Shear, range	Depth<br>Surface salinity, range<br>Bottom temperature, minimum<br>Shear, range
Sponge ground (global threshold)	As above	
Geodia spp.	Depth<br>Surface current, range<br>Surface current, minimum<br>Shear, minimum	Depth<br>Surface salinity, range<br>Bottom temperature, minimum<br>Surface current, range<br>Shear, minimum
Geodia barretti	Depth<br>Winter chlorophyll, range<br>Spring chlorophyll, range<br>Surface temperature, minimum<br>Surface salinity, range<br>Surface salinity, minimum<br>Shear, minimum	Depth<br>Surface salinity, range<br>Bottom temperature, minimum<br>Surface current, range<br>Shear, minimum
Geodia phlegraei	Spring chlorophyll, range<br>Summer chlorophyll, range<br>Summer chlorophyll, minimum<br>Surface temperature, range<br>Surface temperature, minimum<br>Bottom temperature, maximum<br>Bottom salinity, maximum<br>Shear, range	Sea surface salinity, range<br>Bottom temperature, minimum<br>Surface current, range<br>Shear, minimum

Table 8. Predictor variable combinations remaining after the AUC-based variable elimination, for the EC study area for each of the Response variables and SDMs.

**Response variable \ Model**	**Random Forest**	**MaxEnt**
Sponge ground (local gear-specific threshold)	Depth<br>Surface temperature, range<br>Bottom salinity, maximum<br>Shear, maximum	Depth<br>Summer chlorophyll, minimum<br>Surface temperature, range<br>Bottom salinity, range<br>Bottom salinity, maximum<br>Shear, range
Sponge ground (global threshold)	Depth<br>Summer chlorophyll, minimum<br>Surface temperature, range<br>Surface salinity, maximum	Depth<br>Surface temperature, range<br>Bottom salinity, range<br>Bottom salinity, maximum
Geodia spp.	Depth<br>Surface temperature, range<br>Bottom salinity, range<br>Surface current, minimum	Depth<br>Summer chlorophyll, range<br>Summer chlorophyll, minimum<br>Surface temperature, range<br>Surface temperature, minimum
Geodia barretti	Depth<br>Winter chlorophyll, range<br>Spring chlorophyll, minimum<br>Surface temperature, range<br>Shear, maximum	Depth<br>Surface temperature, range<br>Surface temperature, minimum<br>Bottom salinity, range<br>Surface current, minimum
Geodia phlegraei	Summer chlorophyll, minimum<br>Surface temperature, minimum<br>Bottom salinity, range<br>Bottom salinity, maximum	Depth<br>Surface temperature, minimum<br>Bottom salinity, range<br>Surface current, minimum<br>Shear, minimum

Model Evaluation

Model Fit for Training Area

RF and ME models were trained to predict each response variable separately for each study area, and AUC values were estimated for each combination of model type, response variable and study area using 10-fold cross-validation. Cross-validation attempts to address the problem of how a model will perform on a future data set by splitting the

available data into a number of disjoint groups of equal size, train the model on a combination of all but one of them, and test the model performance by comparing predictions for the last group with their observed values. This is repeated using each group as the test group and calculating the mean of a measure of fit for each group, in our case using the AUC value. Because cross-validation estimates of model performance depend on the exact split of the full data set into disjoint groups, we repeated the calculations 10 times for each combination of model, response variable and study area, and calculated the mean of the results. We used the absence data to calculate AUC values for both the RF and ME models, making the two values directly comparable. Our ME AUC values thus differ from those that would have been calculated using the ME software GUI. Model predictions were also applied to the predictors to produce mapped predictions for each study area and response variable, and compared between the two model types.

Model Extrapolation

For each model type, response variable and set of predictors, AUC values were calculated for models trained for the FC study area and applied to the EC study area, and mapped predictions of presence probabilities were produced. These AUC values and maps were then compared with models both trained for and applied to the EC study areas, to assess the effect of extrapolating the models beyond the area they were trained for.

Partial Dependence Plots

Insight into the effect variation in each predictor has on predictions of the response variable can be gained from partial dependence plots, which can be created using two different but related approaches. Conceptually the simplest is to train a model using only a single environmental predictor, and then observe the predictions made by this model using the range of input values that exist for the predictor. We call this the single-variable approach. Another approach is to train a model using all the available predictors, and then observe the predictions made by this model when all predictors but one are kept at their mean value, while using the range of input values that exist for the predictor. We call this the multi-variable approach. Both these approaches are implemented in the ME GUI (Phillips *et al.* 2006); we have implemented them in R to allow their application to both the RF and ME models.

Variable Importance

Given a fitted model, we assess the relative importance for predictions of each predictor by permuting test set values of each individual variable. We then quantify the resulting reduction in model fit, calculated as the AUC value of the model when tested against unpermuted data minus the AUC value of the model when tested against permuted data (delta-AUC). The permutation is repeated 10 times for each of the 10 cross-validation

partitions in each of the 10 repetitions, to obtain results that are independent of particular splits of the data set for cross-validation and particular variable permutations (Knudby *et al.* 2010). The permutation approach to assessment of variable importance is also found in the RF implementation in R (Liaw and Wiener 2002); we have implemented it independently to allow its application to both the RF and ME models. Although both model types consider interactions between predictors, we did not specifically investigate such interactions through the partial dependence plots or the variable importance measure.

RESULTS AND DISCUSSION

Due to the combinations of two study areas, two model types, five response variables, and the two variable elimination approaches that yielded three different sets of predictors, the study has created a large set of results. The following section will provide all results when feasible and examples, focusing on *Geodia* spp. presence in the FC study area, when providing all results is not feasible. The complete set of results is available in the Appendix when not provided in this section.

Variable Elimination

The AUC values produced by all models are shown in Table 9 and Table 10, where the top row of each cell corresponds to a model developed using the original 50 variables (Table 5), the centre row corresponds to a model developed using the 23 variables remaining after elimination of variables that were highly correlated (Table 6), and the bottom row corresponds to a model developed using the variables remaining after the AUC-based variable elimination. Model performance in all cases was excellent (AUC > 0.9), showing that the distributions of all the response variables are highly structured by their environment as quantified by our predictors, and that the models are able to emulate that structure.

Table 9. AUC values obtained with models based on the original 50 variables (top row in each cell), the 23 variables remaining after elimination of variables that were highly correlated (centre row in each cell), and the variables remaining after the AUC-based variable elimination, for the FC study area.

**Response variable \ Model**	**Random Forest**	**MaxEnt**
Sponge ground (local gear-specific thresholds)	0.985<br>0.986<br>0.986	0.980<br>0.955<br>0.954
Sponge ground (global threshold)	As above	
Geodia spp.	0.945<br>0.941<br>0.976	0.955<br>0.955<br>0.976
Geodia barretti	0.983<br>0.947<br>0.974	0.970<br>0.972<br>0.976
Geodia phlegraei	0.959<br>0.952<br>0.980	0.957<br>0.954<br>0.975

Table 10. AUC values obtained with models based on the original 50 variables (top row in each cell), the 23 variables remaining after elimination of variables that were highly correlated (centre row in each cell), and the variables remaining after the AUC-based variable elimination, for the EC study area.

**Response variable \ Model**	**Random Forest**	**MaxEnt**
Sponge ground (local gear-specific threshold)	0.966<br>0.967<br>0.964	0.960<br>0.955<br>0.944
Sponge ground (global threshold)	0.977<br>0.978<br>0.973	0.970<br>0.971<br>0.948
Geodia spp.	0.982<br>0.985<br>0.987	0.980<br>0.975<br>0.973
Geodia barretti	0.988<br>0.989<br>0.988	0.983<br>0.982<br>0.974
Geodia phlegraei	0.971<br>0.969<br>0.982	0.966<br>0.975<br>0.975

Models developed using the variable combinations remaining after the AUC-based variable elimination have AUC values that are typically similar to those resulting from the models based on a larger number of variables, supporting our two-step variable elimination approach which retained the most influential predictors. Notable exceptions include the ME sponge ground models, where model relative performance deteriorates as variables are eliminated. This is possible when several eliminated variables individually reduce the AUC value by less than 0.005. Other notable exceptions include models of *Geodia* spp. and *G. phlegraei*, where models developed with the smallest number of variables produce the highest AUC values for both models types in the FC study area, and for RF models in the EC study area.

Model Evaluation

Model Fit for Training Area

The RF models perform similarly to or better than the ME models, with the largest differences between the two models seen for the EC study area. We suggest that the superior performance of the RF models is a result of the information content in the absence data, used to train the RF but not the ME models, the latter being specifically developed for use with presence-only data. If false absences in the field data were a substantial problem this would decrease the information content in the absence data points, and reduce the advantage of the RF models over the ME models. For both study areas, the smallest differences in AUC values between the two models are seen for the genus- and species-level models. Differences in performance between the two model types could also be related to the different algorithm employed by each model type. A stricter analysis of the information content in the absences could be done by training RF and/or ME models with and without absences, to compare AUC values within each model type instead of across the two models.

In addition to differences in AUC values, the two models also differ in their spatial predictions. Figure 4, shows *Geodia* spp. predictions made by ME and RF, respectively, along with the observed presence and absence locations used to train the two models. The ME model has a generous area of high probability which includes all of the known presences on the continental slopes, particularly in the northern part of the study area, but it also includes large areas where the sponges are absent in the field data. The RF model, on the other hand, predicts tighter distribution zones with less area of intermediate probability. This is supported by *in situ* video and camera observations which show sharp boundary transitions, at least on the Sackville Spur in the north of Flemish Cap. The RF model also indicates a high probability of *Geodia* spp. in the deep waters in this area. Eight of the ten deepest field data locations show *Geodia* spp. presence, including the six deepest (depths 1827-2201 m), which provides support to the RF model's extrapolation at depth. Further validation of which model produces the best predictions, especially in deeper areas, will require additional field data. Other differences between the two models include the predicted presence probability for *Geodia* spp. in the Flemish Pass between Flemish Cap and Grand Bank in the FC area with ME. Beazley *et al*. (2013) report that

Geodia spp. are present but not abundant in this area which is dominated by the glass sponge *Asconema* sp. and the demosponges of the family Axinellidae. Murillo *et al*. (2012) report low sponge biomass in this area. Note that the AUC values for the two models are identical for this response variable and study area (Table 9), despite the differences seen in the predictions.

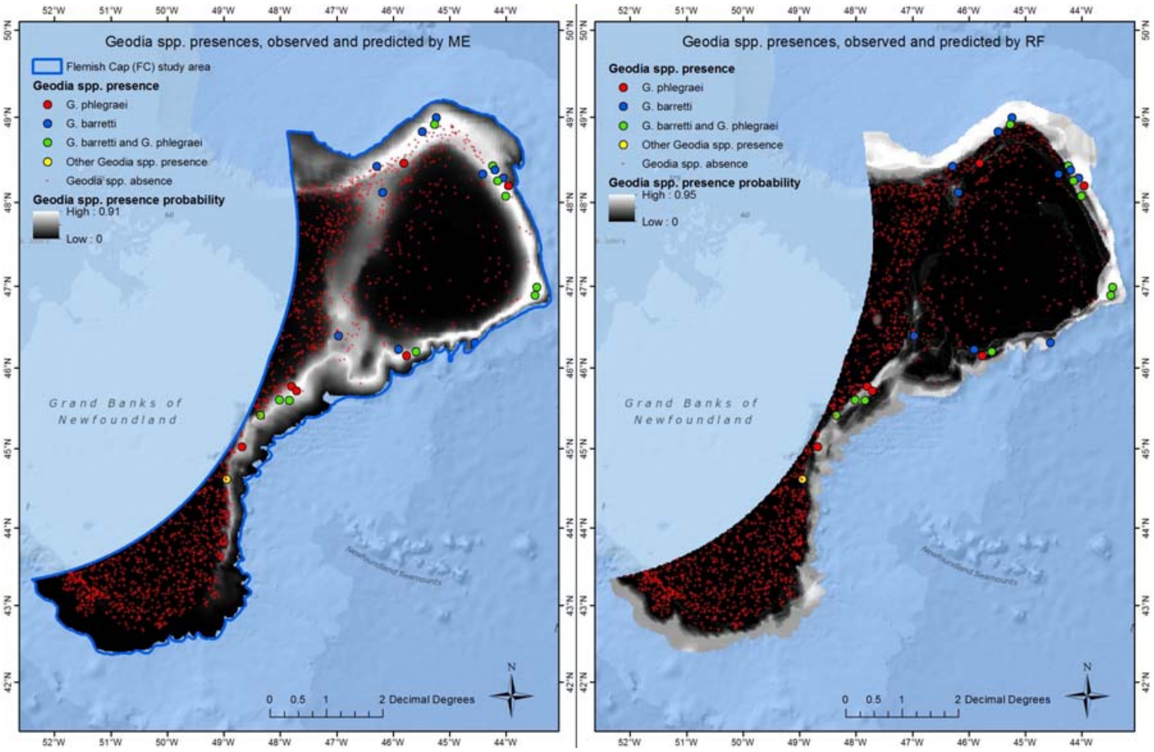

Figure 4. *Geodia* spp. locations in the FC study area, observed (coloured circles) and predicted probability by ME (left) and RF (right).

Model Extrapolation

Models trained on data from the FC study area and tested with data from the EC study area (Table 11) all have poorer fits than the models trained and tested with data from a single area (Table 9 and Table 10), which indicates that the relationships between the response variables and the predictors differ between the FC and EC study areas. This is not a surprising result given the large geographical extent of the EC study area, which includes a range of biogeographic zones, as opposed to the small FC study area (Figure 1).

Table 11. AUC values obtained when models trained for the FC study area were applied to the larger EC study area.

Response variable \ Model	Random Forest	MaxEnt
Sponge ground (local gear-specific threshold)	0.898<br>0.915<br>0.911	0.763<br>0.816<br>0.780
Sponge ground (global threshold)	0.920<br>0.933<br>0.832	0.808<br>0.865<br>0.832
Geodia spp.	0.941<br>0.917<br>0.743	0.774<br>0.789<br>0.581
Geodia barretti	0.944<br>0.913<br>0.807	0.921<br>0.888<br>0.739
Geodia phlegraei	0.955<br>0.927<br>0.890	0.919<br>0.894<br>0.593

The RF models consistently outperform the ME models when extrapolated beyond the area they were trained for (Table 9 and Table 10). This may be related to ME's sampling of pseudo-absences from the background (the study area used for training), which changes when the model is extrapolated to a new study area. Based on these results the RF model is recommended over ME for extrapolation with our data.

For the RF models of *Geodia* spp., *G. barretti* and *G. phlegraei*, the complete set of (50) predictors (top rows in Table 11) obtain better fits when extrapolated than those trained with sets of predictor variables reduced through variable elimination, while for the RF models of sponge grounds the set of predictors remaining after removal of highly collinear predictors (centre rows in Table 11) obtained the best fits. A similar pattern is seen for the ME models, with the exception of the *Geodia* spp. model. Models trained on the smallest sets of variables (bottom rows in Table 11) provide the worst fit for most models, which suggests that the AUC-based variable elimination approach results in models over-fitting to the training data, and consequently to the training area.

The RF models of *Geodia* spp., *G. barretti* and *G. phlegraei* provide better fits than models of sponge grounds. This may be attributed to the nature of the response variables; the *Geodia* genus and species are well-defined biological entities whose composition and environmental niches can be expected to remain largely spatially constant, while sponge grounds as defined using a weight/tow threshold are composed of different species with potentially varying environmental niches. Using the complete set of predictors, the fits (AUC values) obtained from the extrapolated RF models of *Geodia* spp., *G. barretti* and *G. phlegraei* are close to those obtained from the models trained on the EC study area; this is less the case for models of sponge grounds (compare Table 10 and Table 11).

The difference between using training data from the FC and EC study areas, when making predictions for the EC study area, is illustrated by mapped predictions of *Geodia* spp. presence probability. Figure 5 shows mapped predictions of *Geodia* spp. presence probability from the RF model trained with the smallest set of predictors using data from the EC study area. Most of the observed presence locations are correctly identified as having high presence probability, and most observed absence locations have low presence probability. The unsampled Labrador slope shows high *Geodia* spp. presence probability (**1** in Figure 5), as does the unsampled deep part of Baffin Bay (**2** in Figure 5). Figure 6 is comparable to Figure 5 except predictions are made from the RF model trained on the FC study area using the complete set of predictors. The observed presence locations in the FC study area are correctly predicted as having high presence probability, which is not surprising given that the model was trained on data from the FC study area. However, the grouping of observed presences near Hudson Strait (**3** in Figure 6) is predicted as having only intermediate presence probability. This may partly be due to the shallower depths at which *Geodia* spp. are found at these latitudes; the mean depths of *Geodia* spp. presence observations located above and below 60°N are 802 m and 1290 m respectively, with the shallowest *Geodia* spp. below 60°N found at a depth of 868 m. However, the partial dependence of *Geodia* spp. on depth for the two models, shown in Figure 7, illustrate that the modeled influence of depth is similar between the two models, and that predicted *Geodia* spp. presence probabilities are low for depths around 1290 m for both models. Depth alone can therefore not explain the difference between the predictions of the two models. However, the partial dependences of *Geodia* spp. on bottom salinity range and minimum surface current in the model trained on the EC study area both show a spike in predicted presence probability when using the single-variable approach (red lines in Figure 8) for values around 0.2 PSU bottom salinity range and around 0.015 m/s minimum bottom current, which correspond to the environmental conditions found in the area of the observed presences near Hudson Strait (**3** in Figure 6) as well as those found in the northernmost grouping of observed presences (**4** in Figure 6). These specific environmental conditions were incorporated in the model trained on the EC study area, and lead to high presence probability predictions as seen in Figure 5. The same spikes are not seen in the corresponding dependence plots of the model trained on the FC study area (red lines in Figure 9), which did not have the presence observations from these areas available for model training.

No predictions are available for the northernmost grouping of observed presences (**4** in Figure 6) because predictions for *Geodia* spp. are restricted to the area for which data were available for winter chlorophyll concentrations, as this predictor is included in the model. The data on chlorophyll concentrations are derived directly from satellite observations of reflected sunlight on cloud-free days, and their availability for the northern part of the EC study area in the winter months is therefore limited.

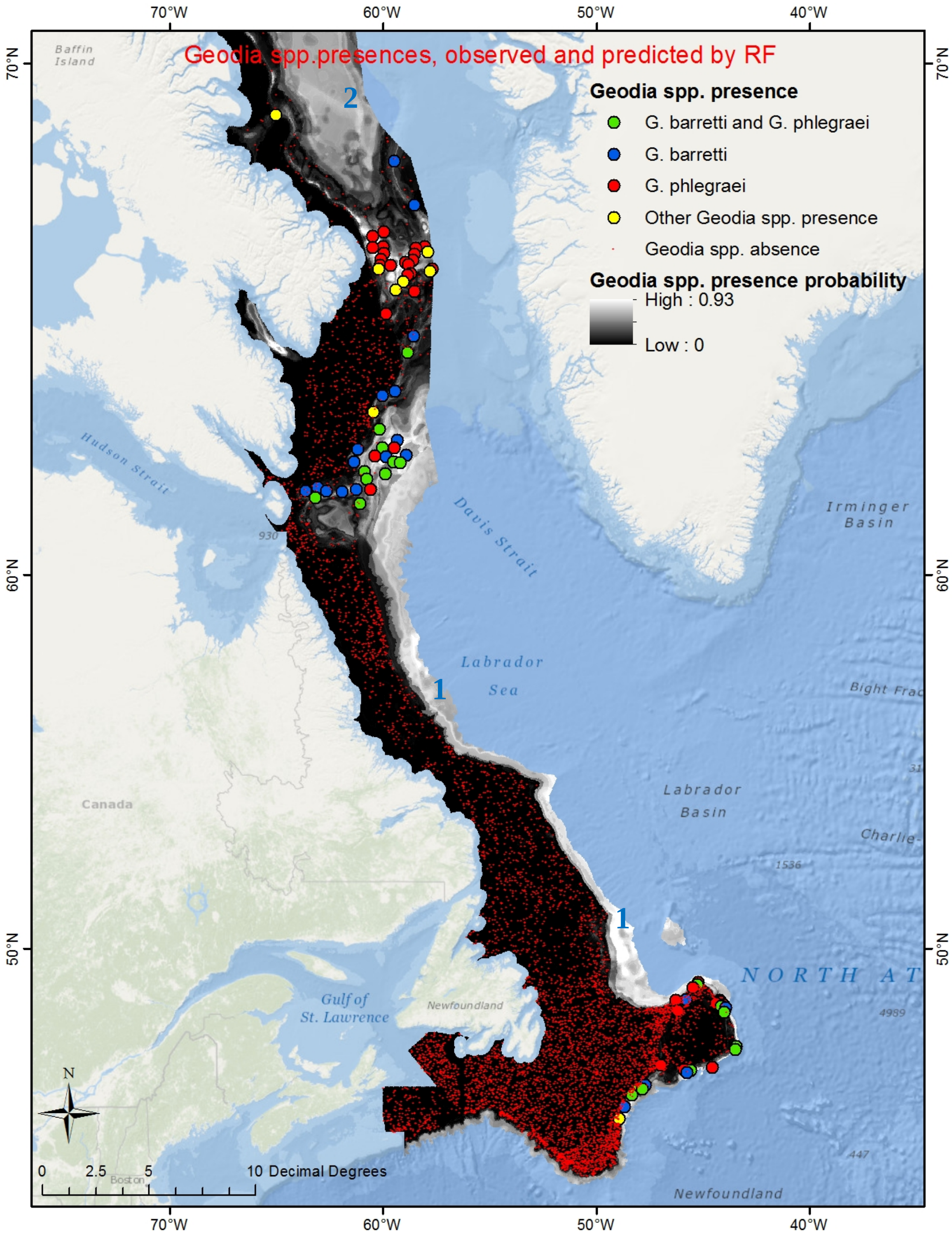

Figure 5. *Geodia* spp. locations in the EC study area, observed (coloured circles) and predicted probability by the RF model (grayscale).

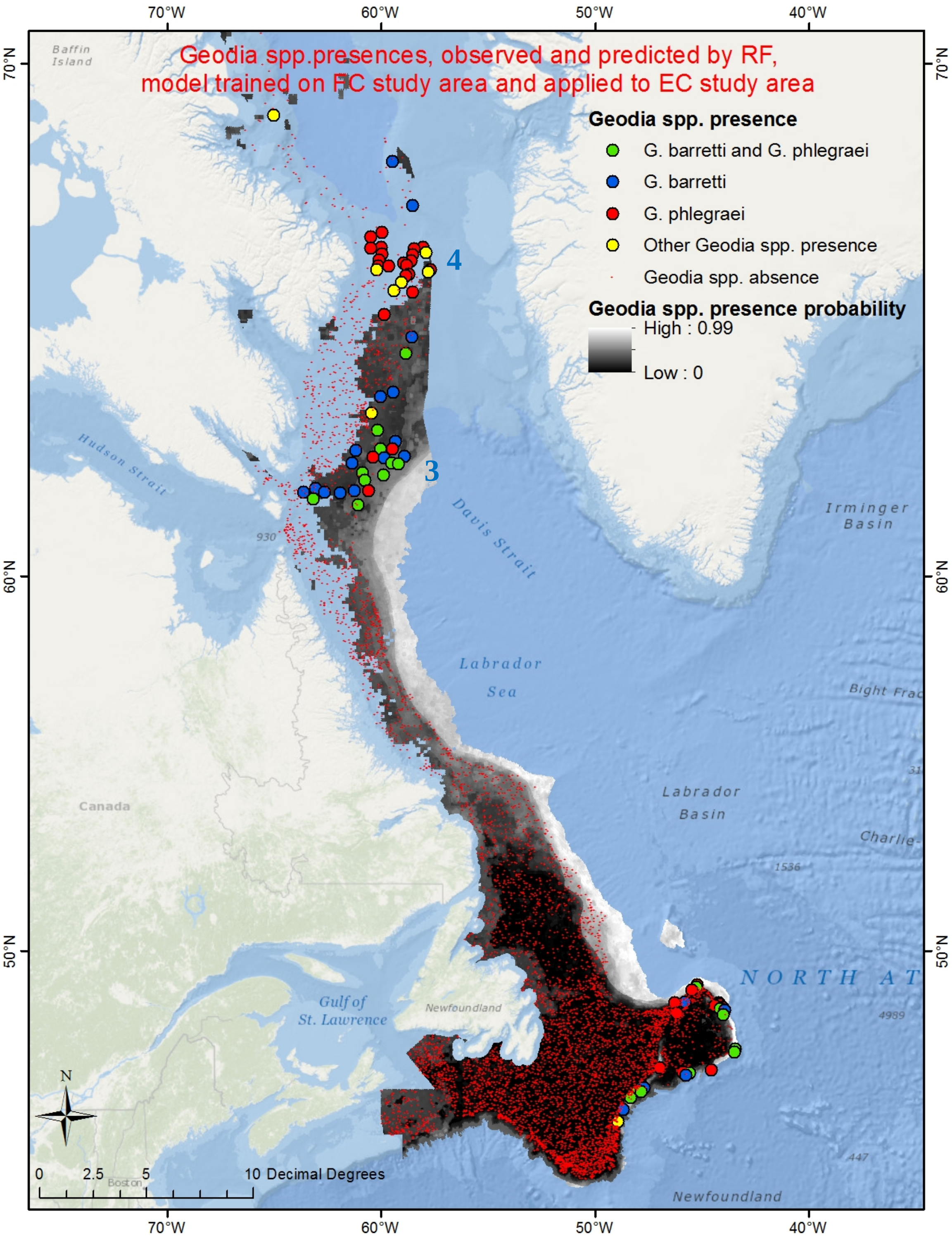

Figure 6. *Geodia* spp. locations in the EC study area, observed (coloured circles) and predicted probability by the RF model extrapolated from the FC study area to the EC study area (grayscale).

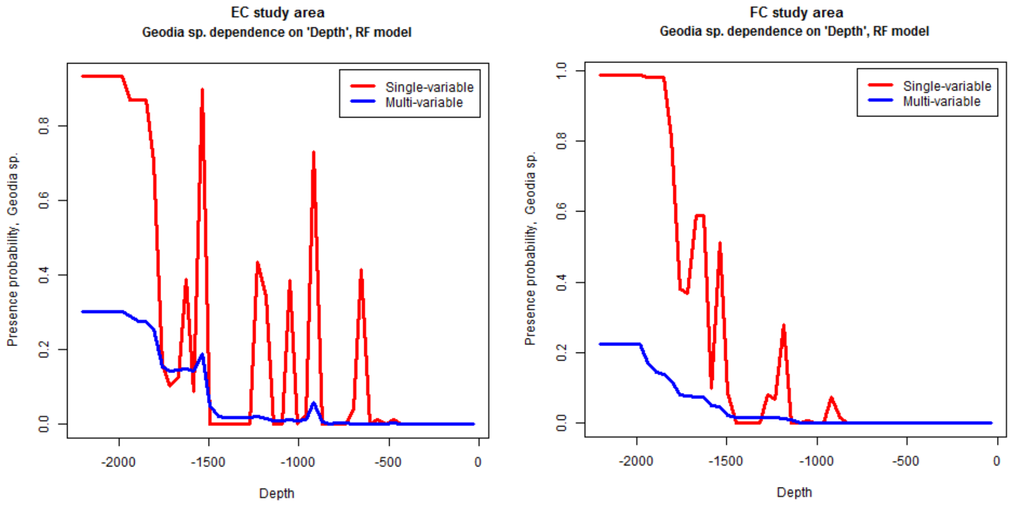

Figure 7. Partial dependence of *Geodia* spp. on depth, RF model, EC study area (left) and FC study area, full set of predictors (right).

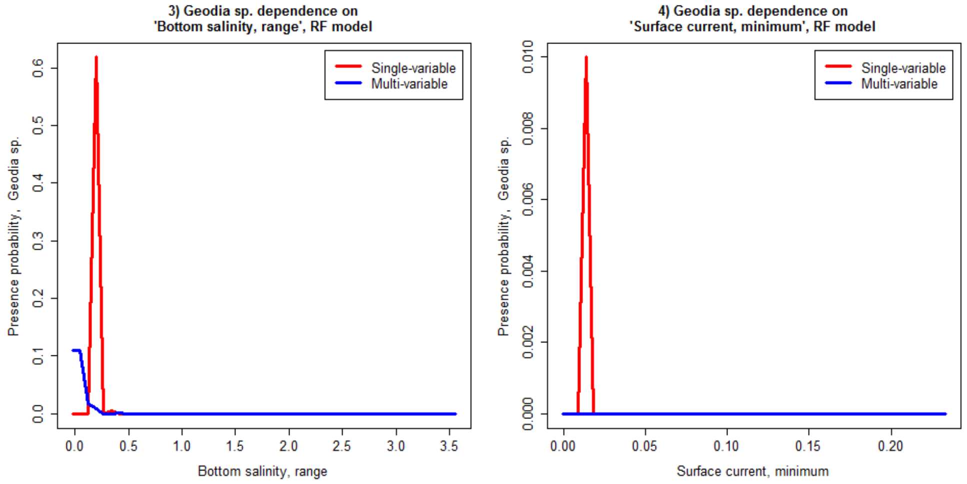

Figure 8. Partial dependence of *Geodia* spp. on bottom salinity range (left) and minimum surface current (right), RF model, EC study area.

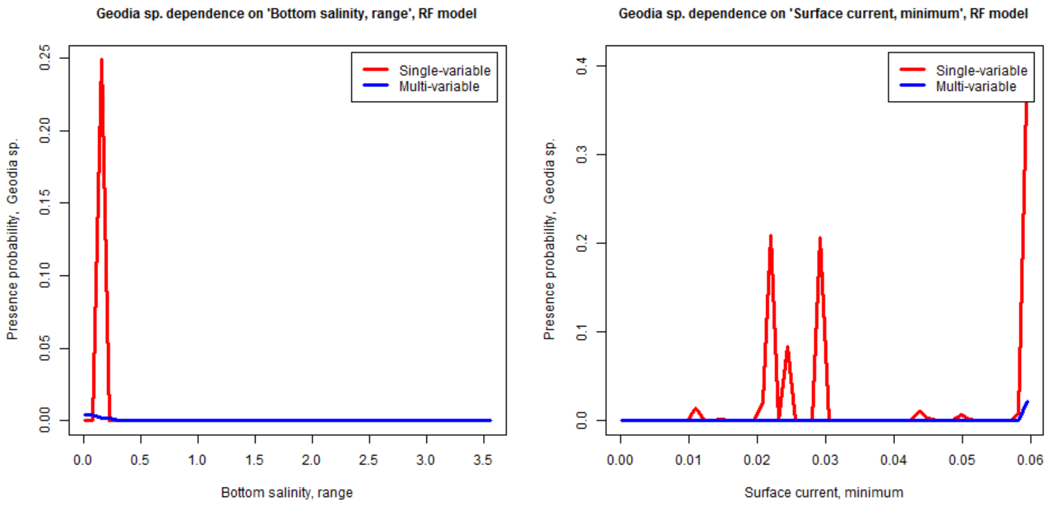

Figure 9. Partial dependence of *Geodia* spp. on bottom salinity range (left) and minimum surface current (right), RF model, FC study area, complete set of predictors.

Partial Dependence Plots

Two examples of the partial dependence plots for the predictors of *Geodia* spp., FC study area, are shown in Figure 10 for the RF model and in Figure 11 for the ME model, both based on the set of predictors available after both variable elimination approaches were applied. The dependence of *Geodia* spp. on depth is illustrative of the differences both between the two approaches and the two model types. For RF (Figure 10), with the multi-variable approach we see that *Geodia* spp. presence probability is 0.0 for depths less than approximately 800 m, and then increases to above 0.5 at depths of more than 2000 m (note that depths have negative values, 0 being the sea surface). In other words, depth alone cannot generate very high presence probabilities when the other predictors are kept at their mean value. With the single-variable approach the *Geodia* spp. presence probability is also 0.0 for depths less than 800 m, but reaches almost 1.0 at depths of more than approximately 1800 m. The difference between the two approaches illustrate that in reality the other predictors do not have their mean values in deep waters, but rather they have values that, in the model, contribute to high *Geodia* spp. presence probability. The dependence plots are ‘spiky’ and less interpretable for the three remaining predictors, except for a trend of higher *Geodia* spp. presence probability for higher values of ‘Shear, minimum’.

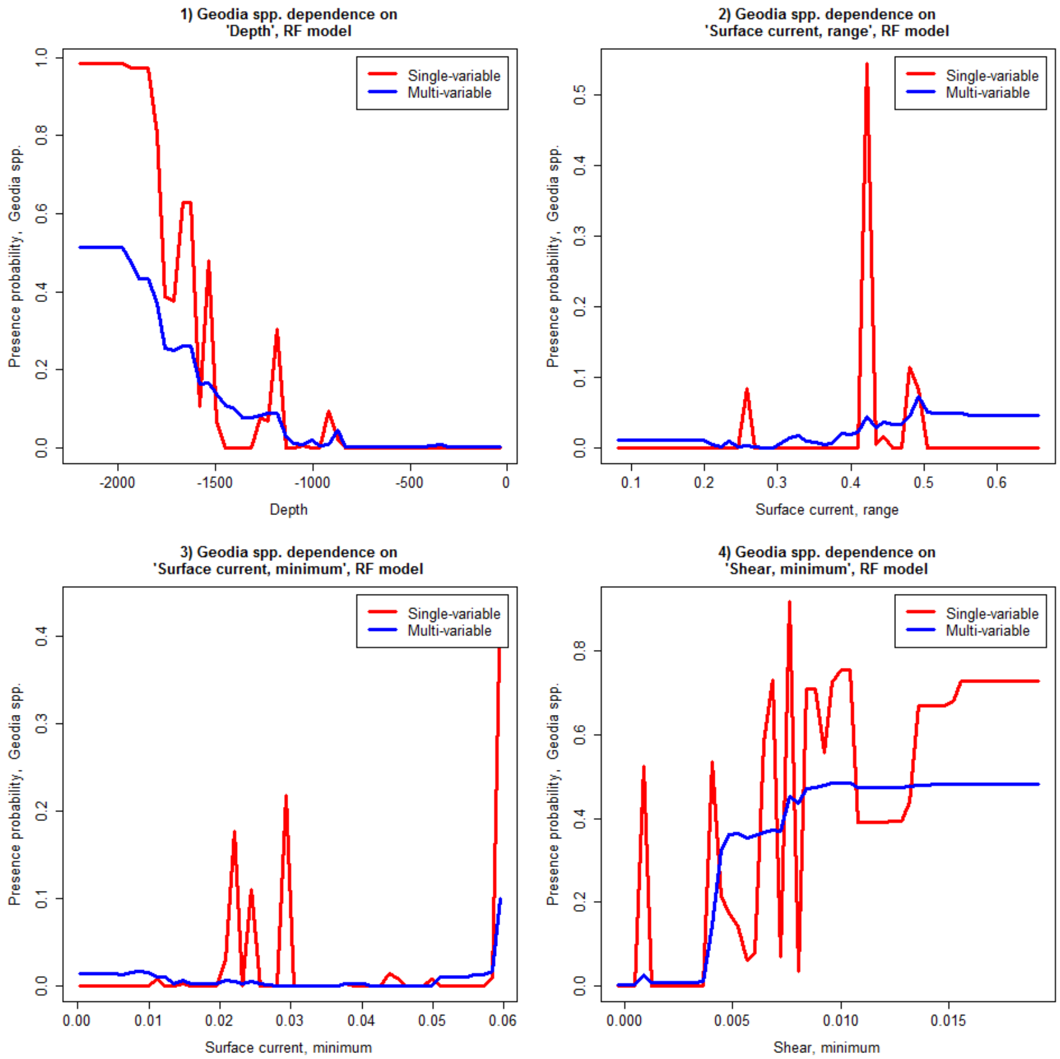

Figure 10. Dependence plots including both the single-variable and multi-variable approaches for the four predictors of *Geodia* spp., using the RF model for the FC study area.

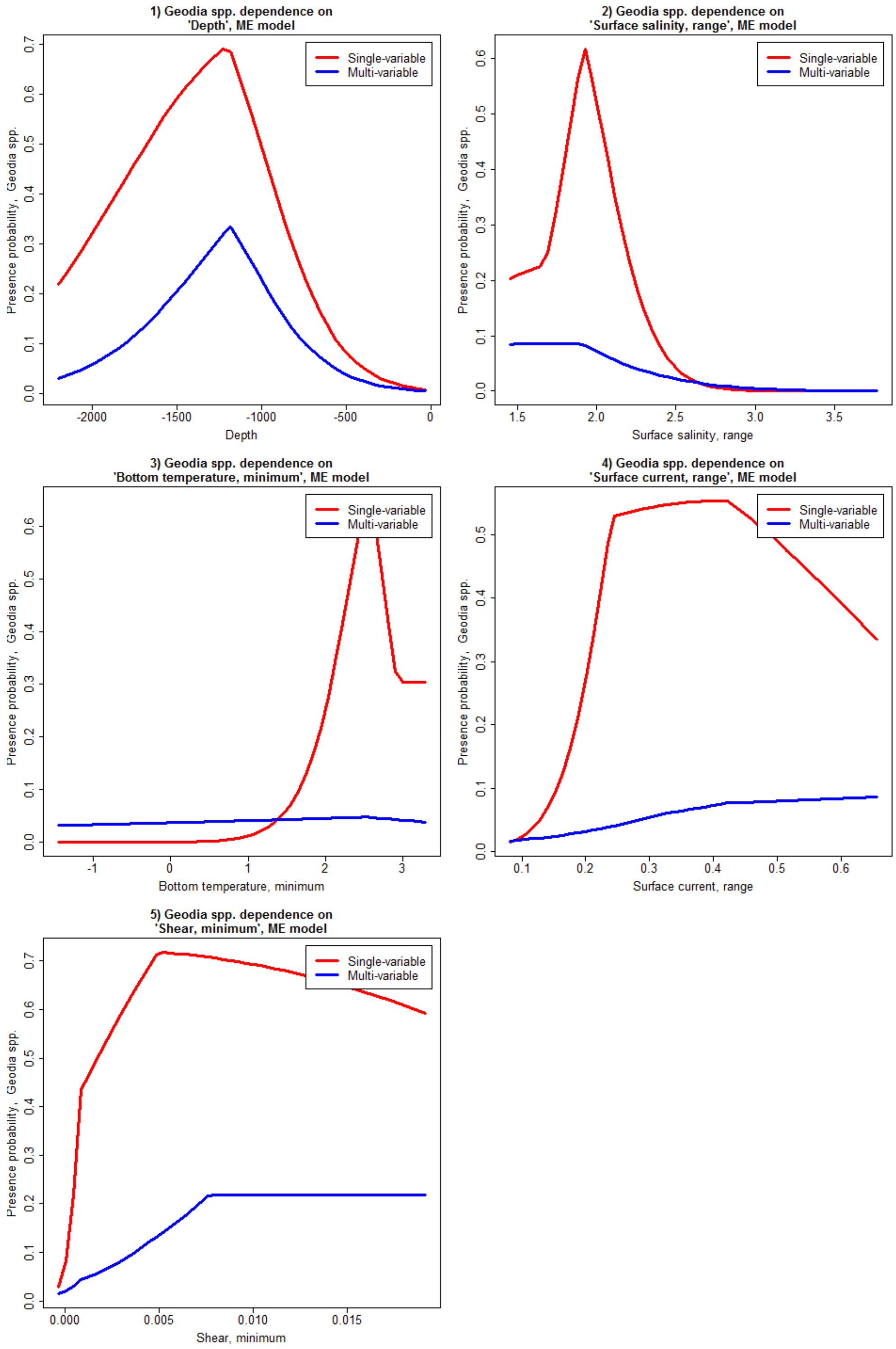

Figure 11. Partial dependence plots including both the single-variable and multi-variable approaches for the five predictors of *Geodia* spp., using the ME model for the FC study area. Top left: Depth. Top right: Surface salinity, range. Centre left: Bottom temperature, minimum. Centre right: Surface current, range. Bottom left: Shear, minimum.

For ME (Figure 11), the dependence of *Geodia* spp. on depth differs between the two approaches as it did for RF. At very shallow depths *Geodia* spp. presence probabilities are 0.0. They then increase until depths of 1200 m, after which they decrease again. As for RF, the single-variable approach reaches higher presence probabilities than the multi-variable approach, although the two curves have the same overall shape. The remaining plots are smoother than those from the RF model, and suggest that optimal conditions for *Geodia* spp. presence can be found in areas of intermediate depth (1000-1500 m), areas with a low salinity range (1.5-2 PSU), high minimum bottom temperature (2-3 °C), surface current range above 0.2 m/s and high minimum shear (>0.005 Pa). However, the different variables used in the RF and ME models and the differences in the partial dependence plots, which exist despite the very good fit of both models (AUC for both is 0.976 (Table 9)), make it hard to draw conclusions about ecological relationships. The information presented in the partial dependence plots should therefore be interpreted cautiously.

Variable Importance

Examples of variable importance results for RF and ME models of *Geodia* spp. presence, FC study area, are shown in Table 12. As is the case for the partial dependence plots, the different results for each model type, despite the very good fit of both models, makes it hard to draw conclusions about which variables truly have the greatest influence on the suitability of an area for *Geodia* spp. presence. Depth and minimum shear have the highest delta-AUC values for the RF model, indicating that, of the four predictors in that model, they have the strongest influence on predictions. These two predictors are also important in the ME model, as is surface salinity range. Surface current range has low importance for both models despite being included in both.

Table 12. Variable importance results, RF and ME models, *Geodia* spp. presence, FC study area.

**Random Forest**		**MaxEnt**	
**Predictor**	**Delta-AUC**	**Predictor**	**Delta-AUC**
Depth	0.105	Depth	0.152
Shear, minimum	0.068	Surface salinity, range	0.091
Surface current, minimum	0.032	Shear, minimum	0.066
Surface current, range	0.017	Surface current, range	0.009
		Bottom temperature, minimum	0.002

Interpretation is further complicated by a comparison with variable importance results from the EC study area, shown in Table 13. Here depth is of secondary importance in the RF model, while it is 7.5 times more important than any other variable in the ME model. In addition, minimum shear is not included in either model as it was eliminated during the AUC-based variable elimination. However, the surface temperature range, absent

from the models for the FC study area, is now the most importance predictor in the RF model, and the second-most important predictor in the ME model.

Table 13. Variable importance results, RF and ME models, *Geodia* spp. presence, EC study area.

Random Forest		MaxEnt	
**Predictor**	**Delta-AUC**	**Predictor**	**Delta-AUC**
Surface temperature, range	0.104	Depth	0.326
Depth	0.049	Surface temperature, range	0.043
Bottom salinity, range	0.045	Summer chlorophyll, minimum	0.030
Surface current, minimum	0.019	Summer chlorophyll, range	0.028
		Surface temperature, minimum	0.003

Global vs. Local Gear-Specific Thresholds for Defining Sponge Grounds

Based on a comparison of the AUC values, both models perform marginally better when developed with, and applied to, sponge grounds defined using a single global threshold than when developed with the local gear-specific thresholds (Table 10). The single global threshold enables the models to predict environmental conditions that allow a specific density of sponges to develop and persist in a location (in our case the density that corresponds to 70 kg/tow), as opposed to predicting environmental conditions that allow locally significant sponge densities to develop and persist. The 70 kg/tow threshold will underestimate the distribution of sponge ground habitat as defined using the local gear-specific thresholds in areas of the Eastern Arctic sampled by the Cosmos and Campelen gears (Table 1) and on the Scotian Shelf, while overestimating the area on the Newfoundland and Labrador shelves and slopes (Table 1).

The difference between the two different approaches to defining sponge grounds is illustrated by their respective observed and predicted distributions. Sponge grounds defined using the local gear-specific thresholds are shown in Figure 12, while sponge grounds defined using the global threshold are shown in Figure 13. Results from using the local gear-specific thresholds are generally more consistent with known occurrences of the sponge grounds (as defined using these thresholds) but cannot predict the presence of sponge grounds on the portion of the Scotian Shelf (**1** in Figure 12) which is at the southernmost limit of the range of *Geodia*, which are the major constituents of the northern sponge grounds. When the global threshold is applied the northernmost sponge grounds have low predicted presence probability (**2** and **3** in Figure 13). This is partly because several of these observations have values below 70 kg/tow, and also due to the shallower depths at which sponge grounds are found in the Arctic bioregion; the mean depths of sponge grounds (as defined by the global threshold) located above and below

60°N are 616 m and 1179 m respectively. The partial dependence plots for sponge grounds (global threshold, RF model, EC study area) are shown in Figure 14, where it is clear that areas with depths around the mean value for sponge grounds above 60°N will be predicted to have very low presence probabilities. The inability of a single model to adequately describe the influence of depth on sponge ground presence probability for the whole EC study area may be attributed to depth not being a causal predictor, but rather having influence on predictions through collinearity with other predictors that influence sponge ground development. If the relationship between depth and these other predictors changes substantially through space, a single model for which depth is an important predictor will not produce good predictions across the study area.

Variable importance results for the models of sponge ground presence for the EC area are shown in Table 14 (local gear-specific thresholds) and Table 15 (global threshold). Both RF models have depth as the dominant predictor, with delta-AUC values 2-3 times that of the second-most important variable, which in both models is the range of surface temperatures. Depth is also not the most important variable for the one ME model (local gear-specific thresholds), while the range of bottom salinity is the most important variable in the other ME model (global threshold). The range of bottom salinity is largely uncorrelated with depth in the EC study area (R=-0.095) so the importance of this variable in the ME model is unlikely to be an artifact of variable selection with collinear variables. The range of surface temperatures is also included in both ME models.

Table 14. Variable importance results, sponge grounds (local gear-specific thresholds), EC study area.

Random Forest		MaxEnt	
**Predictor**	**Delta-AUC**	**Predictor**	**Delta-AUC**
Depth	0.152	Depth	0.148
Surface temperature, range	0.058	Bottom salinity, maximum	0.047
Bottom salinity, maximum	0.045	Bottom salinity, range	0.045
Shear, maximum	0.038	Shear, range	0.027
		Surface temperature, range	0.024
		Summer chlorophyll, minimum	0.014

Table 15. Variable importance results, sponge grounds (global threshold), EC study area.

Random Forest		MaxEnt	
**Predictor**	**Delta-AUC**	**Predictor**	**Delta-AUC**
Depth	0.156	Bottom salinity, range	0.135
Surface temperature, range	0.079	Surface temperature, range	0.065
Surface salinity, maximum	0.057	Bottom salinity, maximum	0.060
Summer chlorophyll, minimum	0.033	Depth	0.046

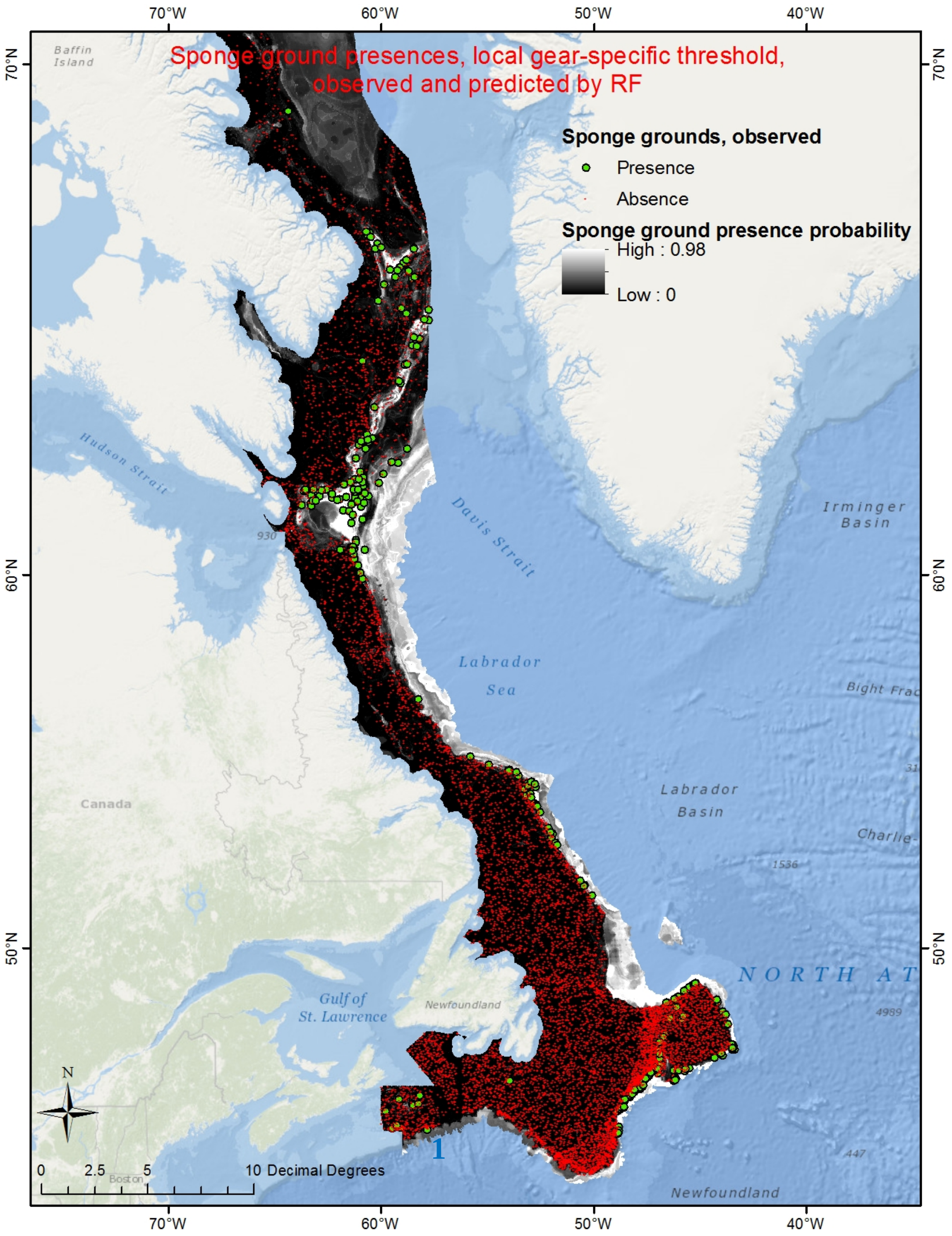

Figure 12. Sponge grounds of the EC study area as defined using local gear-specific thresholds, observed (green circles) and predicted probability by the RF model (greyscale).

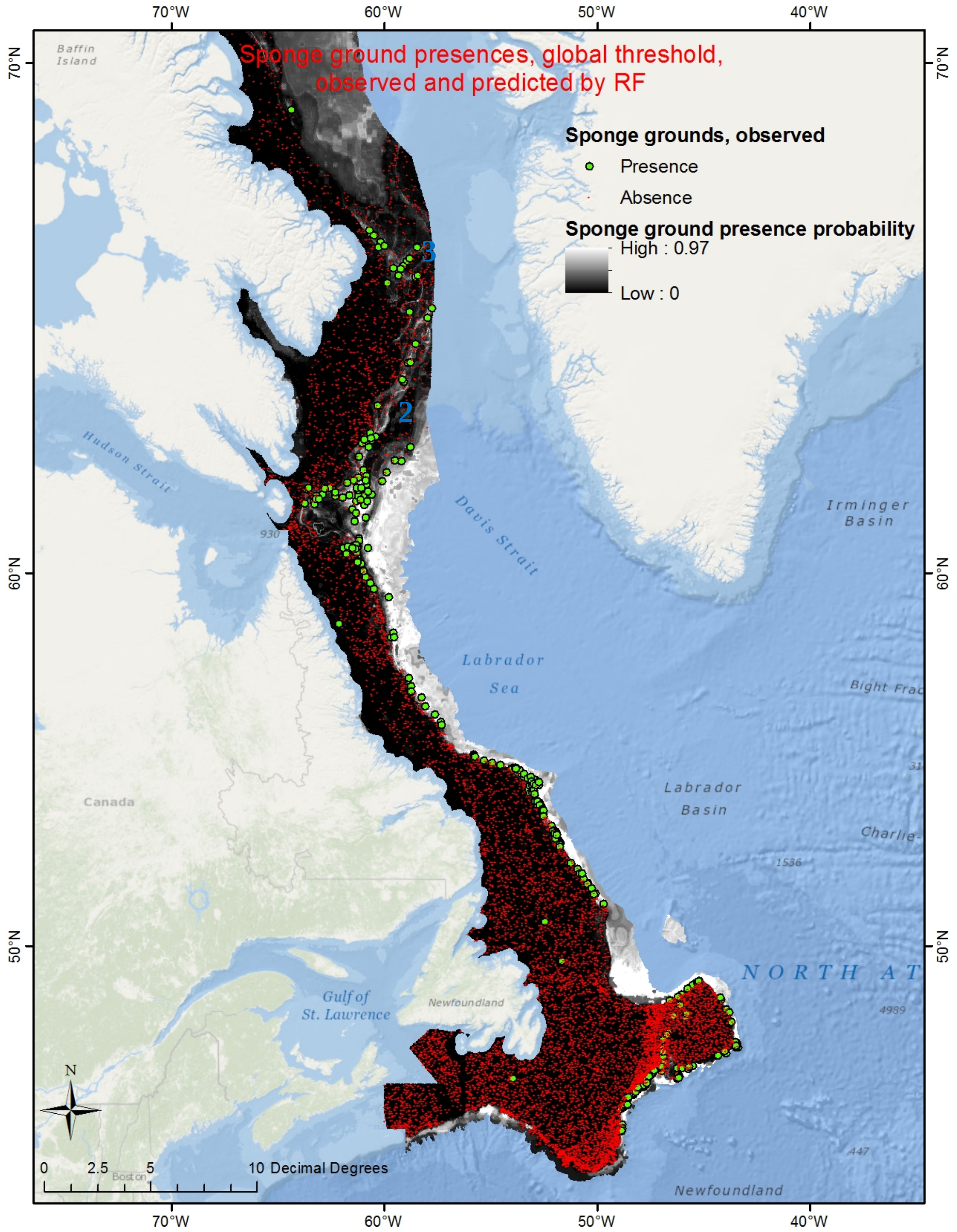

Figure 13. Sponge grounds of the EC study area as defined using global thresholds, observed (green circles) and predicted probability by the RF model (greyscale).

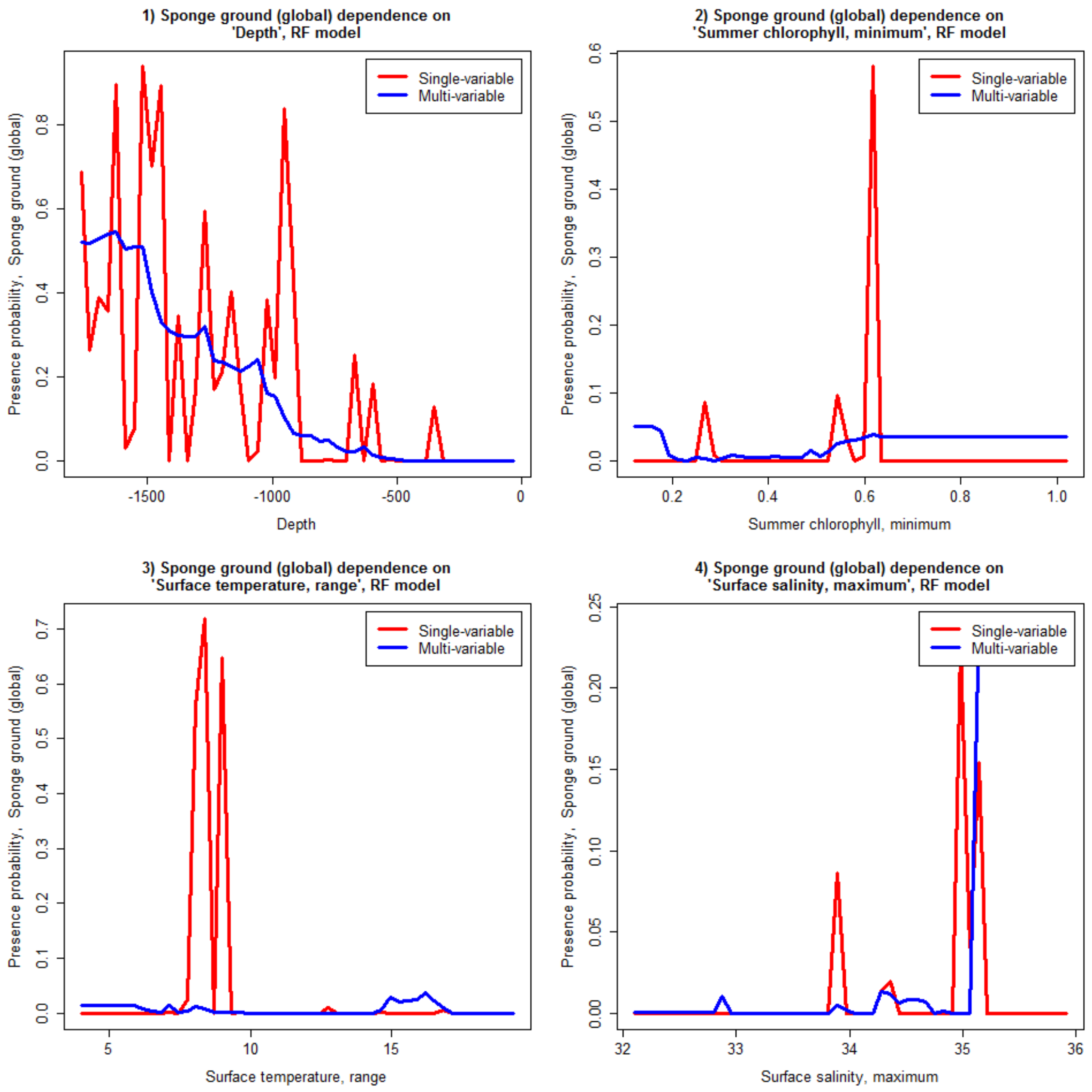

Figure 14. Partial dependence plots including both the single-variable and multi-variable approaches for the four predictors of sponge grounds as defined using the global threshold, RF model, EC study area. Top left: Depth. Top right: Summer chlorophyll, minimum. Bottom left: Surface temperature, range. Bottom right: Surface salinity, maximum.

<u>Species- vs. Genus-Level Response Variables</u>

Models of *Geodia* spp. presence have very similar AUC values to models of *G. barretti* and *G. phlegraei* presences, suggesting that the environmental conditions conducive to settlement and growth of the five different *Geodia* spp. are sufficiently similar for their distribution to be modeled as a single entity. This is contrary to what would be expected in the presence of inter-specific competition and species-specific niche development. One potential explanation for this is that there is a correspondence between the spatial resolution of the environmental data layers (0.017°) and the “thematic resolution” of

niche detail for which they are able to describe a spatial distribution, i.e. the different *Geodia* species may have similar habitat requirements in terms of what can be resolved from the spatial data layers used as predictors in our study, and the spatial expression of niche differentiation between the species may only show at a higher spatial resolution. If so, the similar model fits produced for genus- and species-level response variables suggest that we have only managed to resolve their common environmental niche. Given the limited knowledge of the biology and ecology of the species in question, this must currently remain a hypothesis. Another factor that can influence model fit is the larger number of presence observations at the genus-level than at the species-level, which may improve the ability of both model types to fit to the training data.

Variable importance results for the models of genus and species level sponge presence for the EC study area are presented in Tables 16-18. For the *Geodia* genus (Table 16), depth and the range of surface temperature are the two most important variables for both RF and ME models, while the variables of less importance vary between model types. Depth is the strongest predictor of *G. barretti* presence for both model types; the range of surface temperature is included in both models but shows low delta-AUC values. Results for *G. phlegraei* are very different. The range of bottom salinity is the most important predictor for both model types, with delta-AUC values ~3 times higher than that of the second-most important variables. Depth is not included at all as a predictor in the RF model, and is only of secondary importance in the ME model.

Table 16. Variable importance results, *Geodia* spp., EC study area.

**Random Forest**		**MaxEnt**	
**Predictor**	**Delta-AUC**	**Predictor**	**Delta-AUC**
Surface temperature, range	0.104	Depth	0.326
Depth	0.049	Surface temperature, range	0.043
Bottom salinity, range	0.045	Summer chlorophyll, minimum	0.030
Surface current, minimum	0.019	Summer chlorophyll, range	0.028
		Surface temperature, minimum	0.003

Table 17. Variable importance results, *G. barretti*, EC study area.

Random Forest		MaxEnt	
**Predictor**	**Delta-AUC**	**Predictor**	**Delta-AUC**
Depth	0.068	Depth	0.172
Shear, maximum	0.040	Bottom salinity, range	0.082
Surface temperature, range	0.035	Surface temperature, minimum	0.032
Winter chlorophyll, range	0.029	Surface current, minimum	0.028
Spring chlorophyll, range	0.016	Surface temperature, range	0.017

Table 18. Variable importance results, *G. phlegraei*, EC study area.

Random Forest		MaxEnt	
**Predictor**	**Delta-AUC**	**Predictor**	**Delta-AUC**
Bottom salinity, range	0.161	Bottom salinity, range	0.205
Surface temperature, minimum	0.060	Depth	0.064
Summer chlorophyll, minimum	0.059	Surface current, minimum	0.009
Bottom salinity, maximum	0.023	Surface temperature, minimum	0.008
		Shear, minimum	0.001

SDM-Based Distribution Maps

Distribution maps produced using the RF models trained on the predictor sets remaining after variable elimination are considered the best available description of the spatial distribution of each response variable for the EC study area. The maps of sponge ground distribution are shown in Figure 12 and Figure 13, the map of *Geodia* spp. distribution is shown in Figure 5, and the maps of *G. barretti* and *G. phlegraei* distributions are shown in Figure 15 and Figure 16 below.

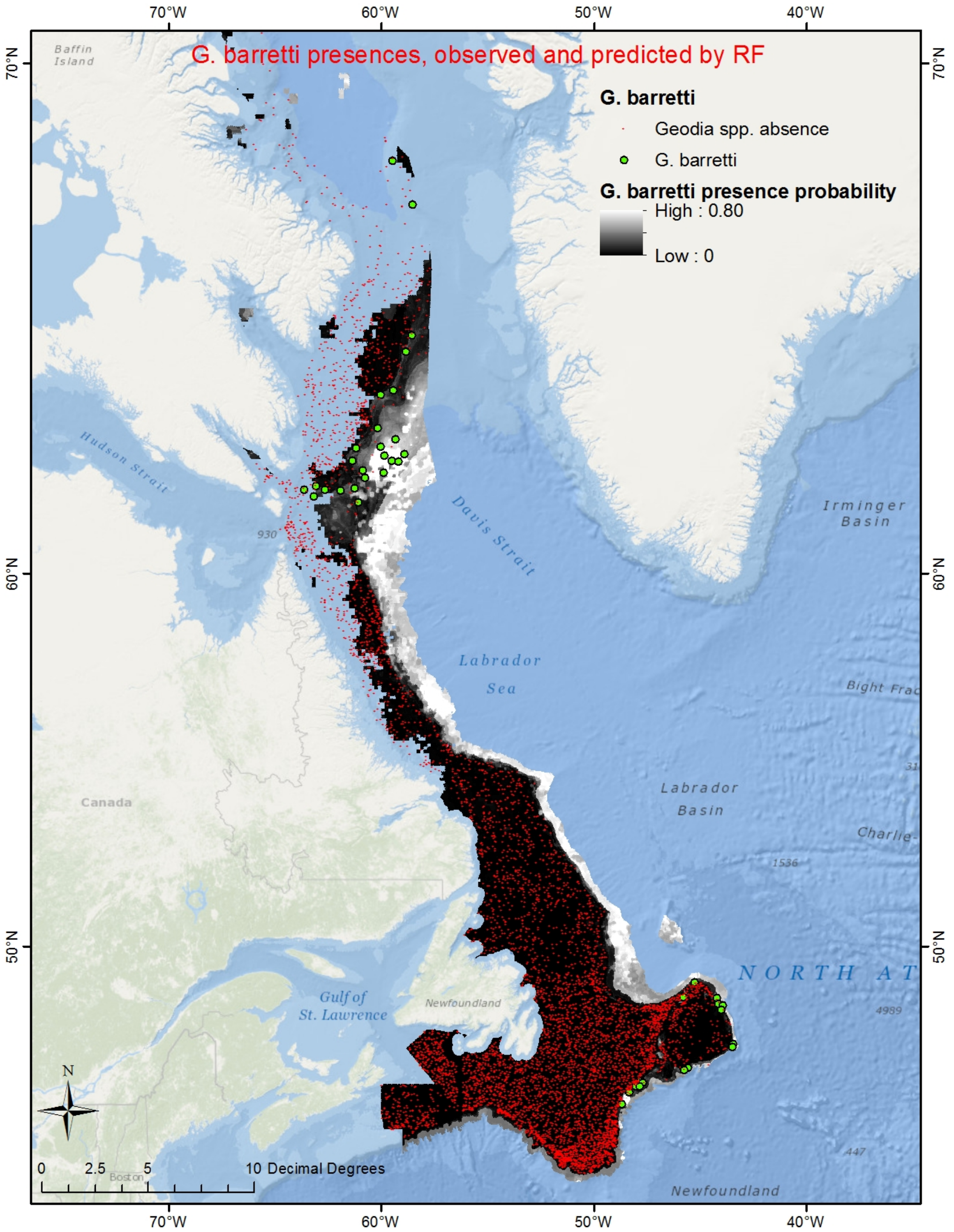

Figure 15. *G. barretti* locations in the EC study area, observed (green circles) and predicted probability by the RF model (greyscale).

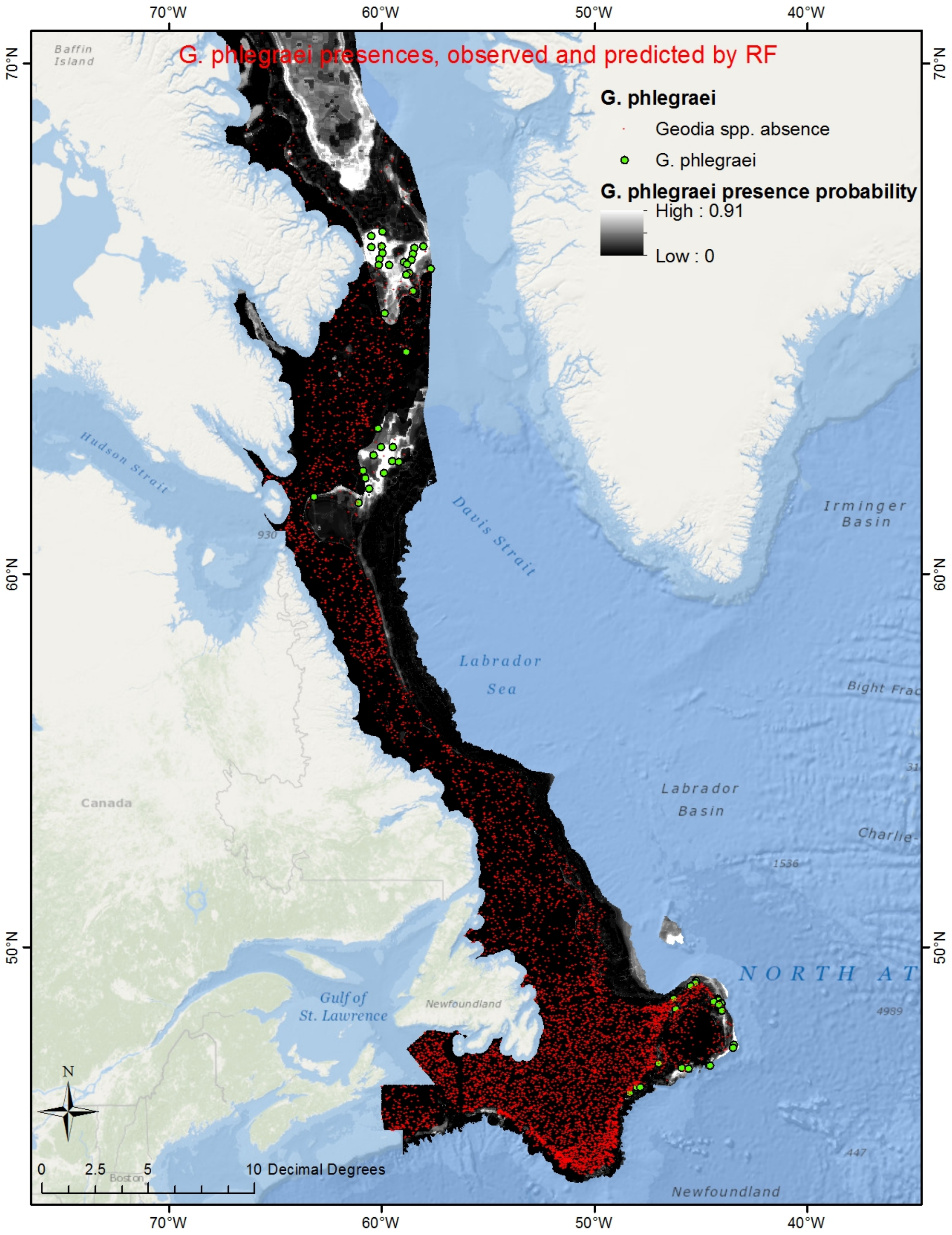

Figure 16. *G. phlegraei* locations in the EC study area, observed (green circles) and predicted probability by the RF model (greyscale).

CONCLUSION

Our study has demonstrated that species distribution models, trained using research trawl data and spatial information on depth, slope, chlorophyll-a concentration, shear, and surface and bottom temperature, salinity and current speed, are able to make excellent predictions of the distributions of *Geodia* spp. sponges and sponge grounds in the northwest Atlantic. The mapped predictions can thus be considered a good estimation of the actual distribution of sponge grounds, as defined by the global and local gear-specific thresholds used in this paper, and *Geodia* spp. presences in the study area.

Of the two model types tested, Random Forest generally out-performs MaxEnt when tested against independent validation data; the difference between the two model types is larger when models trained for the Flemish Cap are extrapolated to the East Coast study area. The two model types not only have different fits, they also make very different predictions. In deeper waters, Random Forest generally predicts high probability of presence for both sponge grounds and *Geodia* spp. sponges, while MaxEnt does not. This difference is attributable to the background samples used in MaxEnt model training, which are not used in the Random Forest model. Knowledge of which model is correct in its predictions for these deep areas can only be obtained through additional field sampling although studies to date (Murillo et al. 2012; Beazley et al. 2013) support the RF results. Unsampled areas predicted to have high presence probabilities can serve as target areas for future sampling to further validate the models. Comparing both with the kernel analyses previously employed, there is a general distribution match between the RF and kernel maps (Figure 38). However the RF model predictions have a more refined distribution as would be expected given its use of absence data in addition to the presence data. For management and conservation purposes, RF can be used to refine kernel models of the location of sponge grounds and to predict sponge ground areas where data are either sparse or non-existent.

The two approaches used for variable elimination, based on removal of collinear variables and variables not contributing to model fit, respectively, both succeeded in reducing the number of predictors from the original 50 and making the fitted models more interpretable without substantial reduction in model fit when tested on independent validation data from the area for which the models were trained. However, when training models for the Flemish Cap and applying them to the East Coast, the models trained on reduced sets of predictors had poorer fits for *Geodia* spp., *G. barretti* and *G. phlegraei*, while the models trained on the set of predictors remaining after removal of highly collinear predictors obtained the best fits for sponge grounds. If models are intended for extrapolation, we conclude that AUC-based variable elimination should not be used, while the benefit of removal of collinear predictors will depend on the response variable.

Model fits for sponge grounds are marginally better when sponge grounds are defined using a global threshold (we used 70 kg/tow) than when they are defined using local gear-specific thresholds; this also holds true for models extrapolated from the Flemish Cap to the East Coast study area. We conclude that the single global threshold enables the models to predict environmental conditions that allow a specific density of sponges to

develop and persist in a location (in our case the density that corresponds to 70 kg/tow), as opposed to predicting environmental conditions that allow locally significant sponge densities to develop and persist.

Fits are similar for models of *Geodia* spp., *G. barretti* and *G. phlegraei*. This suggests that the environmental requirements of the different *Geodia* species are similar, at least when quantified at the spatial resolution and with the predictors used in our study.

Future research should be directed specifically at establishing best practice guidelines for development of distribution models for extrapolation to unsampled areas, as this is one of the primary strengths of the models. In addition, investigation of partial dependence plots should be used to develop and test hypotheses to determine predictor values (or combinations of values for multiple predictors when interactions are significant) that represent physiological tolerance limits for each species. These can be validated against global data sets to provide improved insight into the biology and ecology of these sponges, which are otherwise difficult and costly to study.

REFERENCES

Amsler, M.O., Mcclintock, J.B., Amsler, C.D., Angus, R.A., Baker, B.J. 2009. An evaluation of sponge-associated amphipods from the Antarctic Peninsula. Antarctic Science 21: 579-589.

Beazley, L., Kenchington, E., Murillo, F.J., Sacau Cuadrado, M. 2013. Deep-sea sponge grounds enhance diversity and abundance of epibenthic megafauna in the northwest Atlantic. ICES Journal of Marine Science Online doi:10.1093/icesjms/fst124

BODC. GEBCO Gridded Global Bathymetry Data. 2009. http://www.gebco.net/.

Breiman, L. 2001. Random Forests. Machine Learning. 45: 5-32.

Breiman, L., Friedman, J., Stone, C.J., Olshen, R.A. 1984. Classification and Regression Trees. Chapman & Hall/CRC: Boca Raton, FL, USA. 358 p.

Cárdenas, P., Rapp, H.T., Klitgaard, A.B., Best, M., Thollesson, M., Tendal, O.S. 2013. Taxonomy, biogeography and DNA barcodes of *Geodia* species (Porifera, Demospongiae, Tetractinellida) in the Atlantic boreo-arctic region. Zoological Journal of the Linnean Society, 169: 251–311.

Davies, A.J., Guinotte, J.M. 2011. Global habitat suitability for framework-forming cold-water corals. PLoS ONE 6(4): e18483. doi:10.1371/journal.pone.0018483

Davies, A.J., Wisshak, M., Orr, J.C., Roberts, J.M. 2008. Predicting suitable habitat for the cold-water coral *Lophelia Pertusa* (Scleractinia). Deep-Sea Res Part I Oceanographic Research Papers. 55: 1048-1062.

de Goeij, J.M., van Duyl, F.C. 2007. Coral cavities are sinks of dissolved organic carbon (DOC). Limnology and Oceanography 52: 2608-2617.

DFO. 2009. Development of a Framework and Principles for the Biogeographic Classification of Canadian Marine Areas. Canadian Science Advisory Secretariat Science Advisory Report. 056: 1-17.

Dolan, M.F.J., Grehan, A.J., Guinan, J.C., Brown, C. 2008. Modeling the local distribution of cold-water corals in relation to bathymetric variables: Adding spatial context to deep-sea video data. Deep-Sea Research Part I-Oceanographic Research Papers. 55: 1564-1579.

Elith, J., Graham, C., Anderson, R., Dudik, M., Ferrier, S., Guisan, A., Hijmans, R., Huettmann, F., Leathwick, J., Lehmann, A., Li, J., Lohmann, L.G., Loiselle, B.A., Manion, G., Moritz, C., Nakamura, M., Nakazawa, Y., Overton, J. McC. M., Peterson, A.T., Phillips, S.J., Richardson, K., Scachetti-Pereira, R., Schapire, R.E., Soberon, J., Williams, S., Wisz, M.S., Zimmermann, N.E. 2006. Novel methods improve prediction of species' distributions from occurrence data. Ecography 29:129-151.

EU. MyOcean. http://www.myocean.eu/web/24-catalogue.php. (accessed on 27 March, 2013).

Fawcett, T. 2006. An Introduction to ROC Analysis. Pattern Recognition Letters 27: 861-874.

Ferry, N., Parent, L., Garric, G., Bricaud, C., Testut, C., Le Galloudec, O., Lellouche, J., Drévillon, M., Greiner, E., Barnier, B., Molines, J-M., Jourdain, N., Guinehut, S., Cabanes, C., Zawadzki, L. 2012. GLORYS2V1 Global Ocean Reanalysis of the Altimetric Era (1993-2009) at Meso Scale. Mercator Ocean Newsletter 44: 28-39.

Fuller, S.D. 2011. Diversity of marine sponges in the Northwest Atlantic. PhD thesis, Dalhousie University, Halifax. 215 pp.

Franklin, J. 2009. Mapping Species Distributions: Spatial Inference and Prediction. Cambridge University Press, Cambridge, UK. 338 pp.

Graham, M. 2003. Confronting multicollinearity in ecological multiple regression. Ecology 84: 2809-2815.

Guinan, J., Grehan, A.J., Dolan, M.F.J., Brown, C. 2009. Quantifying relationships between video observations of cold-water coral cover and seafloor features in Rockall Trough, West of Ireland. Marine Ecology Progress Series 375: 125-138.

Hentschel, U., Hopke, J., Horn, M., Friedrich, A., Wagner, M., Hacker, J., Moore, B. 2002. Molecular evidence for a uniform microbial community in sponges from different oceans. Applications in Environmental Microbiology 68: 4431-4440.

Herrnkind, W., Butler, M., Hunt, J., Childress, M. 1997. Role of physical refugia: Implications from a mass sponge die-off in a lobster nursery in Florida. Marine and Freshwater Research 48: 759-769.

Hijmans, R.J., Phillips, S., Leathwick, J., Elith, J. 2011. Package 'Dismo' for Species Distribution Modeling with R. http://cran.r-project.org/web/packages/dismo/dismo.pdf

Hill, R.T. 2004. Microbes from marine sponges: A treasure trove of biodiversity for natural products discovery. In Microbial Diversity and Bioprospecting. Edited by Ball, A.T. ASM Press, Washington DC, USA.

Hogg, M.M., Tendall, O.S., Conway, K.W., Pomponi, S.A., van Soest, R.W.M., Gutt, J., Krautter, M., Roberts, J.M. 2010. Deep-sea Sponge Grounds: Reservoirs of Biodiversity. UNEP-WCMC. Cambridge, UK. 84 p.

Hooper, J.N.A., van Soest, R.W.M., Editors. 2002. Systema Porifera: A Guide to the Classification of Sponges, 1st ed. Kluwer Academic/Plenum. Dordrecht, The Netherlands. 1756 p.

ICES. 2009. Report of the ICES-NAFO Working Group on Deep-Water Ecology (WGDEC), 9 13 March 2009. ICES Document CM 2009/ACOM: 23, 92 pp

Japkowicz, N., Stephen, S. 2002. The class imbalance problem: A systematic study. Intelligent Data Analysis 6: 429-449.

Kenchington, E., Power, D., Koen-Alonso, K. 2013. Associations of demersal fish with sponge grounds on the continental slopes of the northwest Atlantic. Marine Ecology Progress Series 477: 217–230.

Kenchington, E., Siferd, T., Lirette, C. 2012. Arctic marine biodiversity: Indicators for monitoring coral and sponge megafauna in the eastern Arctic. DFO Canadian Scientific Advisory Secretariat Research Document 2012/003: vi + 44p.

Kenchington, E., Lirette, C., Cogswell, A., Archambault, D., Archambault, P., Benoit, H., Bernier, D., Brodie, B., Fuller, S., Gilkinson, K., Lévesque, M., Power, D., Siferd, T., Treble, M., Wareham, V. 2010. Delineating coral and sponge concentrations in the biogeographic regions of the east coast of Canada using spatial analyses. Canadian Science Advisory Secretariat. 041: i-vi + 202 pages.

Kenchington, E., Cogswell, A., Lirette, C., Murillo-Perez, F.J. 2009. The use of density analyses to delineate sponge grounds and other benthic VMEs from trawl survey data. Northwest Atlantic Fisheries Organization Scientific Council Report 09/6: 1-15.

Klitgaard, A. 1995. The fauna associated with outer shelf and upper slope sponges (Porifera, Demospongiae) at the Faroe Islands, northeastern Atlantic. Sarsia 80: 1-22.

Klitgaard, A., Tendal, O. 2004. Distribution and species composition of mass occurrences of large-sized sponges in the northeast Atlantic. Progress in Oceanography 61: 57-98.

Klitgaard, A., Tendal, O., Westerberg, H. 1997. Mass occurrences of large sponges (Porifera) in Faroe Island (NE Atlantic) shelf and slope areas: Characteristics, distribution and possible causes. In: Hawings, L.E., Hutchinson, S., with Jensen, A.C., Sheader, M., Williams, J.A., editors. The Responses of Marine Organisms to their Environments. University of Southampton, UK: Proceedings of the 30th European Marine Biological Symposium, p 129-42.

Knudby, A., Brenning, A., LeDrew, E. 2010. New approaches to modeling fish-habitat relationships. Ecological Modeling 221: 503-511.

Knudby, A., LeDrew, E., Brenning, A. 2010. Predictive mapping of reef fish species richness, diversity and biomass in Zanzibar using IKONOS Imagery and Machine-Learning Techniques. Remote Sensing Environment 114: 1230-1241.

Lawler, J.J., Shafer, S.L., White, D., Kareiva, P., Maurer, E.P., Blaustein, A.R., Bartlein, P.J. 2009. Projected climate-induced faunal change in the Western Hemisphere. Ecology 90: 588-597.

Legendre, P., Legendre, L. 2012. Numerical Ecology, 3rd ed. Elsevier. Amsterdam, The Netherlands. 1006 p.

Leys, S., Wilson, K., Holeton, C., Reiswig, H., Austin, W., Tunnicliffe, V. 2004. Patterns of glass sponge (Porifera, Hexactinellida) distribution in coastal waters of British Columbia, Canada. Marine Ecology Progress Series 283: 133-149.

Liaw, A., Wiener, M. 2002. Classification and Regression by Random Forest. R News 2: 18-22.

Lutz, M.J., Caldeira, K., Dunbar, R.B., Behrenfeld, M.J. 2007. Seasonal rhythms of net primary production and particulate organic carbon flux to depth describe the efficiency of biological pump in the global ocean. Journal of Geophysical Research-Oceans 112: C10011.

Müller, W.E.G., Schröder, H.C., Wiens, M., Perovi-Ottstadt, S., Batel, R., Müller, I.M. 2004. Traditional and modern biomedical prospecting: Part II - the benefits: approaches for a sustainable exploitation of biodiversity (secondary metabolites and biomaterials from sponges). Evidenced Based Complementary and Alternative Medicine 1(2): 83-88.

Murillo, F.J., Duran Munoz, P., Cristobo, J., Rios, P., Gonzalez, C., Kenchington, E., Serrano, A. 2012. Deep-Sea sponge grounds of the Flemish Cap, Flemish Pass and the Grand Banks of Newfoundland (Northwest Atlantic Ocean): Distribution and species composition. Marine Biology Research 8: 842-854.

NASA. OceanColor. http://oceancolor.gsfc.nasa.gov/. (accessed on 12 September, 2012).

NODC. World Ocean Database. http://www.nodc.noaa.gov/OC5/indprod.html. (accessed on 27 March, 2013).

Penney, A.J., Parker, S.J., Brown, J.H. 2009. Protection measures implemented by New Zealand for vulnerable marine ecosystems in the South Pacific Ocean. Marine Ecology Progress Series 397: 341-354.

Peterson, A. 2003. Predicting the geography of species' invasions via ecological niche modeling. Quarterly Review of Biology 78: 419-433.

Peterson, A., Robins, C. 2003. Using ecological-niche modeling to predict barred owl invasions with implications for spotted owl conservation. Conservation Biology 17: 1161-1165.

Phillips, S., Anderson, R., Schapire, R. 2006. Maximum entropy modeling of species geographic distributions. Ecological Modeling 190: 231-259.

R Core Development Team. 2012. R: A Language and Environment for Statistical Computing. Version 2.15.

Ross, R., Howell, K.L. 2012. Use of predictive habitat modeling to assess the distribution and extent of the current protection of 'listed' Deep-Sea Habitats. Diversity and Distributions 19(4): 433-445.

Spetland, F., Rapp, H.T., Hoffmann, F., Tendal, O.S. 2007. Sexual reproduction of *Geodia barretti* Bowerbank 1858 (Porifera, Astrophorida) in two Scandinavian fjords. In Porifera Research: Biodiversity, Innovation and Sustainability, Série Livros 28 ed. Custódio, M.R., Lôbo-Hajdu, G., Hajdu, E. and Muricy, G., Eds. Museu Nacional. Rio de Janeiro, Brazil. pp. 613-620.

Stockwell, D., Peterson, A. 2002. Effects of sample size on accuracy of species distribution models. Ecological Modeling 148: 1-13.

Tittensor, D.P., Baco, A.R., Brewin, P.E., Clark, M.R., Consalvey, M., Hall-Spencer, J., Rowden, A.A., Schlacher, T., Stocks, K.I., Rogers, A.D. 2009. Predicting global habitat suitability for stony corals on seamounts. Journal of Biogeography 36: 1111-1128.

UNGA. 2007. Sustainable Fisheries, Including through the 1995 Agreement for the Implementation of the Provisions of the United Nations Convention on the Law of the Sea of 10 December 1982 Relating to the Conservation and Management of Straddling Fish Stocks and Highly Migratory Fish Stocks, and Related Instruments. 2007. Resolution 61/105.

van Hulse, J., Khoshgoftaar, T. 2009. Knowledge discovery from imbalanced and noisy data. Data and Knowledge Engineering 68: 1513-1542.

van Soest, R.W.M., Boury-Esnault, N., Hooper, J.N.A., Rützler, K., de Voogd, N.J., Alvarez, B., Hajdu, E., Pisera, A.B., Vacelet, J., Manconi, R., Schoenberg, C., Janussen, D., Tabachnick, K.R., Klautau, M., Picton, B., Kelly, M., Vacelet, J., Dohrmann, M., Cristina Diaz, M. 2013. World Porifera Database. http://www.marinespecies.org/porifera.

Yesson, C., Taylor, M.L., Tittensor, D.P., Davies, A.J., Guinotte, J., Baco, A., Black, J., Hall-Spencer, J.M., Rogers, A.D. 2012. Global habitat suitability of cold-water octocorals. Journal of Biogeography 39: 1278–1292.

APPENDIX

This appendix contains all 1) partial dependence plots, 2) variable importance results, and 3) mapped predictions of distributions for the FC study area.

Partial Dependence Plots

FC Study Area

Geodia spp.

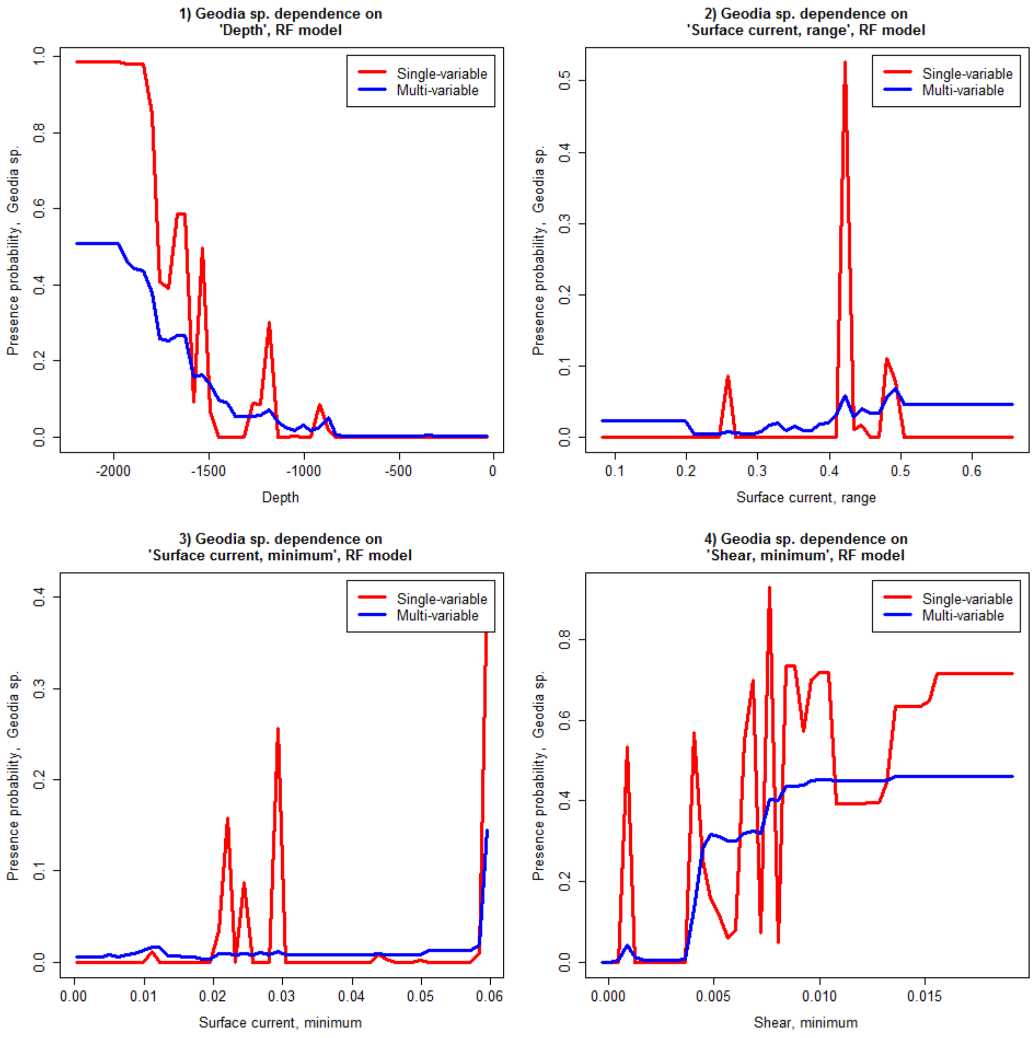

Figure 17. Partial dependence plots for *Geodia* spp., RF model, FC study area.

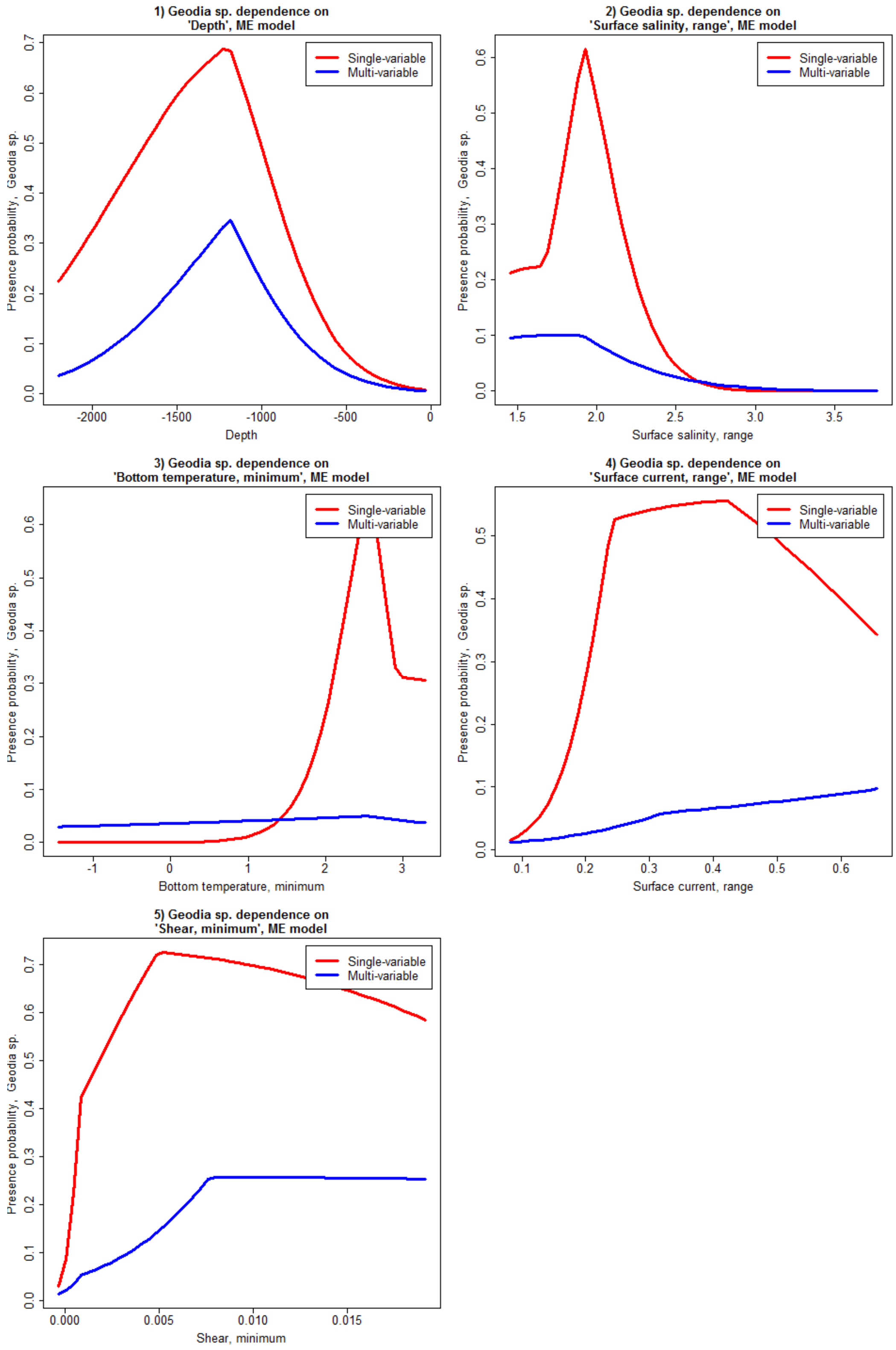

Figure 18. Partial dependence plots for *Geodia* spp., ME model, FC study area.

G. barretti

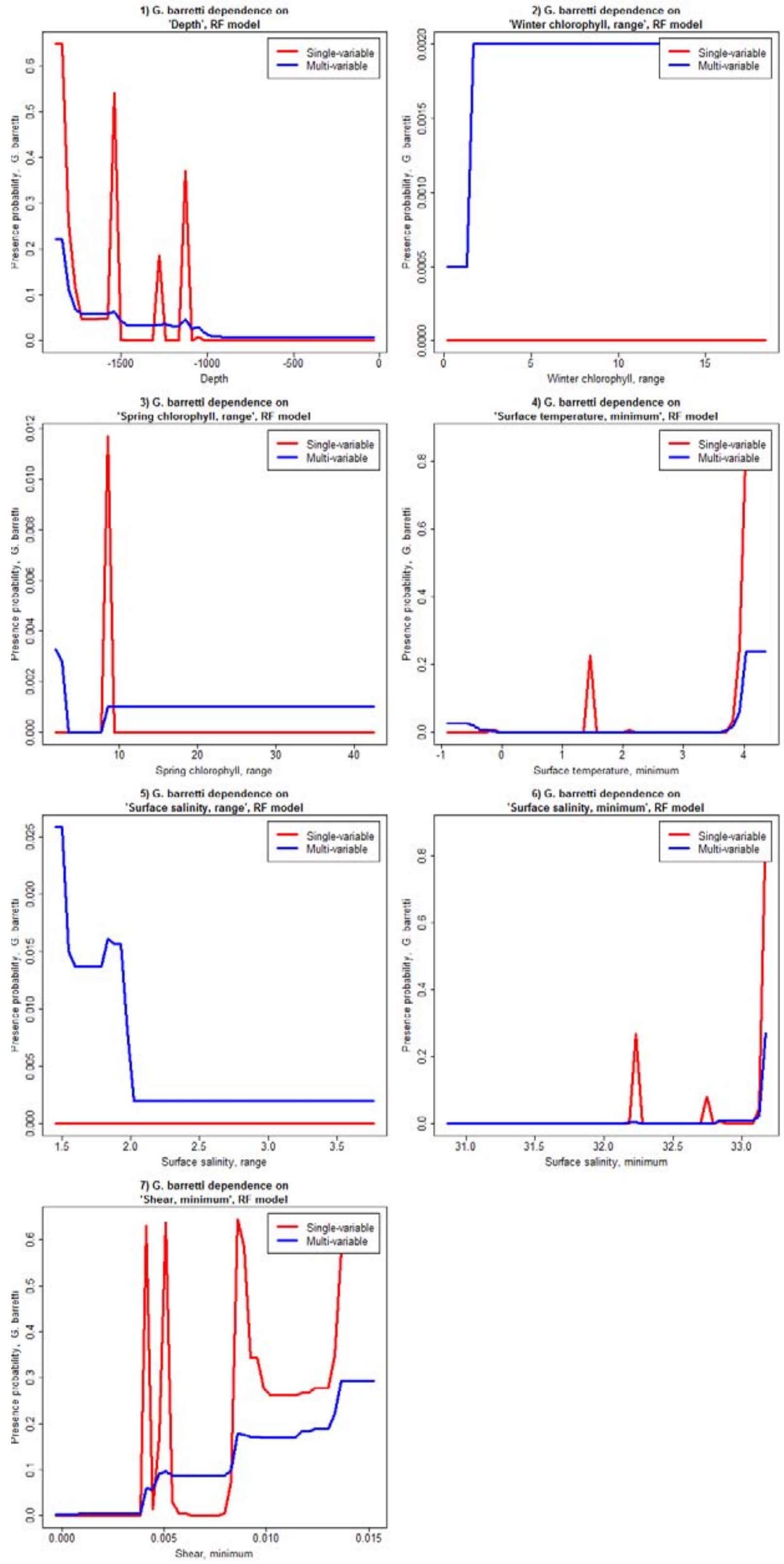

Figure 19. Partial dependence plots for *G. barretti*, RF model, FC study area.

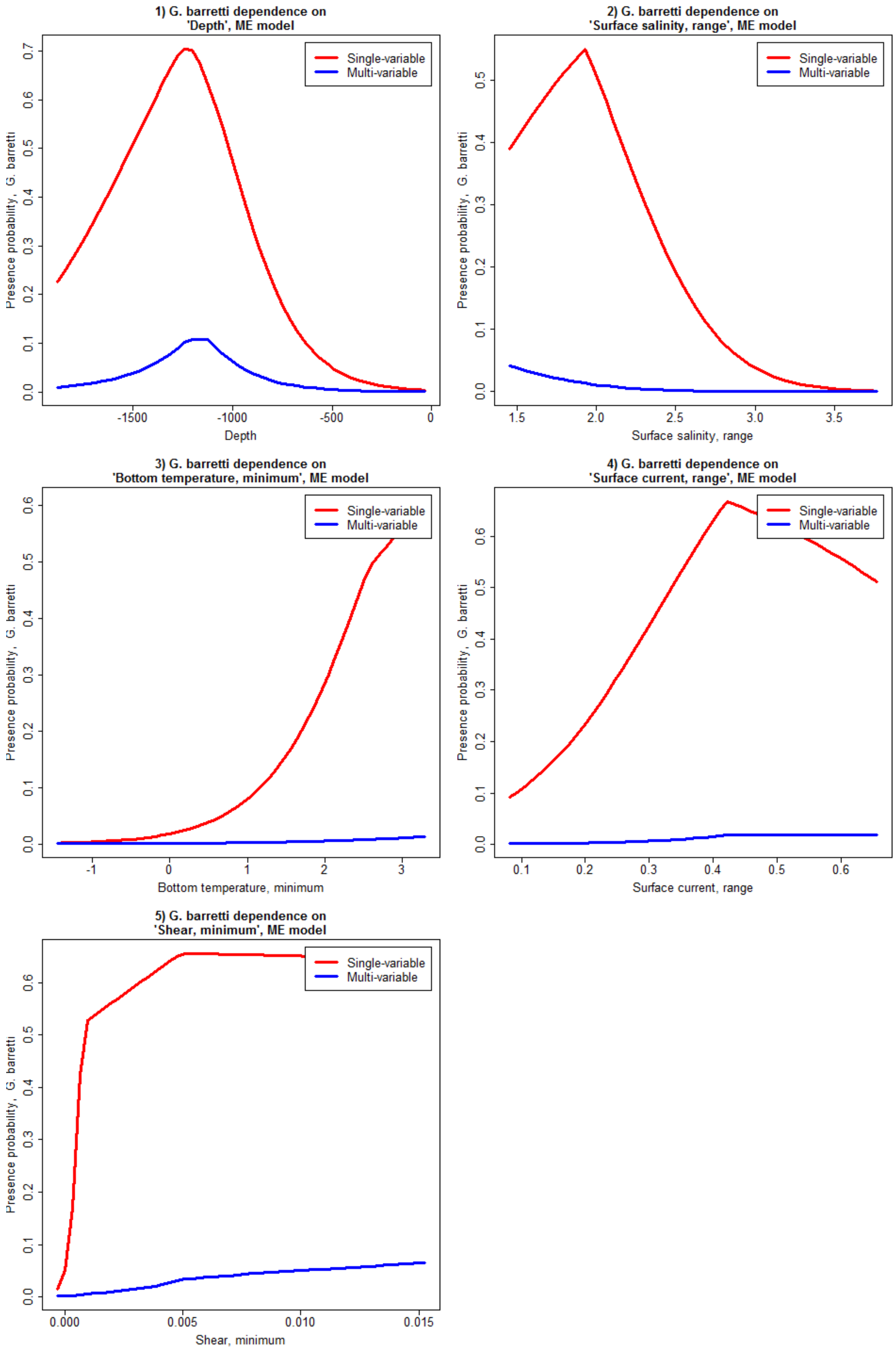

Figure 20. Partial dependence plots for *G. barretti*, ME model, FC study area.

G. phlegraei

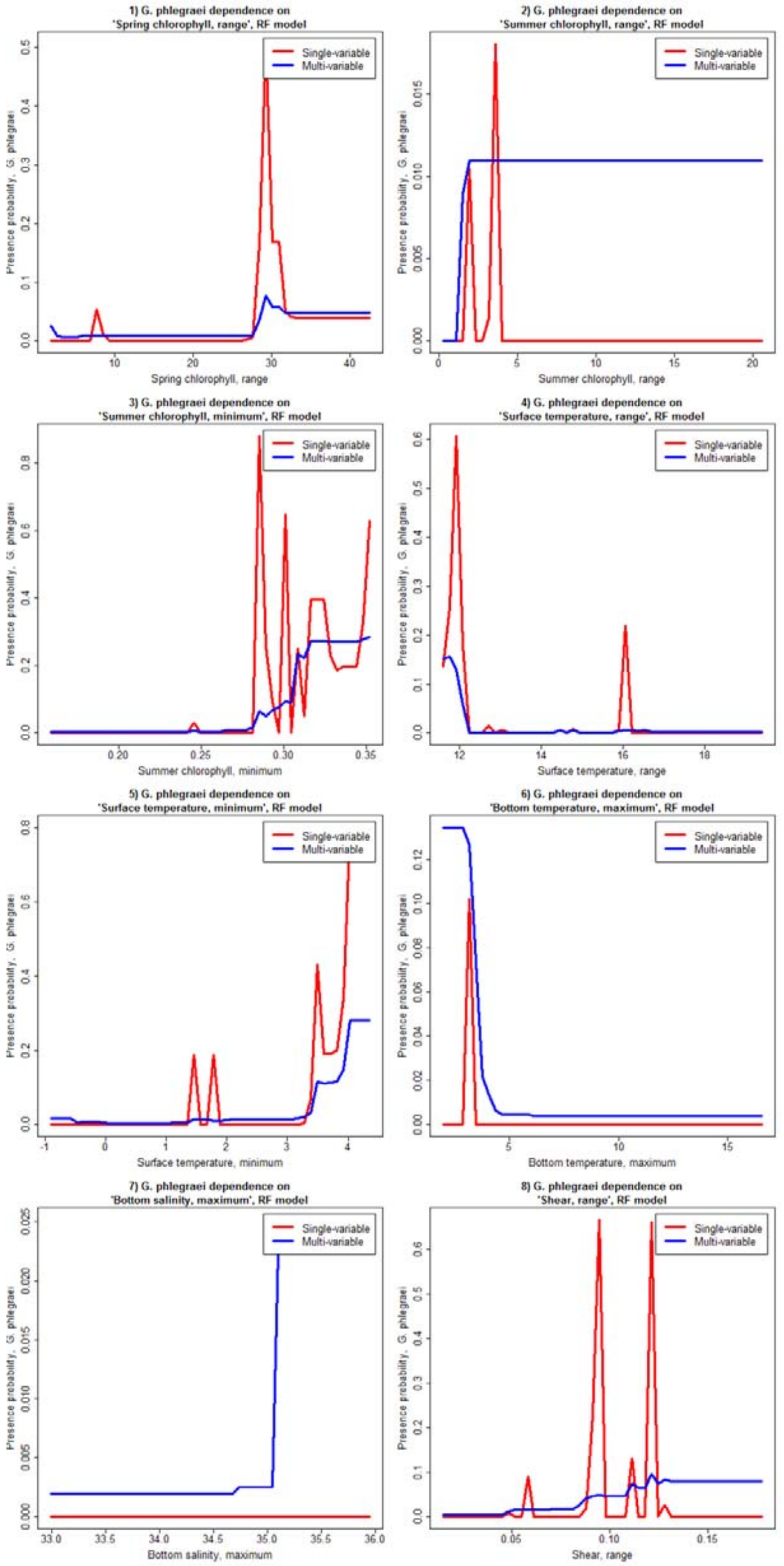

Figure 21. Partial dependence plots for *G. phlegraei*, RF model, FC study area.

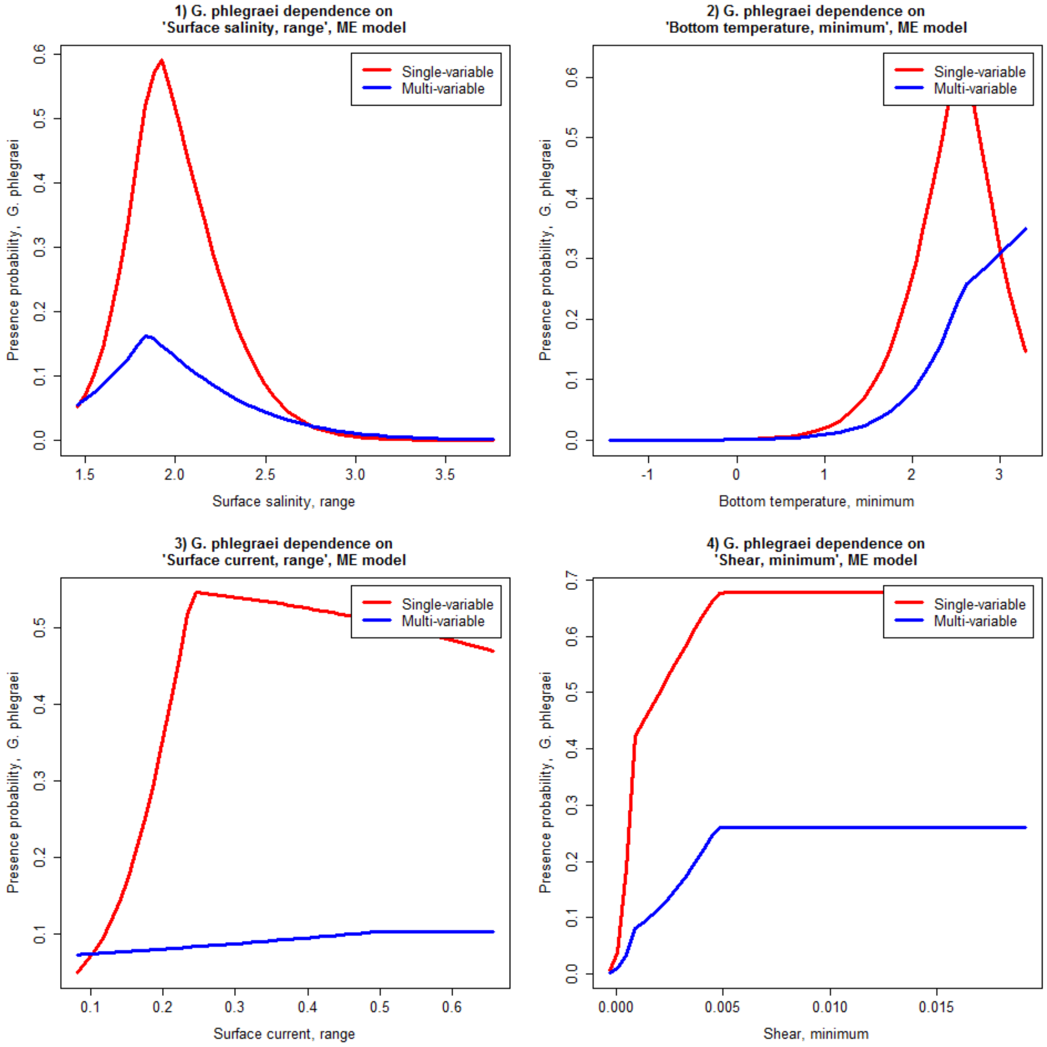

Figure 22. Partial dependence plots for *G. phlegraei*, ME model, FC study area.

Sponge Grounds

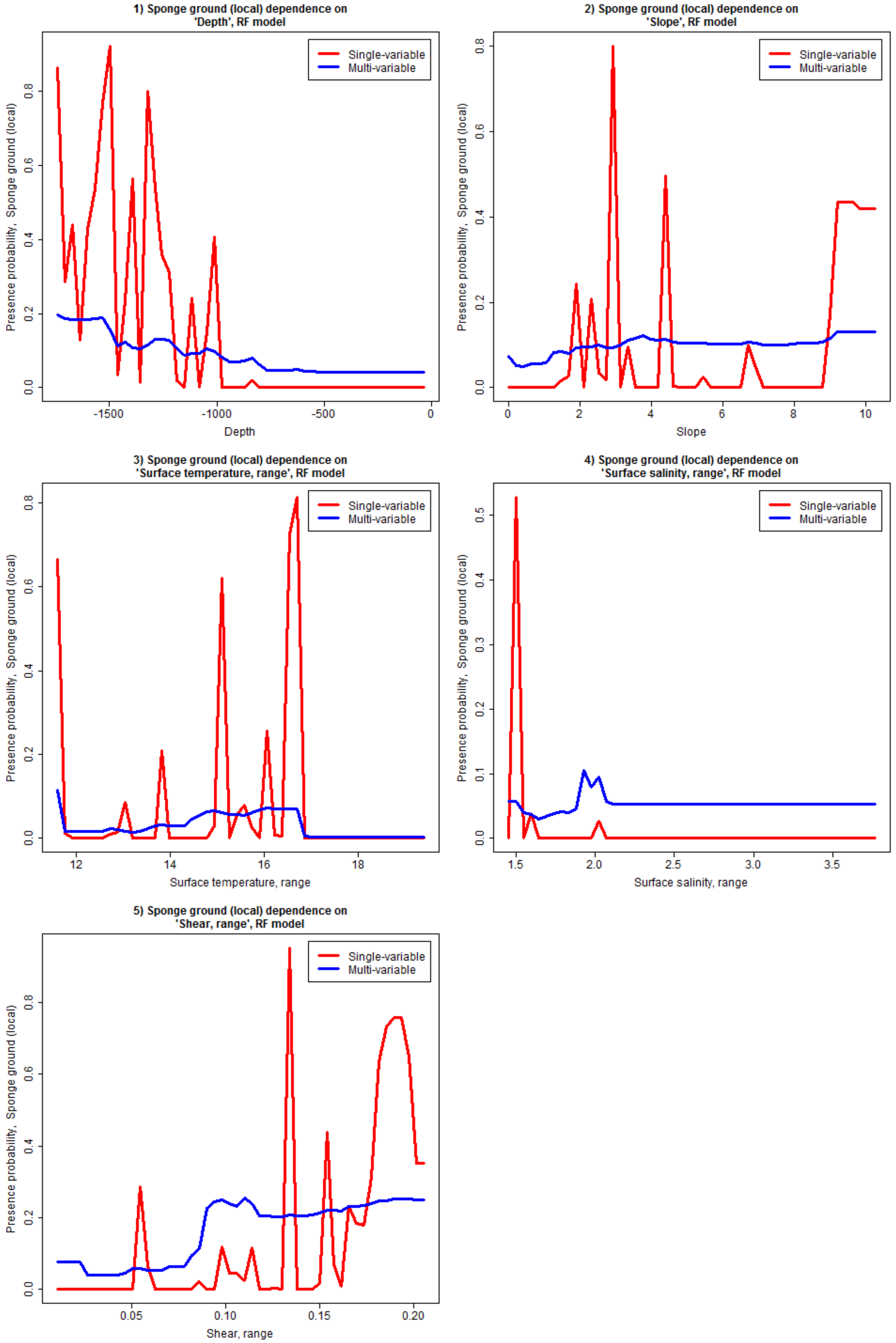

Figure 23. Partial dependence plots for sponge grounds, RF model, FC study area.

EC Study Area

Sponge Grounds

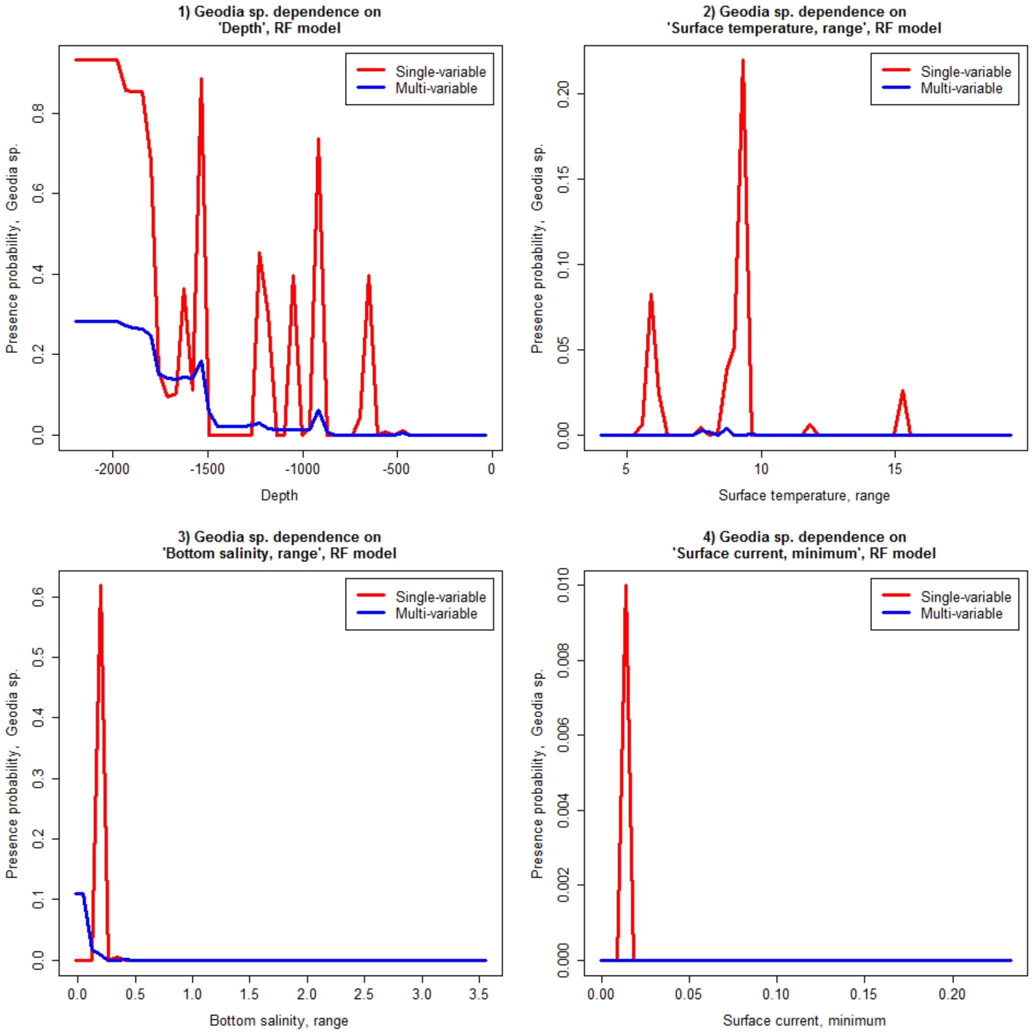

Figure 24. Partial dependence plots for *Geodia* spp., RF model, EC study area.

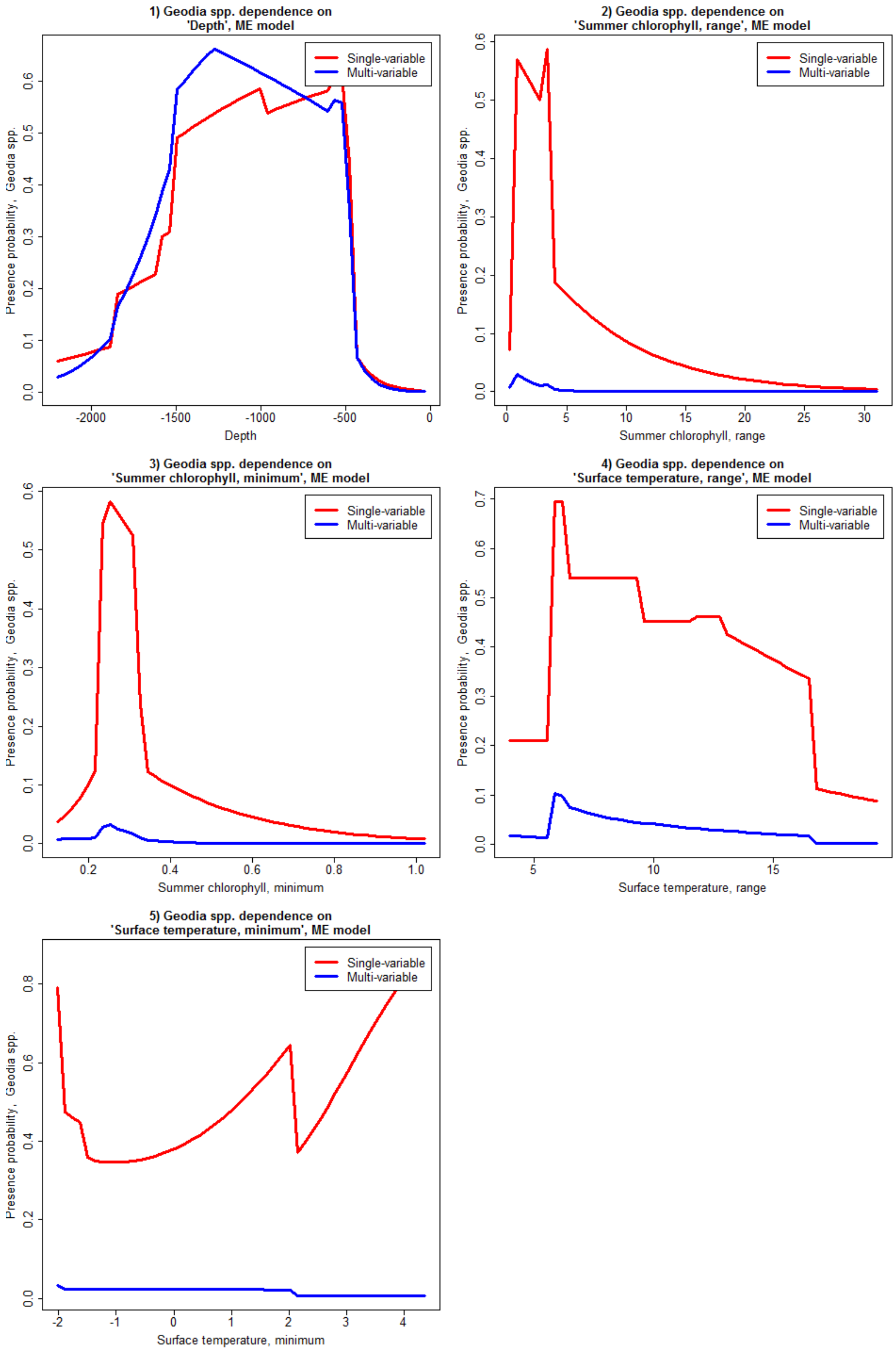

Figure 25. Partial dependence plots for *Geodia* spp., ME model, EC study area.

G. barretti

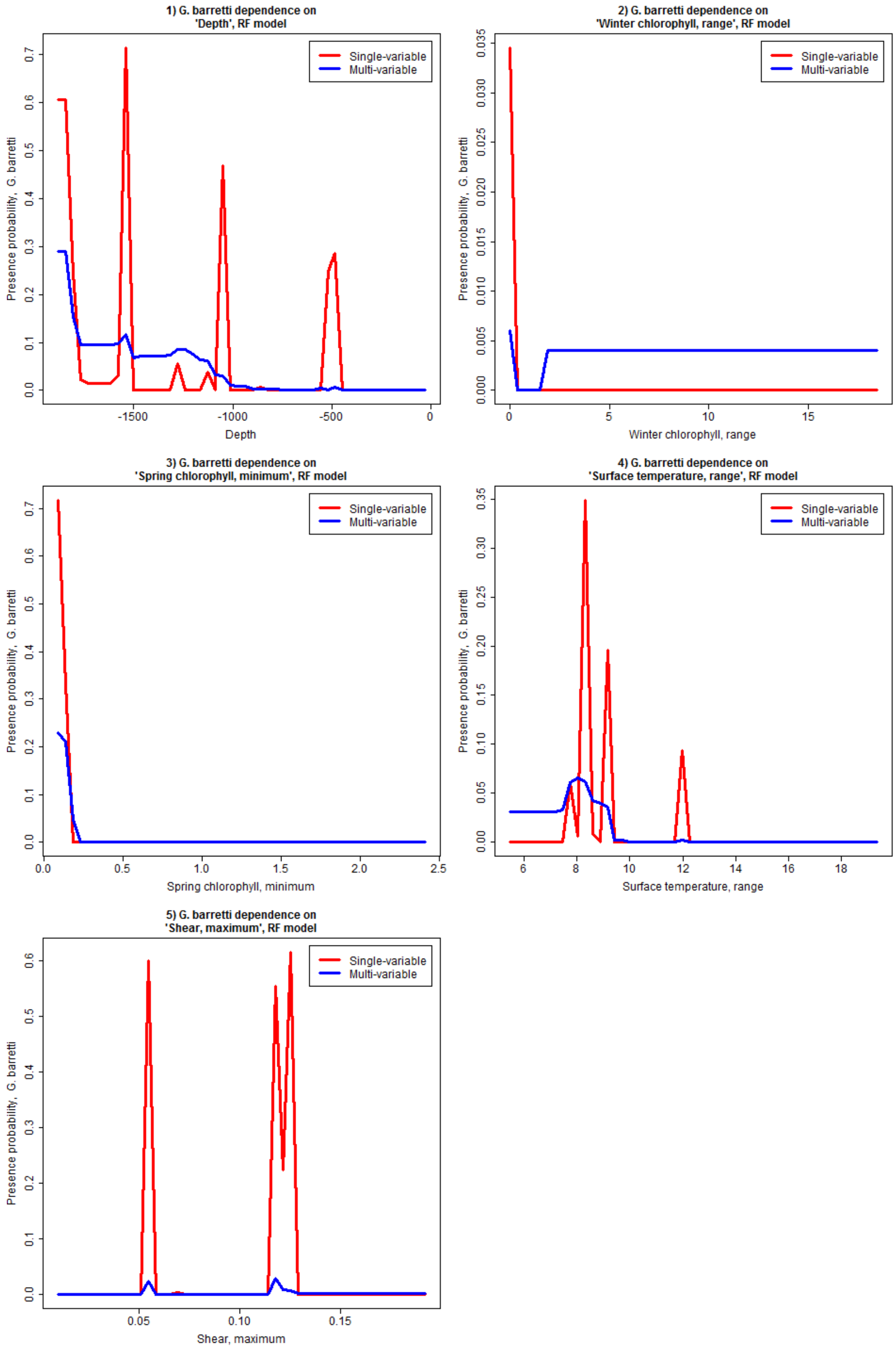

Figure 26. Partial dependence plots for *G. barretti*, RF model, EC study area.

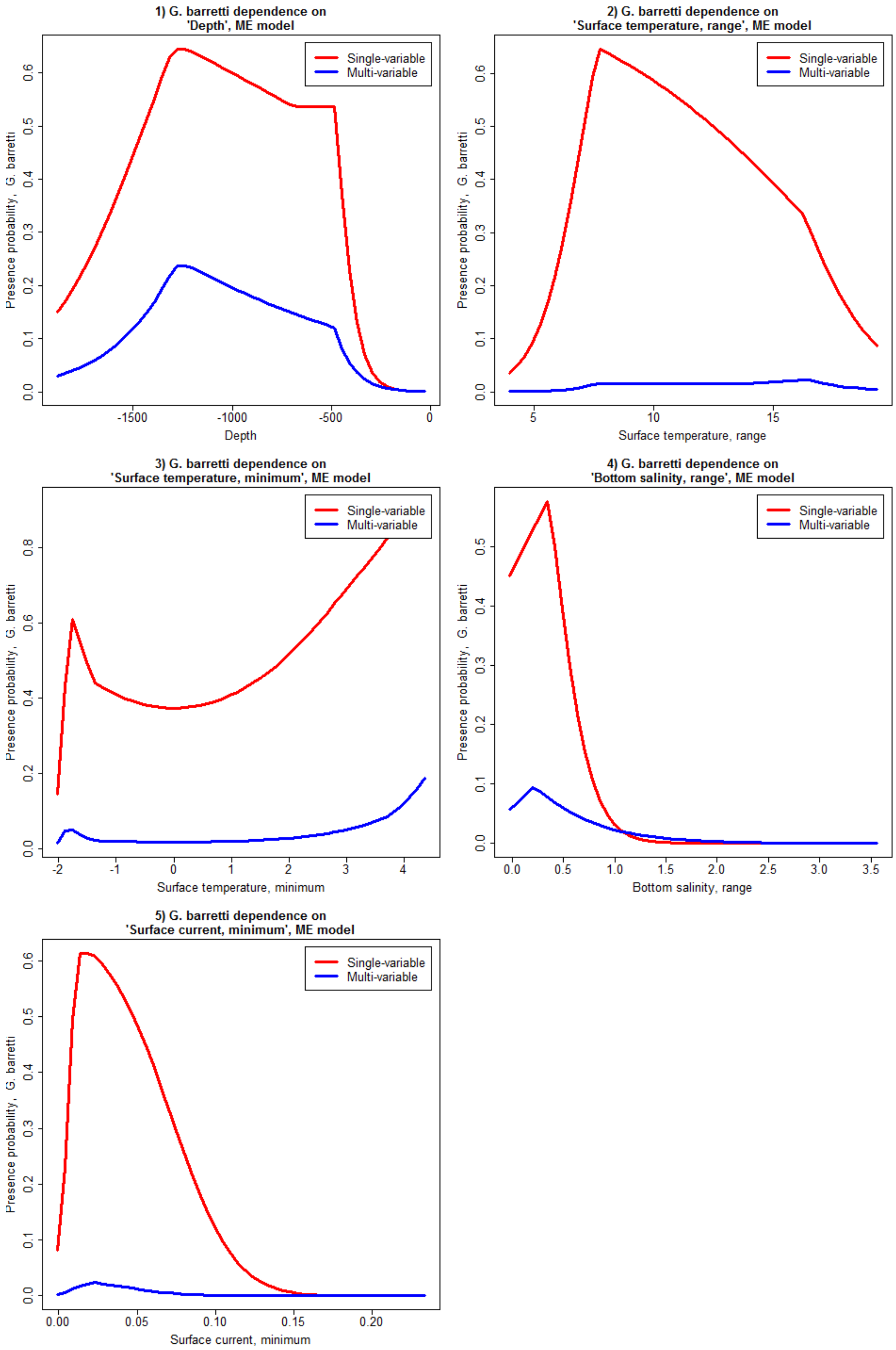

Figure 27. Partial dependence plots for *G. barretti*, ME model, EC study area.

G. phlegraei

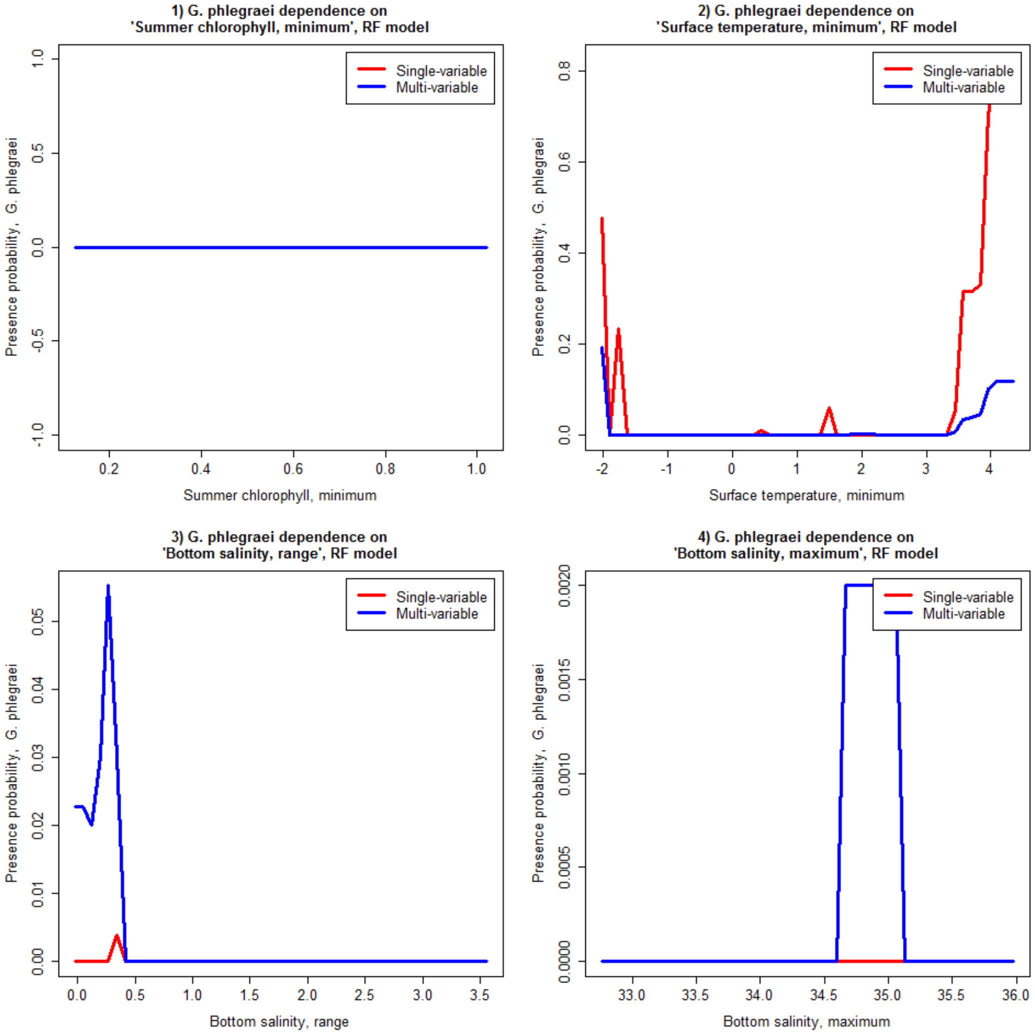

Figure 28. Partial dependence plots for *G. phlegraei*, RF model, EC study area.

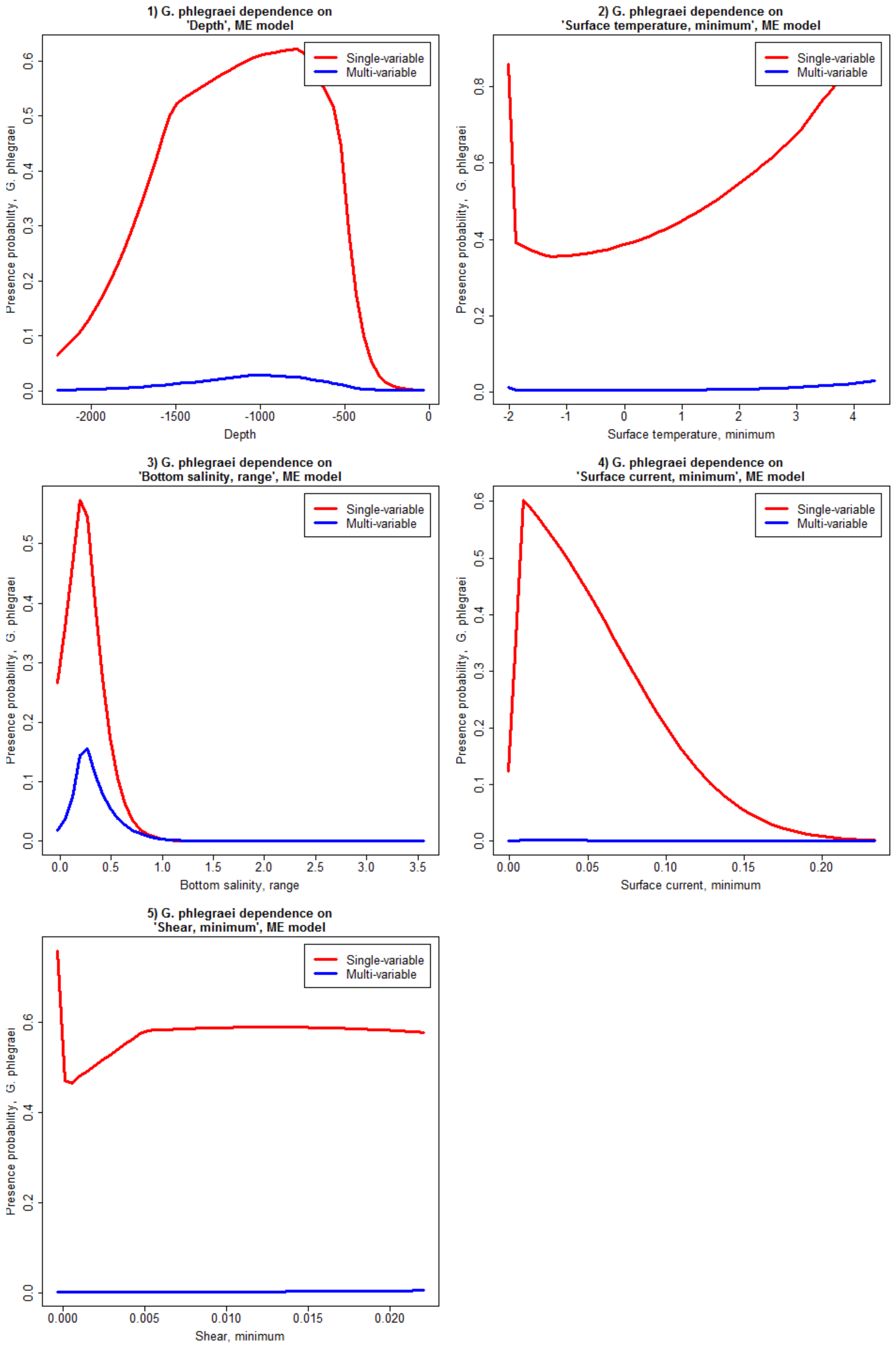

Figure 29. Partial dependence plots for *G. phlegraei*, ME model, EC study area.

Sponge Grounds (Local Gear-Specific Thresholds)

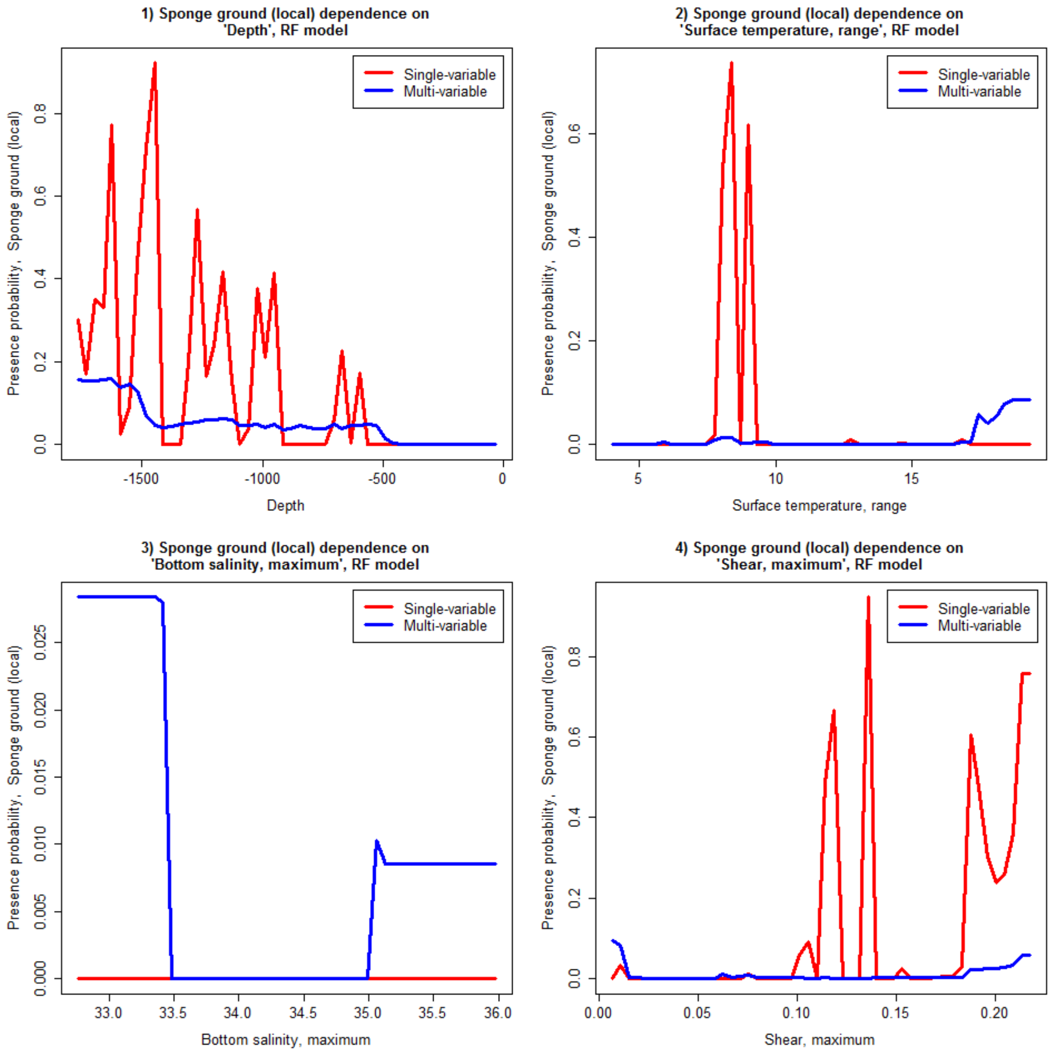

Figure 30. Partial dependence plots for sponge grounds (local gear-specific thresholds), RF model, EC study area.

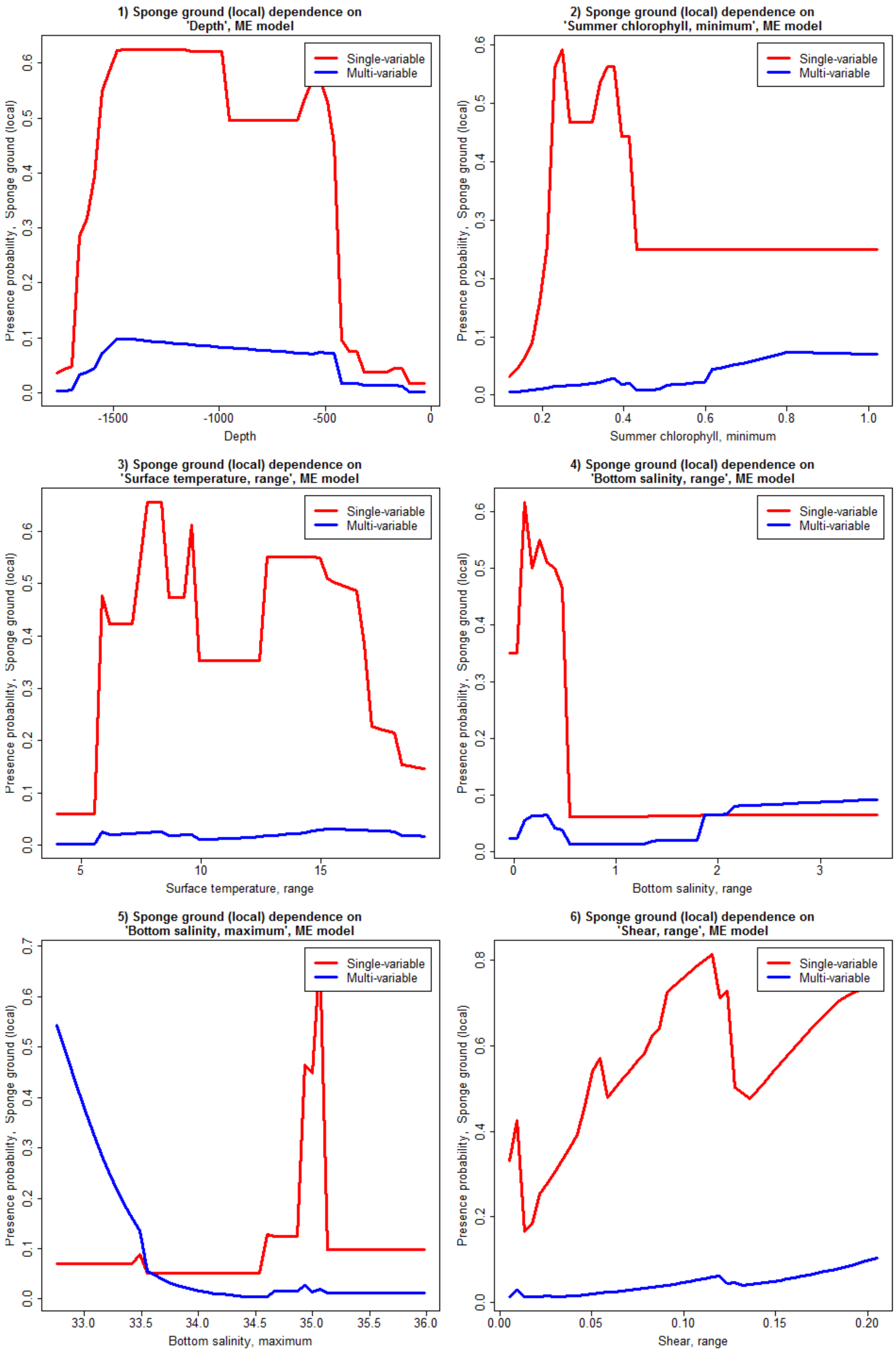

Figure 31. Partial dependence plots for sponge grounds (local gear-specific thresholds), ME model, EC study area.

Sponge Grounds (Global Thresholds)

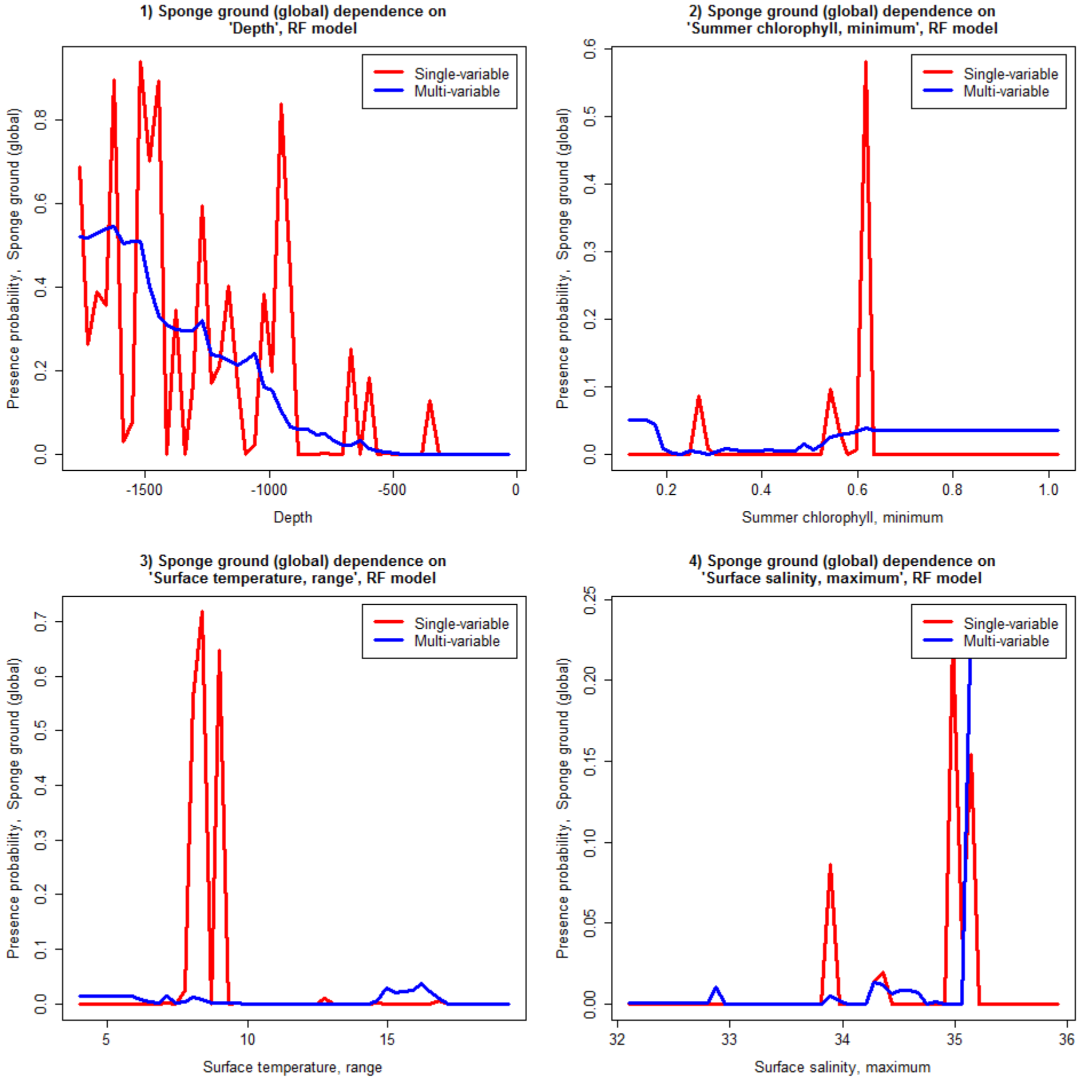

Figure 32. Partial dependence plots for sponge grounds (global threshold), RF model, EC study area.

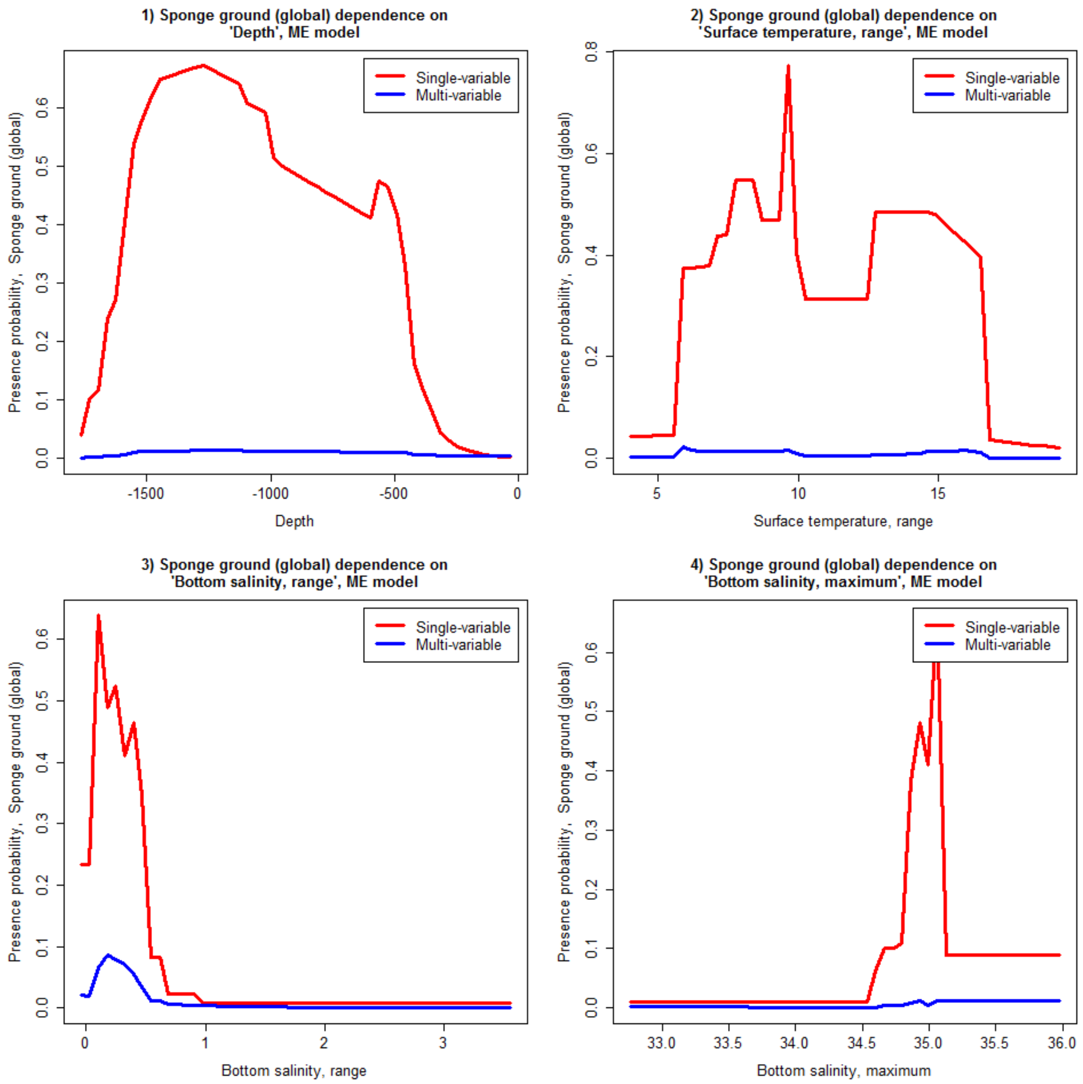

Figure 33. Partial dependence plots for sponge grounds (global threshold), ME model, EC study area.

Variable Importance Results

FC Study Area

Geodia spp.

Table 19. Variable importance results, *Geodia* spp., FC study area.

Random Forest		MaxEnt	
**Predictor**	**Delta-AUC**	**Predictor**	**Delta-AUC**
Depth	0.105	Depth	0.152
Shear, minimum	0.068	Surface salinity, range	0.091
Surface current, minimum	0.032	Shear, minimum	0.066
Surface current, range	0.017	Surface current, range	0.009
		Bottom temperature, minimum	0.002

Geodia barretti

Table 20. Variable importance results, *G. barretti*, FC study area.

Random Forest		MaxEnt	
**Predictor**	**Delta-AUC**	**Predictor**	**Delta-AUC**
Depth	0.122	Surface salinity, range	0.128
Surface salinity, minimum	0.038	Depth	0.125
Surface temperature, minimum	0.037	Bottom temperature, minimum	0.054
Shear, minimum	0.034	Surface current, range	0.050
Surface salinity, range	0.023	Shear, minimum	0.045
Winter chlorophyll, range	0.014		
Spring chlorophyll, range	0.010		

Geodia phlegraei

Table 21. Variable importance results, *G. phlegraei*, FC study area.

Random Forest		MaxEnt	
**Predictor**	**Delta-AUC**	**Predictor**	**Delta-AUC**
Summer chlorophyll, range	0.047	Shear, minimum	0.164
Bottom temperature, minimum	0.047	Bottom temperature, minimum	0.134
Shear, range	0.039	Surface salinity, range	0.098
Surface temperature, range	0.034	Surface current, range	0.000
Summer chlorophyll, minimum	0.033		
Surface temperature, minimum	0.028		
Bottom salinity, maximum	0.028		
Spring chlorophyll, range	0.003		

Sponge Grounds

Table 22. Variable importance results, sponge grounds, FC study area.

Random Forest		MaxEnt	
**Predictor**	**Delta-AUC**	**Predictor**	**Delta-AUC**
Depth	0.061	Shear, range	0.106
Shear, range	0.061	Depth	0.083
Surface salinity, range	0.024	Bottom temperature, minimum	0.053
Surface temperature, range	0.022	Surface salinity, range	0.024
Slope	0.020		

Mapped Predictions, FC Study Area

Geodia spp.

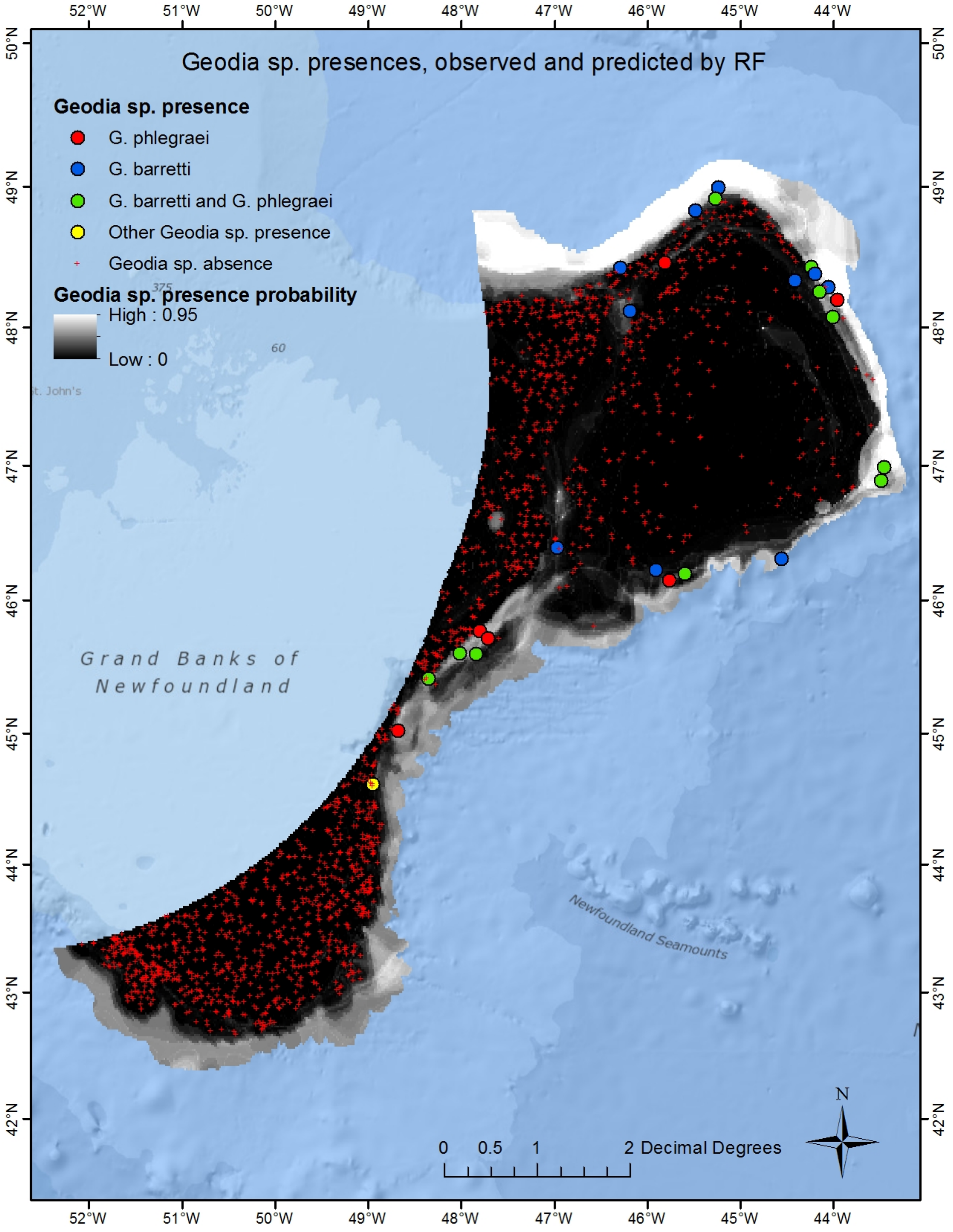

Figure 34. *Geodia* spp. locations in the FC study area, observed (coloured circles) and predicted probability by the RF model (greyscale).

G. barretti

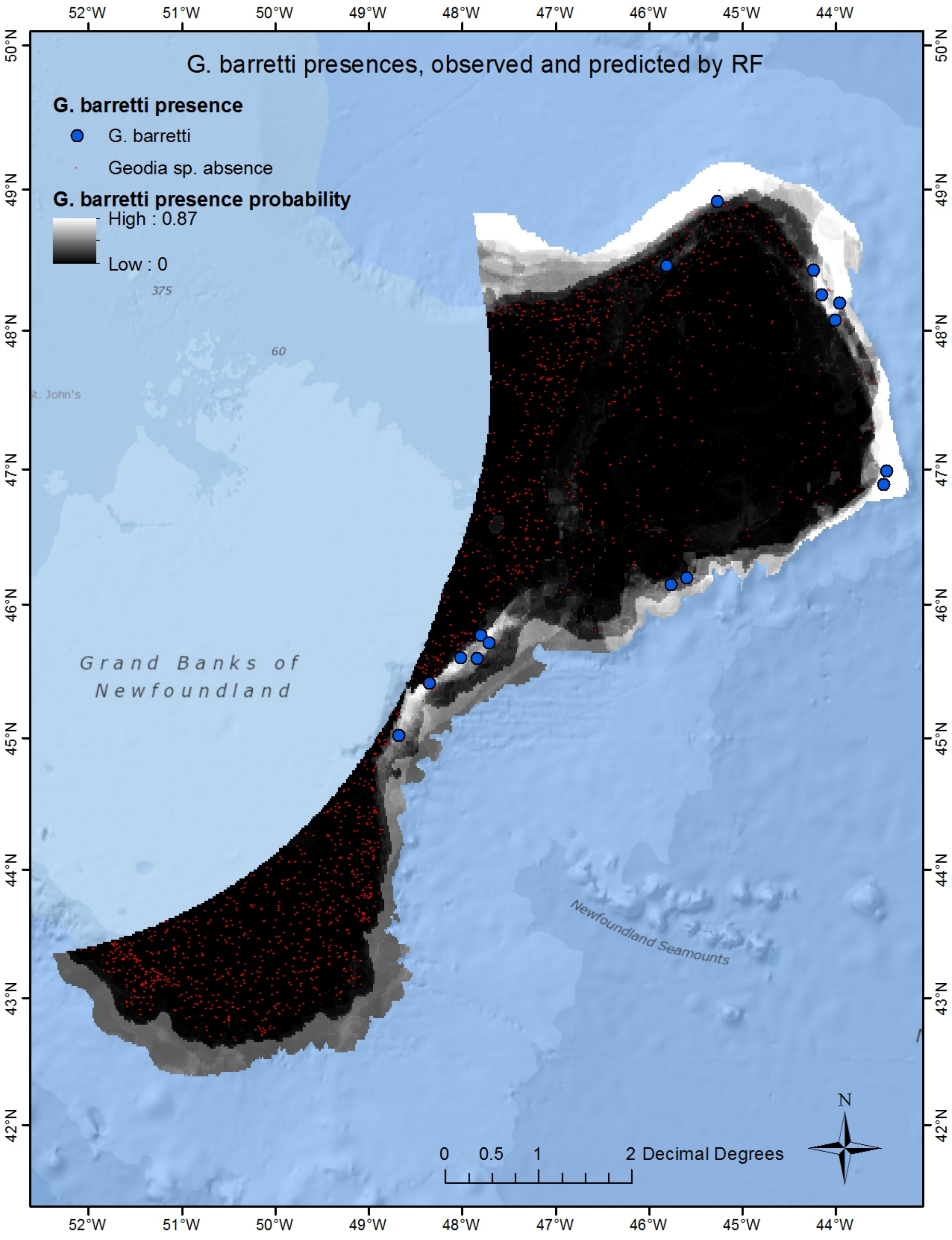

Figure 35. *G. barretti* locations in the FC study area, observed (blue circles) and predicted probability by the RF model (greyscale).

G. phlegraei

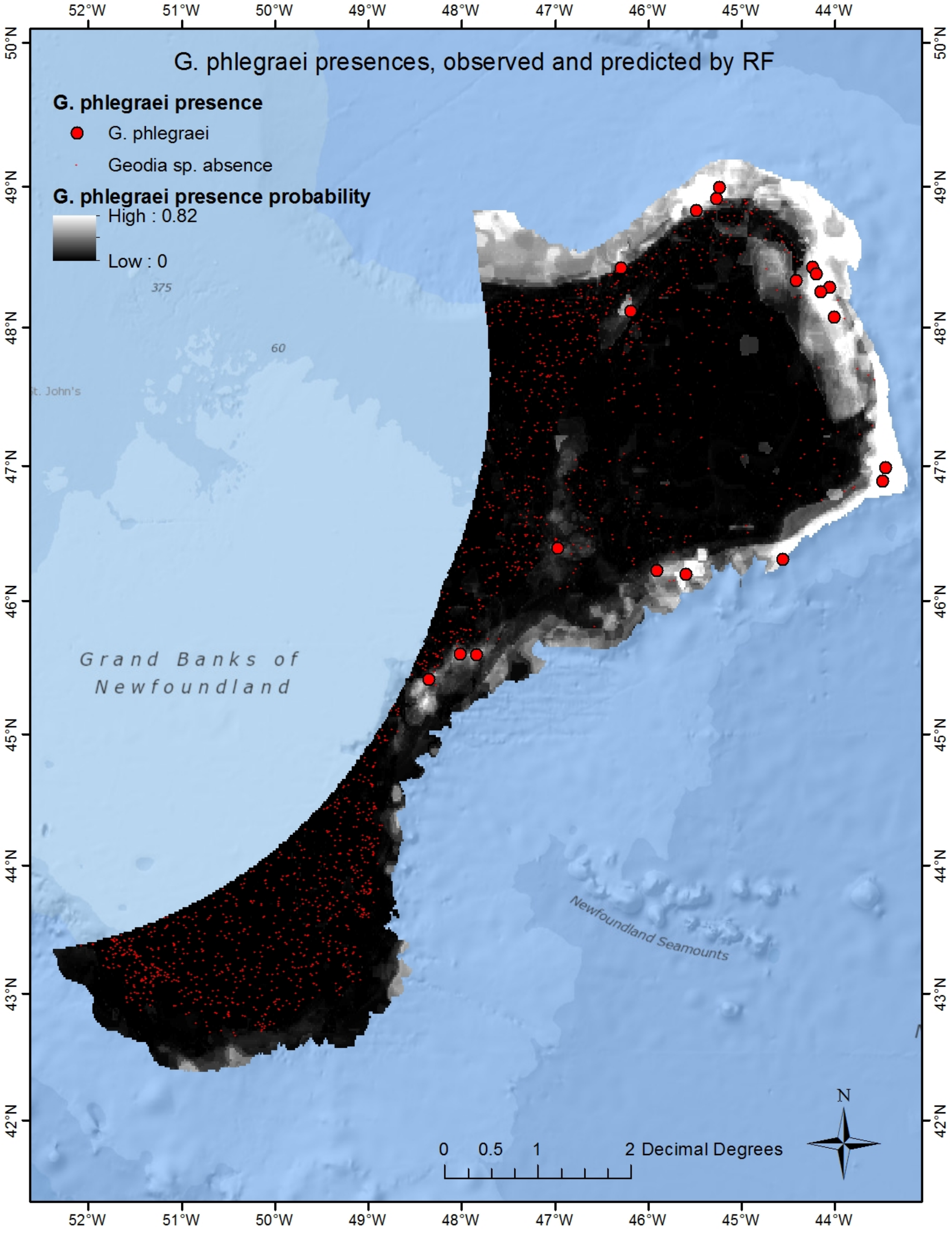

Figure 36. *G. phlegraei* locations in the FC study area, observed (red circles) and predicted probability by the RF model (greyscale).

Sponge Grounds

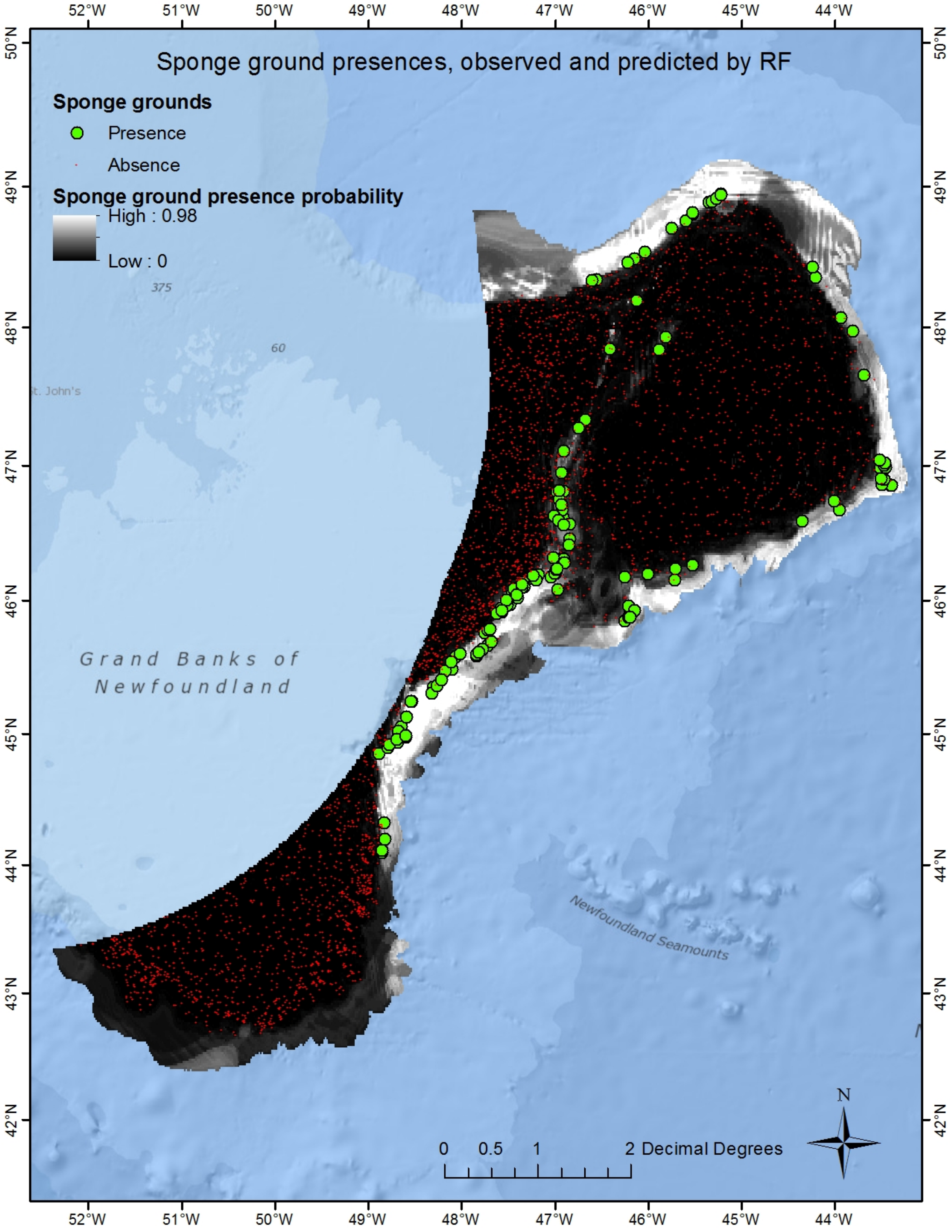

Figure 37. Sponge ground locations in the FC study area, observed (green circles) and predicted probability by the RF model (greyscale).

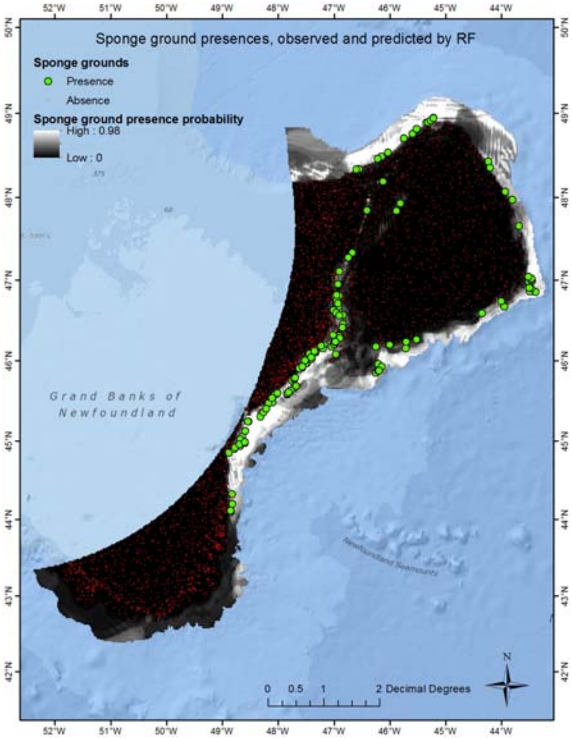

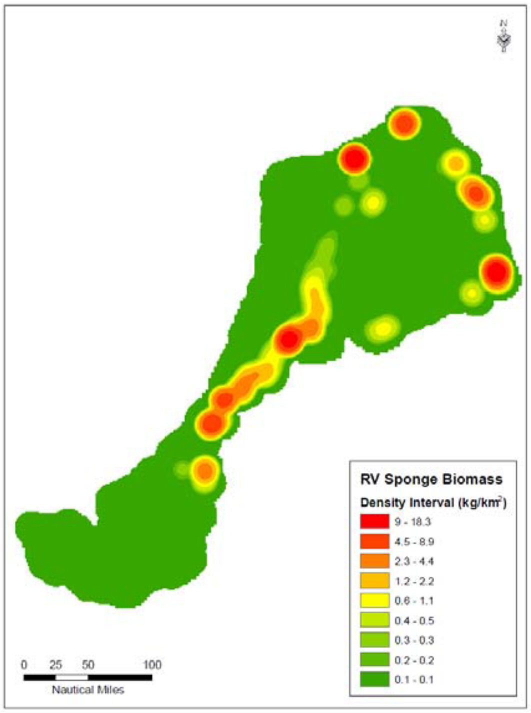

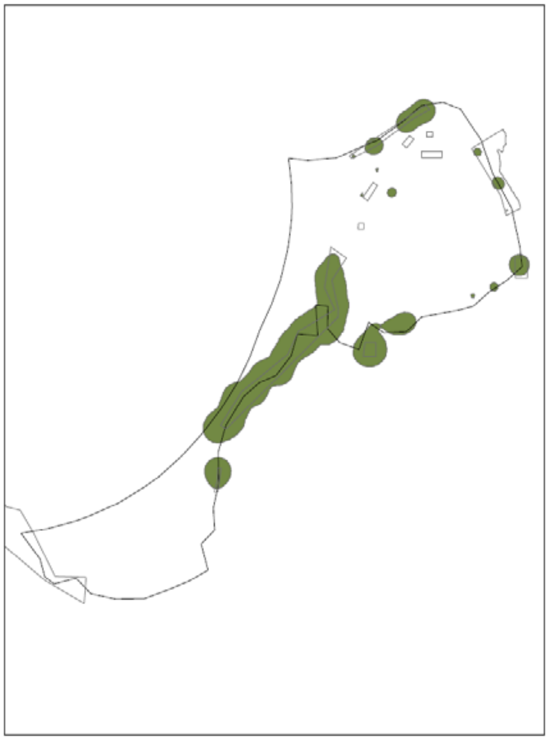

Figure 38. Comparison of predicted sponge grounds with kernel biomass. **Upper left panel.** Sponge ground locations in the FC study area, observed (green circles) and predicted probability by the RF model (greyscale). **Upper right panel.** Sponge kernel biomass (displayed using a geometric distribution in kg/km^2) in the FC study area estimated from Spanish/EU research vessel catches (Kenchington et al. 2012). **Lower panel.** Sponge grounds as identified in the FC study area by NAFO based on a kernel biomass of 75 kg/km in the FC study area estimated from Canadian (DFO) and EU-Spanish research vessel catches. Areas closed by NAFO to protect sponges and other vulnerable marine ecosystems are indicated in outline in relation to the regulatory area.

ACKNOWLEDGEMENTS

This project was funded by Fisheries and Oceans Canada through the Strategic Program for Ecosystem-Based Research and Advice (SPERA) program established to provide scientific advice relevant to the implementation of an ecosystem-based approach to management: *Evaluation of geospatial tools for delineating concentrations of deep-sea corals and sponges* to E. Kenchington and A. Knudby (2012/13). We thank Dr. Janelle Curtis (Pacific Biological Station, DFO) and Neil Ollerhead (Northwest Atlantic Fisheries Centre, DFO) for their thoughtful reviews.
